# BTN3A3 evasion promotes the zoonotic potential of influenza A viruses

**DOI:** 10.1038/s41586-023-06261-8

**Published:** 2023-06-28

**Authors:** Rute Maria Pinto, Siddharth Bakshi, Spyros Lytras, Mohammad Khalid Zakaria, Simon Swingler, Julie C Worrell, Vanessa Herder, Kerrie E Hargrave, Margus Varjak, Natalia Cameron-Ruiz, Mila Collados Rodriguez, Mariana Varela, Arthur Wickenhagen, Colin Loney, Yanlong Pei, Joseph Hughes, Elise Valette, Matthew L Turnbull, Wilhelm Furnon, Quan Gu, Lauren Orr, Aislynn Taggart, Ola Diebold, Chris Davis, Chris Boutell, Finn Grey, Edward Hutchinson, Paul Digard, Isabella Monne, Sarah K Wootton, Megan K L MacLeod, Sam J Wilson, Massimo Palmarini

**Affiliations:** 1 MRC–University of Glasgow Centre for Virus Research (CVR), Glasgow, UK; 2 School of Infection and Immunity, University of Glasgow, UK; 3 Department of Pathobiology, University of Guelph, Guelph, Ontario, Canada; 4 Istituto Zooprofilattico Sperimentale delle Venezie (IZSVe), Legnaro, Italy; 5 The Roslin Institute, University of Edinburgh, Edinburgh, UK

## Abstract

Spillover events of avian influenza A viruses (IAV) to humans could represent the first step of a future pandemic^1^. Multiple factors limiting the transmission and replication of avian IAV in mammals have been identified. There are several gaps in our understanding in order to predict which virus lineages are more likely to cross the species barrier and cause disease in humans^1^. Here, we identified human BTN3A3 (butyrophilin subfamily 3 member A3)^2^ as a potent inhibitor of avian but not human IAV. We determined that BTN3A3 is expressed in human airways and its antiviral activity evolved in primates. We show that BTN3A3 restriction acts primarily at the early stages of the virus life cycle by inhibiting avian IAV RNA replication. We identified residue 313 in the viral nucleoprotein (NP) as the genetic determinant of BTN3A3 sensitivity (313F, or rarely 313L in avian viruses) or evasion (313Y or 313V in human viruses). However, avian IAV serotypes, such as H7 and H9, that spilled over into humans evade BTN3A3 restriction. In these cases, BTN3A3 evasion is due to substitutions (N, H or Q) in NP residue 52 that is adjacent to residue 313 in the NP structure^3^. Thus, sensitivity or resistance to BTN3A3 is another factor to consider in the risk assessment of the zoonotic potential of avian influenza viruses.

Influenza A viruses (IAV) cause a substantial global health burden and circulate both in humans and animal species including domestic poultry, pigs, dogs and horses. Wild aquatic birds are the main natural reservoir of IAV^4^. Ducks, shorebirds, gulls and other waterbirds harbour 16 haemagglutinin (HA) and 9 neuraminidase (NA) subtypes^5^. IAV from wild bird reservoirs can infect economically important birds such as chickens, turkeys, quail and other gallinaceous species, or domestic waterfowl^4^. The population density of domestic birds and the direct exposure to infected birds facilitates avian IAV spillovers into susceptible mammals, including humans. Avian IAV can also further reassort (i.e. exchange genome segments) with viruses established in susceptible species resulting in the rich IAV genetic diversity. In humans, the influenza pandemics of 1918, 1957, 1968 and 2009 were all caused by viruses containing genomic segments of avian origin^6^.

Occasionally, spillover of avian IAV into humans may result in severe or even lethal disease^7^. For example, in 2013 an H7N9 variant resulting from multiple reassortment events of different avian viruses^8^ caused more than 600 human deaths^9^ and has since re-emerged in different epizootic clusters^10^. These spillover events are not typically followed by extensive human-to-human transmission chains, but they are a risk to global health as they could enable the first step towards human adaptation and the generation of pandemic IAV strains^1^.

Multiple barriers have been identified that hamper avian IAV transmission and adaptation in humans^11^. These include virus HA receptor binding specificity^12^, higher HA pH fusion^13,14^, increased efficiency of the virus polymerase in human cells^15^, length of the NA stalk^16,17^, and sensitivity to the host antiviral factor Mx1/MxA^11,18,19^.

Despite the progress made in the last two decades, gaps remain in understanding what allows certain avian IAV subtypes/lineages to spill over in humans. In this study, we aimed to identify (i) host genetic barriers to avian IAV replication in human cells and (ii) IAV genetic signatures that can be directly correlated with their zoonotic potential.

## BTN3A3 specifically restricts avian IAV

**Figure 1:**
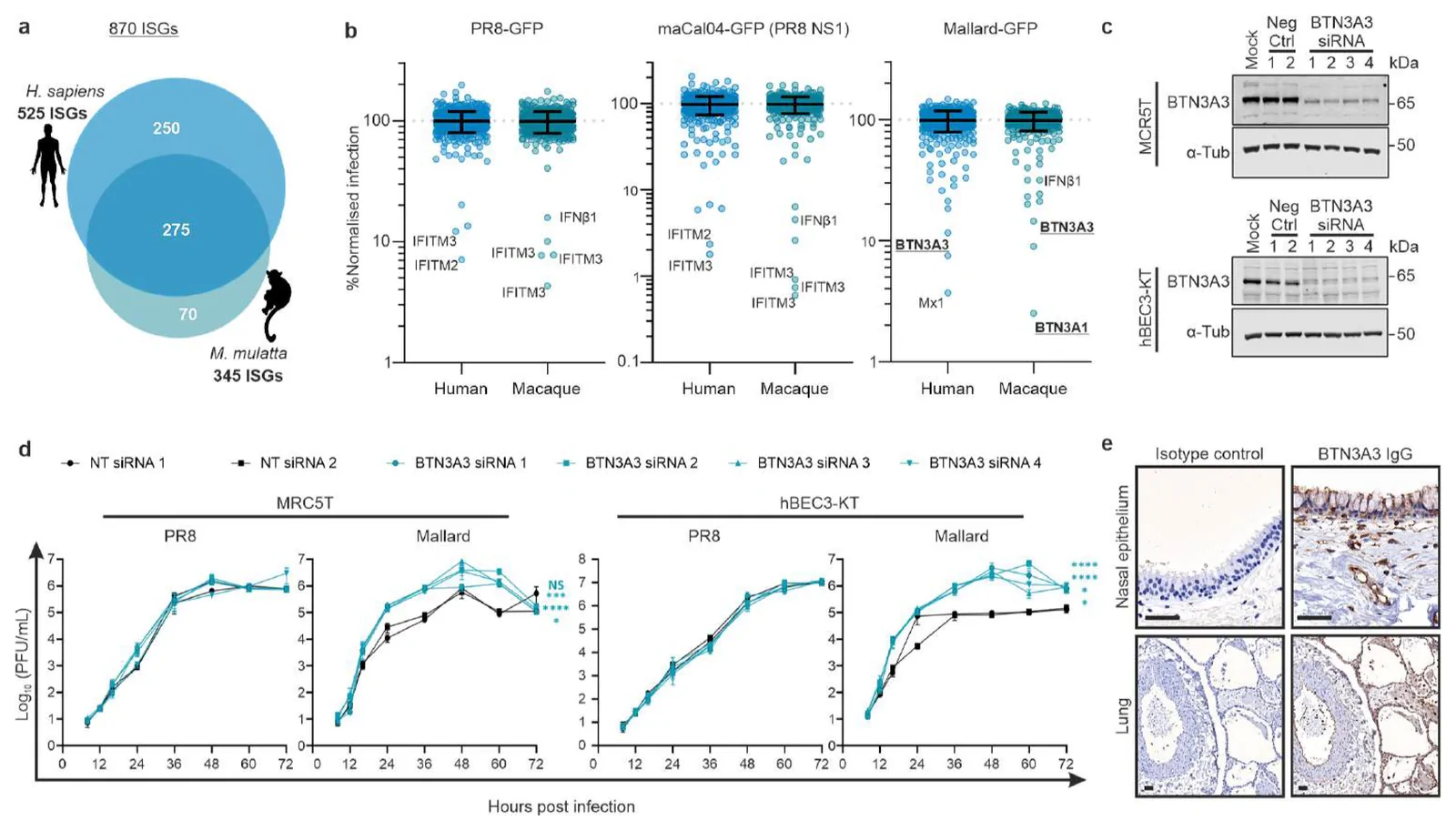
BTN3A3 inhibits avian IAV. **a** Schematic representation of the ISG libraries used in the screens. **b**, Graphs showing the results of normalised infections (% median of overall library) of cells expressing individual ISGs with the indicated viruses. ISGs restricting virus replication are labelled. Each dot represents an individual ISG of libraries illustrated in panel a (870 ISGs in total). **c**, Expression and effective siRNA-driven knock-down of BTN3A3 in MRC5T and hBEC3-KT. Cells were transfected with scrambled (Neg ctrl) or BTN3A3-targetting siRNAs and protein levels in the resulting cell lysates were assessed by western-blot. α-Tubulin was used as loading control. For gel source data, see Supplementary Figure 1. **d**, Replication kinetics of PR8 and Mallard in siRNA-treated MRC5T and hBEC3-KT. Data are mean +/- standard error of the mean (SEM) of 3 independent experiments (each using two technical replicates). Statistical significance between groups was measured by a 2-way ANOVA. Comparisons were made between area under the curve of the different BTN3A3 siRNA treatment conditions and the average of the two negative controls. NS-Non-significant, *p≤0.05, **p≤0.01, ***p≤0.001, ****p≤0.0001. **e**, Photomicrographs of human nasal epithelium and lung sections subjected to immunohistochemistry. Cells expressing BTN3A3 (shown in brown) are found along the entire nasal epithelium and in endothelial cells, immune cells as well as pneumocytes. Bars represent 50μm.

We focused on the host type-I interferon (IFN) response as it is one of the key host antiviral innate immune mechanisms and a barrier for virus cross-species transmission^20^. IFN acts through the activation of hundreds of interferon stimulated genes (ISGs), some of which have antiviral properties^20^. Hence, we first aimed to identify human ISGs that contribute to IAV host tropism. We performed arrayed expression screening^21^ of 870 human and macaque ISGs (Fig. 1a) using three different GFP-tagged recombinant IAV strains: A/Puerto Rico/8/1934 (henceforth PR8, a human laboratory adapted H1N1 strain), A/California/04/2009 (Cal04, a mouse-adapted 2009 pandemic [pdm09] H1N1 strain) and A/mallard/Netherlands/10-Cam/1999 (Mallard, an H1N1 avian strain). These screens (Fig. 1b, Supplementary Table 1) identified ISGs previously shown to be antiviral against IAV, such as IFITM2, IFITM3 and Mx1^11,22–24^. Interestingly, two other ISGs, BTN3A1 and BTN3A3, inhibited the avian virus (Mallard), but not the mammalian viruses (PR8 or Cal04).

**Extended Data Fig. 1:**
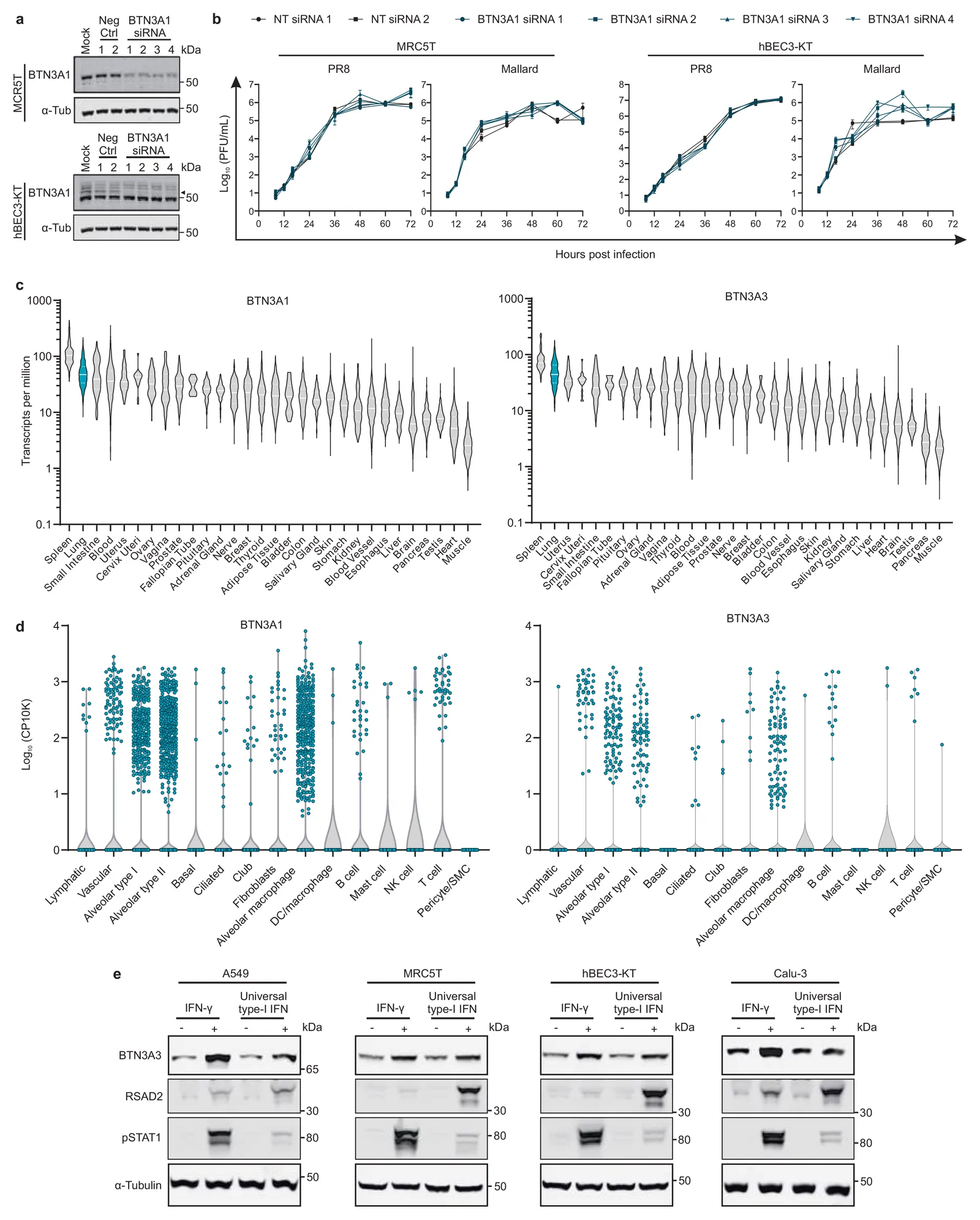
Activity and expression of BTN3A1 and BTN3A3 in cell lines and human tissues. **a**, siRNA knock-down of BTN3A1 in MRC5T and hBEC3-KT. Cells were transfected with scrambled (Neg ctrl) or BTN3A1-targetting siRNAs, and protein levels in the resulting cell lysates were assessed by western blotting. α-Tubulin was used as loading control. Arrows indicate the band corresponding to BTN3A1. For gel source data, see Supplementary Figure 1. **b**, Graphs showing the replication kinetics of PR8 and Mallard in siRNA-treated MRC5T and hBEC3-KT cells. Cells were infected with a MOI of 0.001, supernatants were collected at the indicated times post infection and viruses titrated by plaque assay. Data are mean +/- SEM of 3 independent experiments (each using 2 technical replicates). Statistical significance between groups was measured by a 2-way ANOVA. Comparisons were made between area under the curve of the different BTN3A1 siRNA treatment conditions and the average of the two negative controls. No statistically significant differences were found. **c**, Organ-dependent bulk tissue gene expression. Lung samples are highlighted in blue. **d**, Lung single cell tissue expression. Data in **c** and **d** were obtained from the GTEx Portal (www.gtexportal.org). **e**, Western blotting of cell lysates obtained from A549, MRC5T, hBEC3-KT and Calu-3 treated with either IFN-γ or universal type-I IFN. Treatment with IFN was for 16h in A549, MRC5T, hBEC3-KT and for 24h in Calu-3 cells. pSTAT1 and RSAD2/Viperin were used as IFN induction controls and α-Tubulin as loading control. For gel source data, see Supplementary Figure 1.

In order to validate these hits, we carried out loss of function experiments. Constitutive expression of BTN3A3 was detected in primary immortalised human foetal lung fibroblasts (MRC5T) and human bronchial epithelial cells (hBEC3-KT) (Fig. 1c). siRNA-mediated knockdown of BTN3A3 in these cells resulted in improved replication of the avian Mallard strain, while it did not affect the growth kinetics of PR8 (Fig. 1d). Equivalent knockdown experiments targeting BTN3A1 did not show a significant difference in avian IAV’s replication (Extended Data Fig. 1a-b). By immunohistochemistry we showed that BTN3A3 is constitutively expressed in the healthy respiratory tract of human donors, both in the upper and lower respiratory tract. BTN3A3 expression was detected in ciliated cells within the nasal epithelium and bronchioli, type I and II alveolar epithelial cells and alveolar macrophages (Fig. 1e). Indeed, available data from human transcriptomic profiles (GTEx consortium) suggest that the lungs have the second highest expression levels of BTN3A3 across 30 tissues analysed (Extended Data Fig. 1c-d). Importantly, we found that a variety of human lung cell lines displayed constitutive expression of BTN3A3, which can then be further upregulated by either type-I or type-II IFN (Extended Data Fig. 1e).

**Figure 2:**
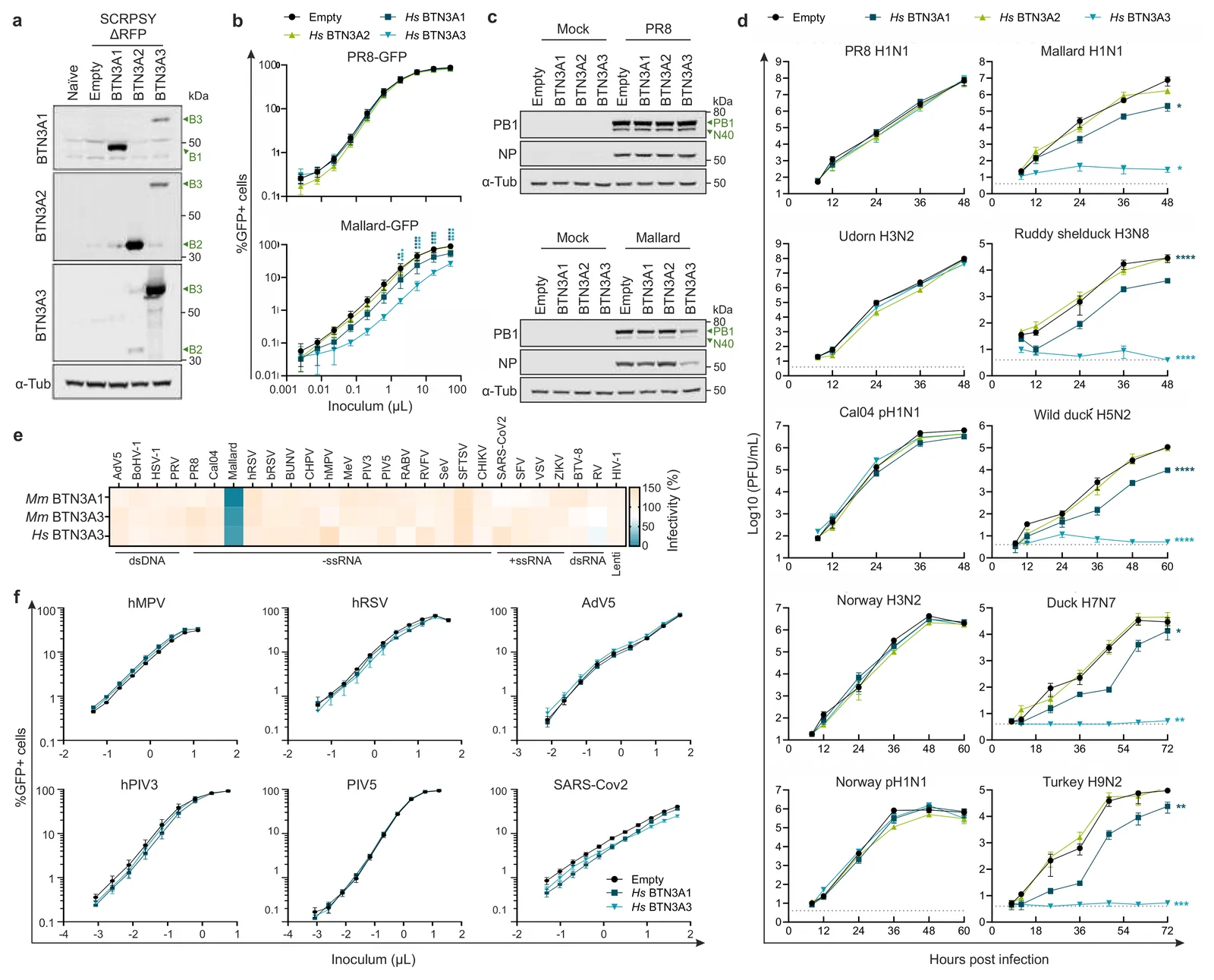
Specificity of BTN3s restriction. **A**, Western blotting of cell lysates obtained from A549 cells stably expressing human BTN3A1 (B1), BTN3A2 (B3) or BTN3A3 (B3). **b**, BTN3-overexpressing A549 cells were challenged with serial dilutions of GFP-tagged PR8 or Mallard for 10 hours (h). Upon trypsinisation and fixation, percentage of GFP-positive cells was measured by flow cytometry. Data are mean +/- SEM of 3 independent experiments (each using two technical replicates). Multiple comparisons between each BTN3-expressing and empty control cells was performed using a one-way ANOVA. **c**, BTN3A-expressing A549 cells were infected with PR8 or Mallard WT viruses at MOI 3 for 6h. Viral protein expression was measured by detection of PB1 (and PB1-N40) and NP by western blot. **d**, Replication kinetics of a panel of human (left panels) and avian (right panels) viruses (MOI= 0.001) in A549-Empty and BTN3A1 or BTN3A3-expressing cells. Data represent mean +/- SEM of 3 independent experiments (each using 2 technical replicates). Comparisons were made between area under the curve of the different BTN3-expressing and empty cells using a 2-way ANOVA. **e**, Heatmap showing normalised infection of cells expressing BTN3A1 and BTN3A3 for a panel of indicated viruses (see Methods for details). **f**, Human BTN3A1 and BTN3A3 overexpressing A549s were challenged with increasing volumes of the indicated GFP-tagged viruses. Cells were fixed at 10h post infection (hpi) for PR8 and Mallard and 16 hpi for the remaining viruses. Percentage of GFP-positive cells was measured by flow cytometry. Data are mean +/- SEM of 3 independent experiments (each using two technical replicates). Statistical annotations in this figure: NS =Non-significant, *p≤0.05, **p≤0.01, ***p≤0.001, ****p≤0.0001. Samples in (a) and (c) were derived from the same experiment and gels were processed in parallel. For gel source data, see Supplementary Figure 1.

The butyrophilin gene superfamily has undergone complex duplication events over its evolutionary history with subfamily 3 comprising three primate-specific paralogues: BTN3A1-3^2,25^. We tested the antiviral activity of overexpressed human BTN3A1, BTN3A2 and BTN3A3 in A549 cells (Fig. 2a). Infection of BTN3-overexpressing cells using GFP-tagged and untagged viruses showed that PR8 was not sensitive to any of the human BTN3 proteins. However, BTN3A1 and BTN3A3 were successful at restricting Mallard (the latter being more effective at restriction) whilst BTN3A2 showed no antiviral effects (Fig. 2b-c). Next, we assessed the antiviral effects of the BTN3 proteins against a wider panel of IAV strains. These included human laboratory-adapted viruses, human clinical isolates and various avian IAV. None of the human viruses tested were sensitive to any of the BTN3A proteins (Fig. 2d, left panels). However, the replication kinetics of all avian viruses was restricted approximately 10-fold by BTN3A1. Restriction of avian viruses by BTN3A3 was even greater with viral titres barely reaching the limit of detection and showing a decrease of up to 100,000-fold (Fig. 2d, right panels).

We further tested the antiviral specificity of BTN3A1 and BTN3A3 against an additional 24 viruses, including dsDNA, ssRNA and dsRNA viruses. In this screening process, the avian IAV Mallard was the only virus substantially inhibited by BTN3A1 and BTN3A3 (Fig. 2e). Further validation assays against a panel of human respiratory viruses confirmed the specificity of this restriction factor against avian IAV (Fig. 2f).

## BTN3A3 evolution

**Extended Data Fig. 2:**
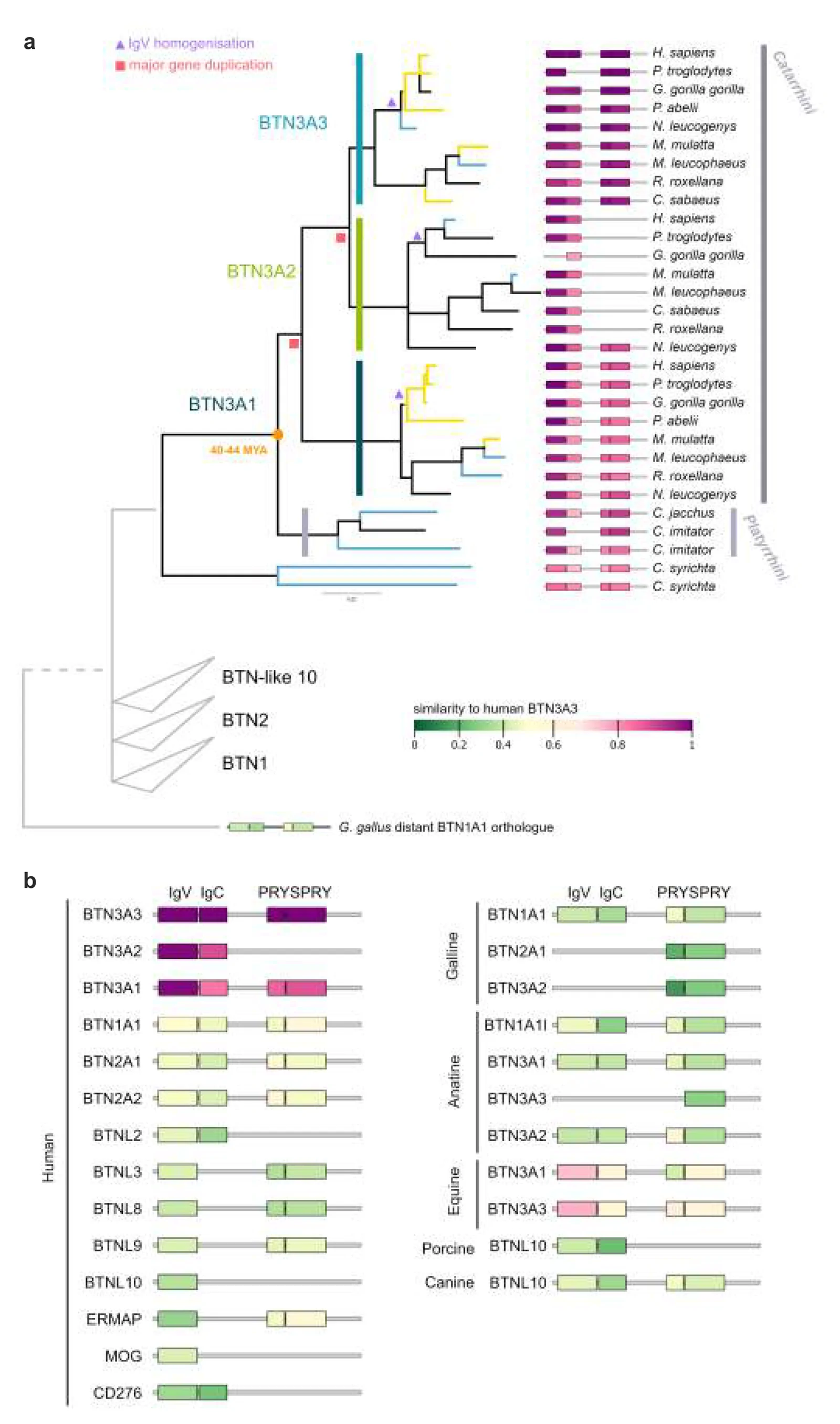
Evolution of BTNs **a**, Maximum likelihood phylogeny of concatenated protein domain coding sequences of the *Haplorrhini* BTN3 genes (K2P+G4 substitution model). Nodes with bootstrap support below 60 have been collapsed. Branches confirmed to have or not have anti-avian IAV activity (described in b) are highlighted in yellow and blue, respectively. Branches not tested were kept black. Relation to more distant tested homologues and orthologous/paralogous gene families are shown as a schematic in grey. IgV homogenisation events, major gene duplications and gene subfamilies are annotated on the phylogeny. Presence of each of the four protein domains (IgV, IgC, PRY and SPRY) is annotated on the right of each tree tip and coloured by pairwise amino acid similarity to the respective domain of the human BTN3A3. Species names and taxonomic classification is annotated on the right. The median divergence time between *Catarrhini* and *Platyrrhini* was retrieved from TimeTree (http://timetree.org/). **b**, Schematic representation of domain organisation and sequence similarity of the indicated proteins.

We then examined the origin of anti-avian IAV activity in the BTN3 gene family. Phylogenetic analysis of the BTN3A genes of the *Haplorrhini* sub-order (tarsier, monkeys, apes, and humans) indicated that BTN3A1-3 originated through two successive duplications after the split between the new world monkey lineage (*Platyrrhini*) and the old world monkey/ape lineage (*Catarrhini*) around 40 – 44 million years ago (Extended data Fig. 2a)^26^. Domain detection analysis showed that the majority of BTN3A1 and BTN3A3 genes have a consistent domain organisation with one set of N-terminal IgV and IgC domains followed by a PRYSPRY domain, while BTN3A2 genes have lost their PRYSPRY domain (with the exception of *Nomascus leucogenys* BTN3A2) (Extended Data Fig. 2a-b).

**Extended Data Fig. 3:**
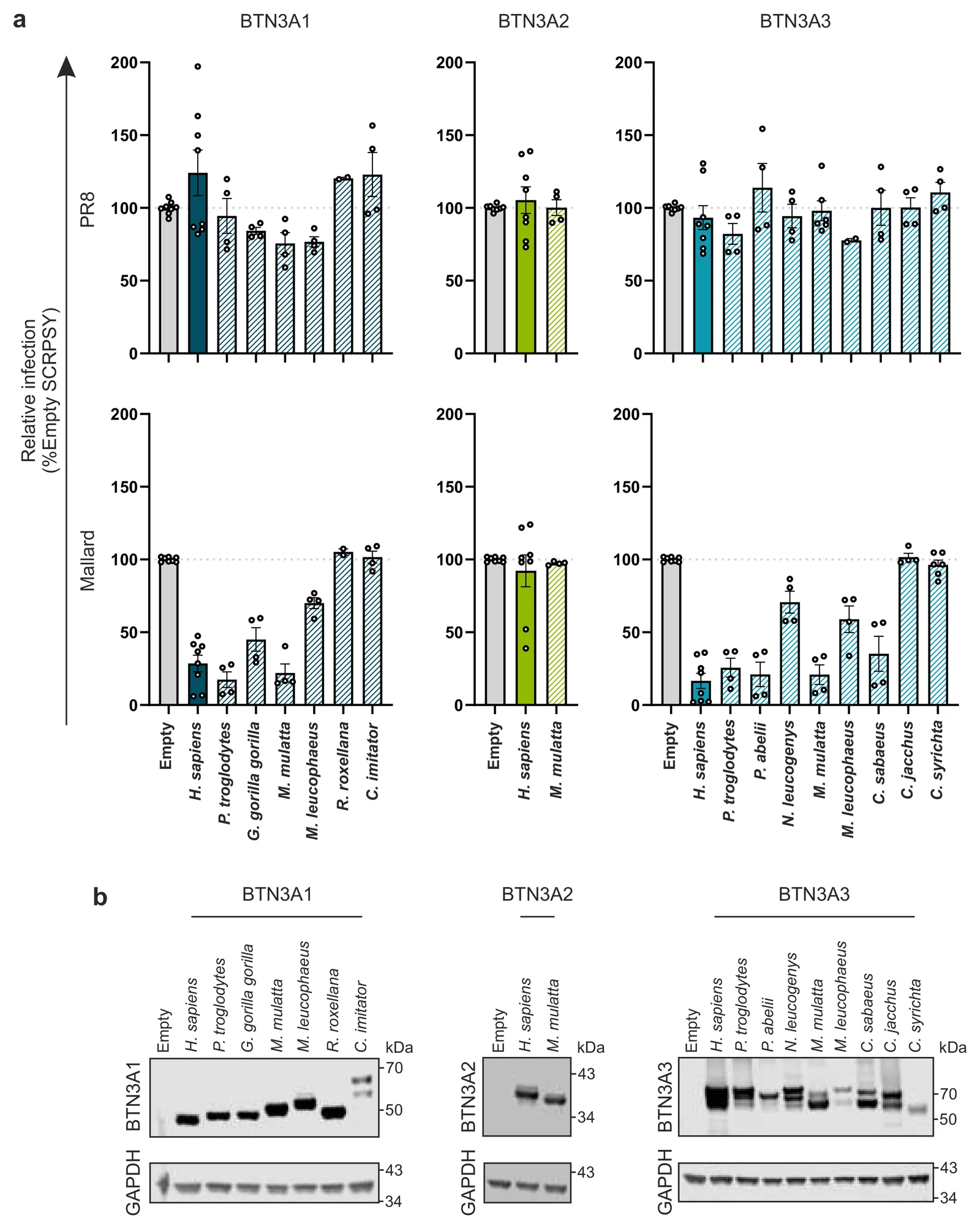
Evolution of antiviral activity of BTNs. **a**, A549 cells were transiently transduced with SCRPSY lentiviruses expressing the indicated BTN proteins and challenged with PR8- or Mallard-GFP. Eight hours post infection, the percentage of GFP-positive cells was measured by flow cytometry. Data are mean +/- SEM of 2 independent experiments which both gave similar results. **b**, Western blotting of cell lysates obtained from A549 cells transiently transduced with SCPRSY lentiviruses expressing different BTN proteins. GAPDH was used as loading control. For gel source data, see Supplementary Figure 1.

**Extended Data Fig. 4:**
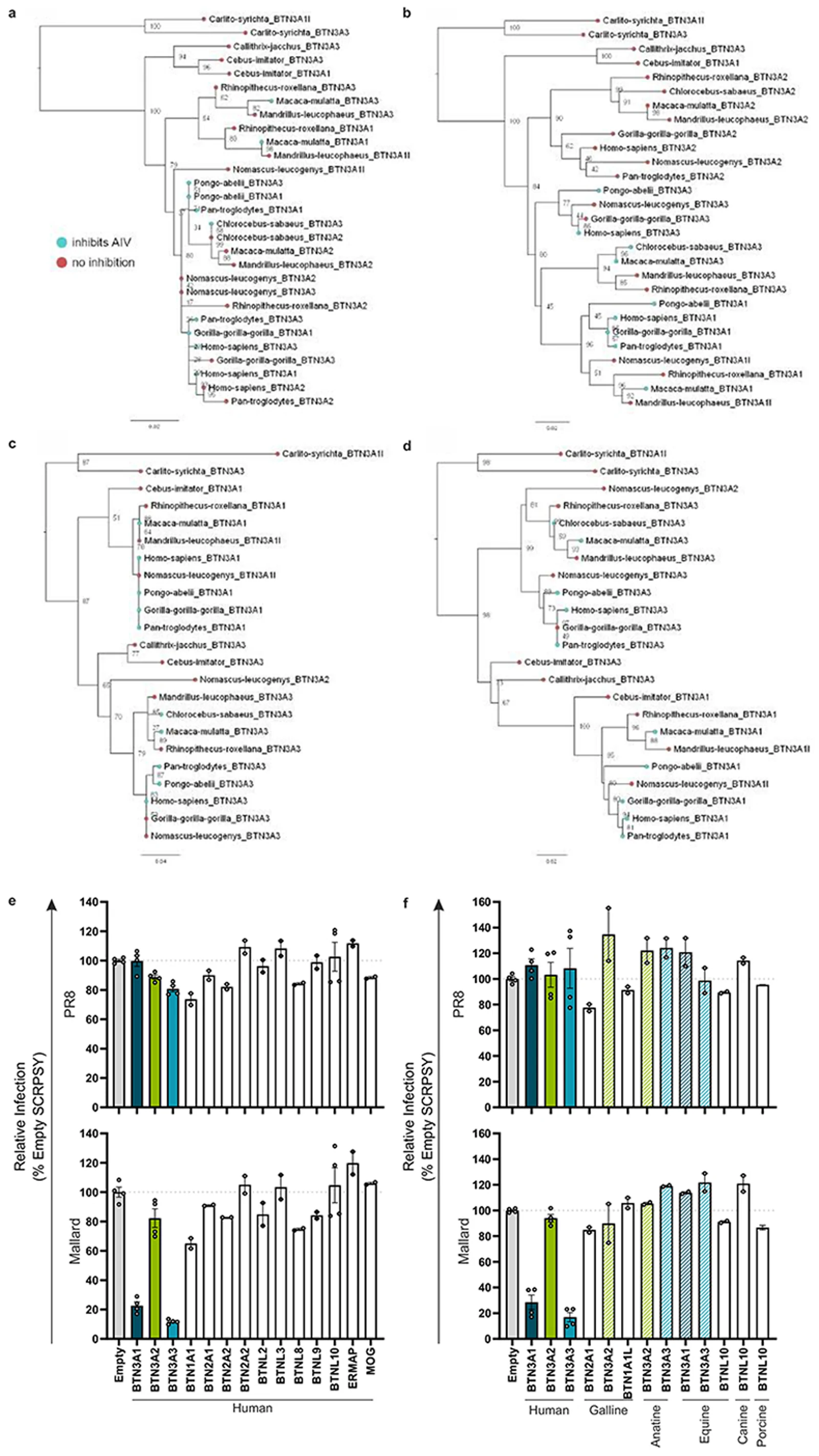
Phylogeny of BTN3 domains and antiviral activity of BTN orthologues and paralogues. **a-d**, maximum likelihood *Haplorrhini* BTN3 gene coding sequence phylogenies of separate domains: IgV (**a**), IgC (**b**), PRY (**c**), SPRY (**d**) under a K2P+G4 substitution model. Trees are rooted at the *C. syrichta* branch and node confidence values (10,000 bootstrap replicates) are annotated on each node. Tip shapes are coloured by whether each gene exhibits anti-AIV activity (consistent with Fig. 3a). Phylogenies were visualised using FigTree. **e-f**, A549 cells were transiently transduced with SCRPSY lentiviruses expressing the indicated BTN proteins and challenged with PR8- or Mallard-GFP. Eight hours post-infection, percentage of RFP-positive cells and its subpopulation of GFP-positive cells was measured by flow cytometry. Data are mean +/- SEM of 2 independent experiments which both gave similar results. Detection of these proteins was not possible using commercially available antibodies, due to their genetic divergence compared to human BTN3A1-3. Therefore, tagging of these BTN genes was attempted by introducing a C-terminal FLAG. Despite their protein expression being successfully detected using an anti-FLAG antibody, the addition of FLAG to human BTN3A3 and other BTN genes resulted in the abolishment of their antiviral activity. Cloning of the genes indicated above was conducted in the same way as those constructs shown in Extended Data 3.

Data obtained by transient transduction of A549 cells with BTN3A-expressing lentiviruses followed by viral challenge suggests that the antiviral phenotype of BTN3A3 was gained after the *Platyrrhini*-*Catarrhini* split, consistent with the two duplication events. Humans, chimpanzees (*Pan troglodytes*), gorillas (*Gorilla gorilla gorilla*), orangutans (*Pongo abelii*), macaques (*Macaca mulatta*) and green monkeys (*Chlorocebus sabaeus*) all have at least one BTN3A1/3 gene capable of inhibiting Mallard viral replication (Extended data Fig. 3). Our phylogenetic analysis which considers each *Haplorrhini* BTN3 protein domain individually is consistent with previously documented recombination and homogenisation of the IgV domain within the BTN3 *Catarrhini* gene subfamily^27^ (Extended Data Fig. 4a-d). This incongruence in the evolutionary history of these genes could explain the multiple gains/losses of antiviral function.

We also found that the closest orthologues to BTN3 from mammalian species with endemic IAV (canine, equine and porcine), the distant galline (*Gallus gallus*) and anatine (*Anas platyrhynchos*) BTN1 orthologues, and other human paralogues of the butyrophilin superfamily did not inhibit PR8 or Mallard, supporting a *Catarrhini* origin of BTN3 antiviral activity (Extended Data Fig. 4e-f).

## Determinants of resistance to BTN3A3

**Extended Data Fig. 5:**
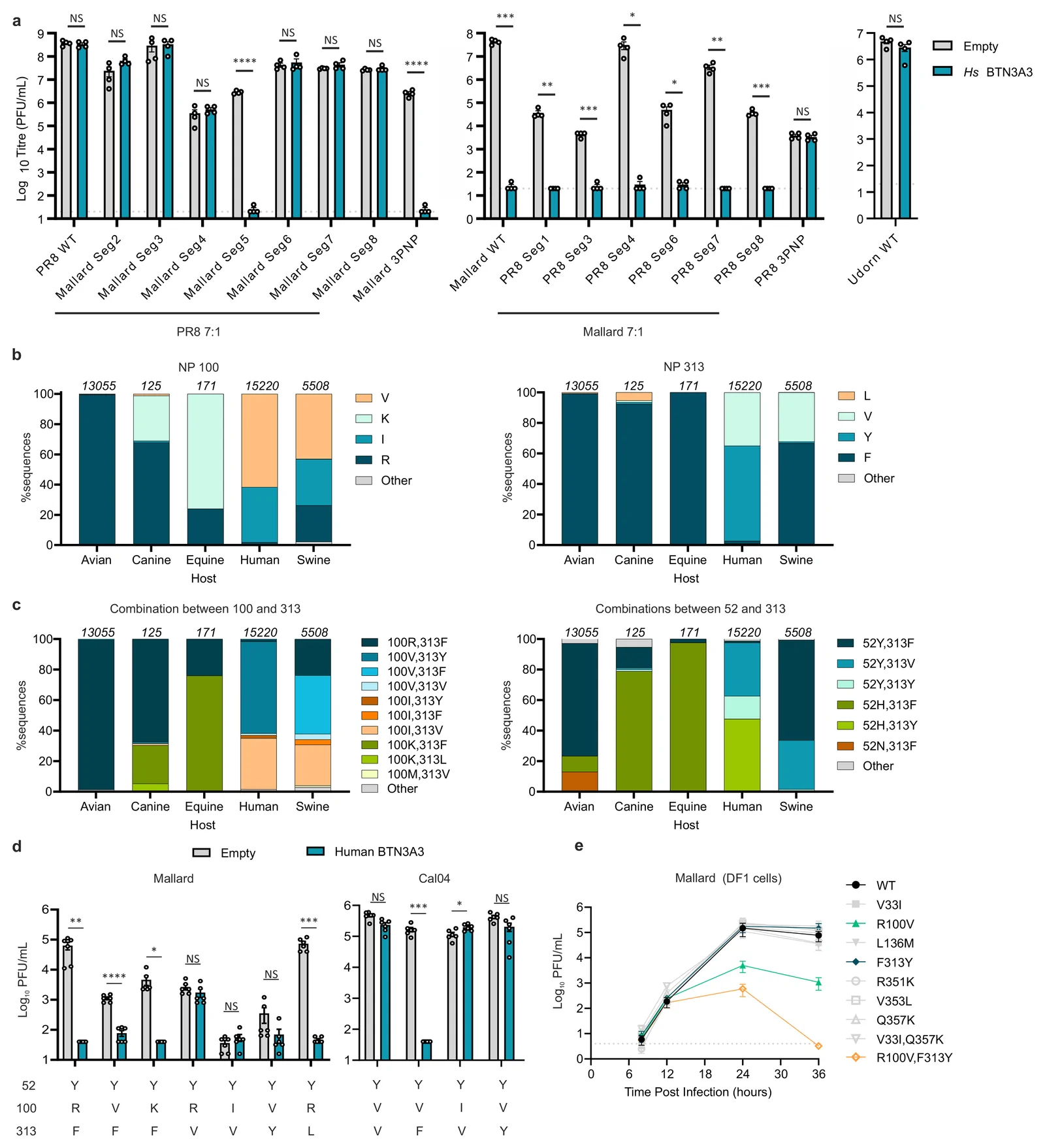
Determinants of BTN3A3 sensitivity. **a**, PR8:Mallard reassortants and Udorn wild type (WT) were used to perform plaque assays in MDCK cells expressing BTN3A3 and empty vector control cells. Note that reassortant PR8 7:1 Mallard seg 1, Mallard 7:1 PR8 seg 2 and Mallard 7:1 PR8 seg 5 failed to rescue. Reassortant PR8 Mallard 3PNP is formed by PR8 (segments 4, 6, 7 and 8) and segments 1, 2, 3 and 5 from Mallard. Conversely, reassortant Mallard PR8 3PNP contain segments 4, 6, 7 and 8 from Mallard and segments 1, 2, 3 and 5 from PR8. Data are mean +/- SEM of 2 technical replicates from 2 independent experiments. Statistical differences between cells expressing BTN3A3 and control cells were calculated using multiple t-tests and corrected for multiple comparisons using the Holm-Šídák method. NS-Non-significant, *p≤0.05, **p≤0.01, ***p≤0.001, ****p≤0.0001. **b**, Identity of amino acid residues in positions 100 (left) and 313 (right) of all NP proteins available in our dataset collected from GenBank. **c**, Identity of combinations between amino acid residues 100 and 313 (left) and 52 and 313 (right). **d**, Infectious virus titres obtained in A549-Empty and A549-BTN3A3 cells infected with either Mallard or Cal04 residues 100 and 313 mutants. Cells were infected with an MOI of 0.001 for 48h and viruses were titrated by plaque assay. Data are mean +/- SEM of 2 technical replicates from 3 independent experiments. Statistical analysis was carried out as in (a). **e**, Viral replication assays in avian cells were carried out in chicken fibroblasts (DF1 cells). Cells were infected an MOI of 0.001 for 48h. Infectious virus titres were determined by plaque assay. Data are mean +/- standard error of the mean (SEM) of 3 independent experiments (each using two technical replicates).

**Figure 3:**
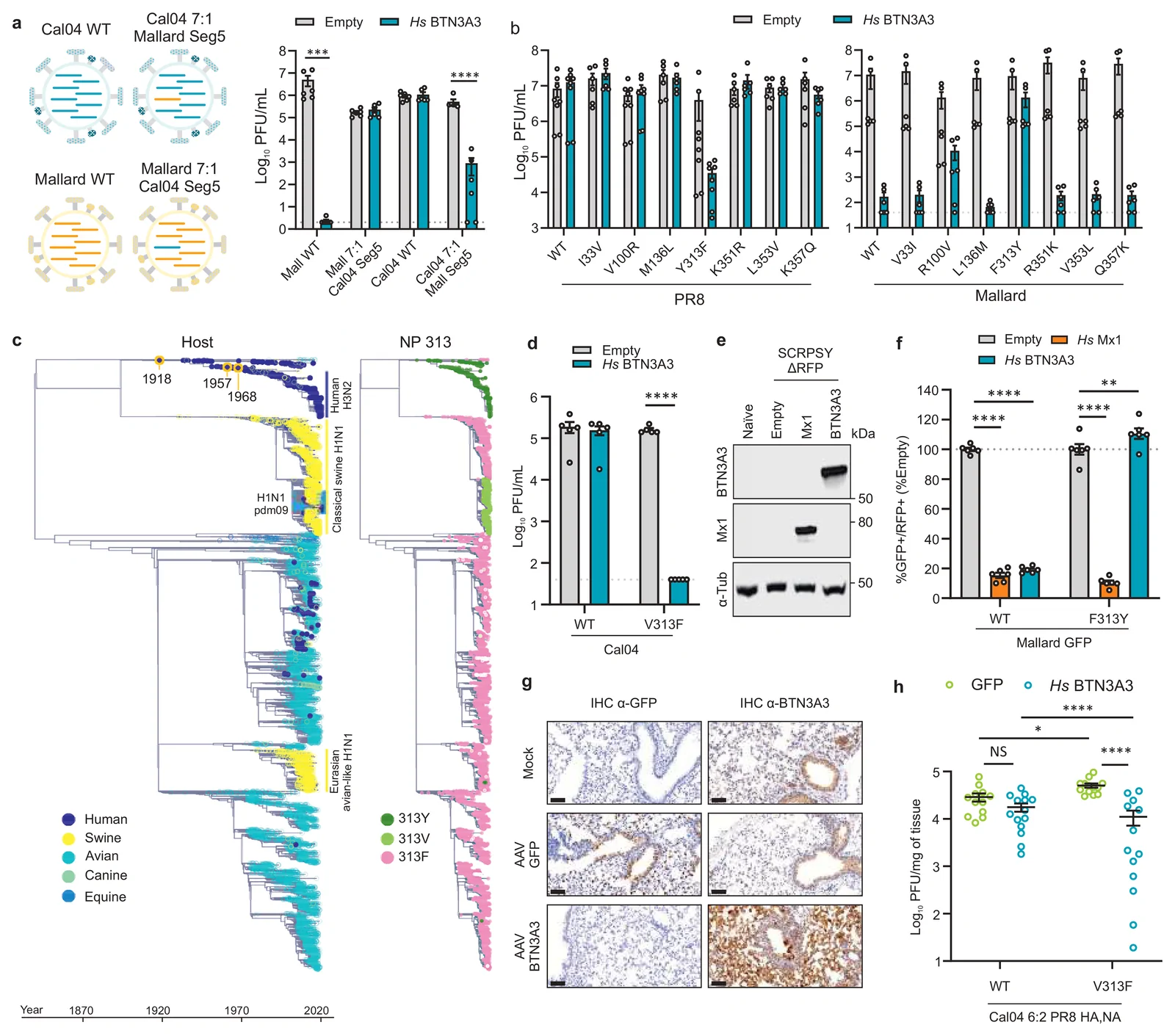
Determinants of BTN3A3 sensitivity. **a**, A549-Empty and BTN3A3-expressing cells were infected for 48 h with the schematised viruses (left; MOI 0.001), after which infectious titres were quantified by plaque assays (right). Data are mean +/- SEM of 3 independent experiments (each using 2 technical replicates). Multiple t-tests were performed using the Holm-Šídák method. **b**, PR8 and Mallard NP single mutants were used to infect A549-Empty and A549-BTN3A3 and titrated as in (a). **c**, Tip-dated maximum likelihood phylogeny of the filtered IAV NP coding sequence dataset (see Methods). Tip shapes are annotated by host (left) and 313 residue (right). Legend only shows residues occurring in more than 5% of the sequences. **d**, Cal04 WT and V313F mutant were used to infect A549-Empty and A549-BTN3A3. Virus quantification and statistical analysis were performed as in a. **e**, Western blotting of cell lysates obtained from A549 cells stably expressing human Mx1 or BTN3A3. Samples were derived from the same experiment and gels were processed in parallel. For gel source data, see Supplementary Figure 1. **f**, A549 overexpressing human Mx1 and BTN3A3 were infected with GFP-tagged Mallard WT or F313Y. Ten hpi cells were trypsinised, fixed and RFP- and GFP-positive cells were measured by FACS. Data are mean +/- SEM from 3 independent experiments (each using 2 technical replicates). Statistical differences were calculated as in (a). **g**, C57BL/65 mice were inoculated with PBS (mock), AAV-GFP or AAV-BTN3A3 for 3 weeks after which lungs were examined for GFP and BTN3A3 expression. Scale bars = 70μm. **h**, Three weeks post AAV-GFP or AAV-BTN3A3 treatment, mice were infected with Cal04 (6:2 PR8 HANA) WT or V313F mutant. Lung viral titres were assessed 3 days post infection. Data represent mean +/- SEM. Each group included 12 to 14 mice. Dots represent individual mice. Source data are available in Supplementary Table 9. Multiple comparisons were performed from a one-way ANOVA. Statistical annotations in this figure are defined as NS = Non-significant, *p≤0.05, **p≤0.01, ***p≤0.001, ****p≤0.0001.

To identify viral genetic determinants of BTN3A3 resistance, we engineered 7:1 PR8 : Mallard reassortants and tested their ability to form plaques in BTN3A3-overexpressing MDCK cells (Extended Data Fig. 5a). All PR8-based reassortants plaqued as efficiently in MDCK-Empty as in MDCK-BTN3A3 cells apart from those containing Mallard segment 5. The converse phenotype was seen in Mallard-based reassortants in which only viruses containing PR8 segment 5 (specifically Mallard 4:4 reassortant encoding PR8 viral ribonucleoprotein components – Mallard 3PNP) formed plaques in MDCK-BTN3A3 cells (Extended Data Fig. 5a). We were unable to rescue the single segment reassortant Mallard 7:1 PR8 Segment 5. Hence, we engineered the reciprocal 7:1 segment 5 reassortants between Mallard and Cal04. We assessed virus fitness in A549-Empty and A549-BTN3A3 cells and confirmed that Mallard segment 5 conferred BTN3A3 sensitivity (Fig. 3a). Segment 5 is monocistronic and encodes the viral nucleoprotein (NP)^28^. To identify the amino acid residue/s in NP determining the sensitivity or resistance to BTN3A3, we compared the NP sequences of the five human and five avian IAV tested in Fig. 2d. Human and avian NP sequences have conserved differences in residues 33, 100, 136, 313, 351, 353 and 357. Of these, positions 33, 100, 313 and 357 have been previously associated with avian-to-human transmission^29^. We rescued PR8 or Mallard single NP mutants and assessed their replication in A549-Empty or A549-BTN3A3. In PR8 background, we observed no reduction in virus yields in A549-BTN3A3 for any mutants except for PR8 Y313F. Conversely, on a Mallard background the R100V mutant resulted in partial loss of BTN3A3 sensitivity but a near complete evasion was seen for Mallard F313Y (Fig. 3b; Extended Data Fig. 5b). Therefore, we concluded that amino acid residue 313 is a key determinant of BTN3A3 sensitivity/resistance.

We next reconstructed a time-calibrated phylogenetic tree from a comprehensive set of more than 30,000 IAV NP amino acid sequences (Fig. 3c). While, human NP sequences have almost exclusively 313Y or 313V, all NP clades circulating in avian hosts, as well as the Eurasian avian-like H1N1 swine clade, predominantly exhibit a conserved 313F residue. Less than 1% of avian IAV NP sequences contain 313L, which also confers susceptibility to BTN3A3 (Extended Data Fig. 5b-d). Occurrence of the BTN3A3-resistant 313Y is specific to the human clade, originated from the H1N1 1918 pandemic which subsequently reassorted into 1957 H2N2 and further 1968 H3N2 pandemic and currently circulates seasonally. Precise dating of the original F313Y change is difficult due to the small number of pre 1918 IAV genomes available. However, recently sequenced genomes from the pandemic all code for NP 313Y^30^, suggesting that F313Y took place prior to or soon after human emergence of the 1918 H1N1 strain.

**Extended Data Fig. 6:**
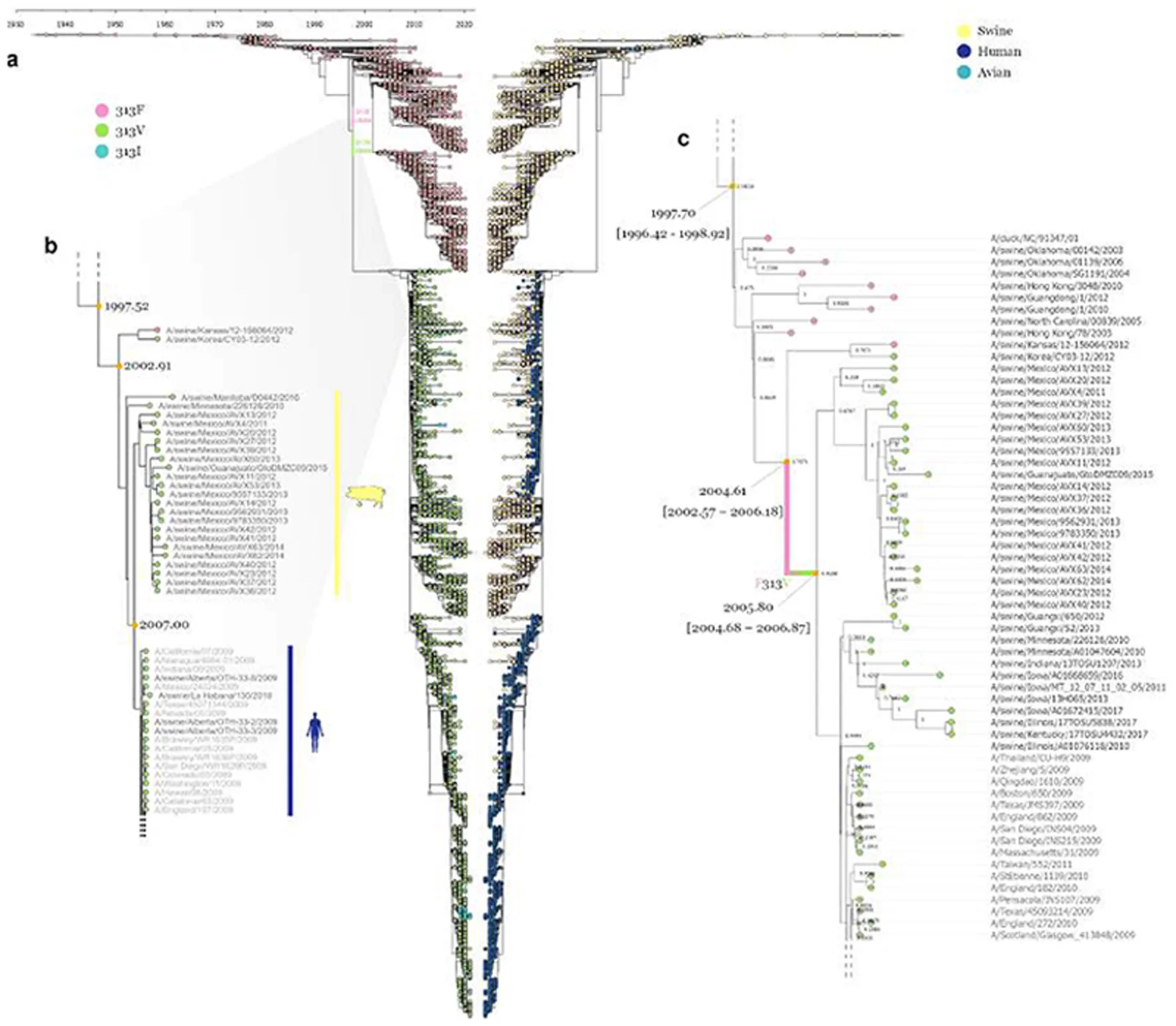
Molecular dating of the F313V NP substitution on the classical swine H1N1 lineage. **a**, Tip-dated maximum likelihood phylogeny of all classical H1N1 lineage NP sequences annotated by position 313 residue (left) and isolation host (mirrored tree, right). **b**, Zoomed in snippet of the part of the ML phylogeny shown in A where the F313V change has occurred. Tip shapes are coloured by 313 residue, estimated dates for key nodes are annotated, and strain names are shown on the right of the tips. **c**, Zoomed in snippet of the part of the BEAST maximum clade credibility phylogeny where the F313V change has occurred. Tip shapes are coloured by 313 residue, median node age and 95% highest posterior density confidence intervals are annotated for key nodes, posterior probability values are shown for each node, and strain names are shown on the right of the tips. The branch where F313V is believed to have taken place on is annotated in colour (pink and green). Phylogenies were visualised using FigTree.

**Extended Data Fig. 7:**
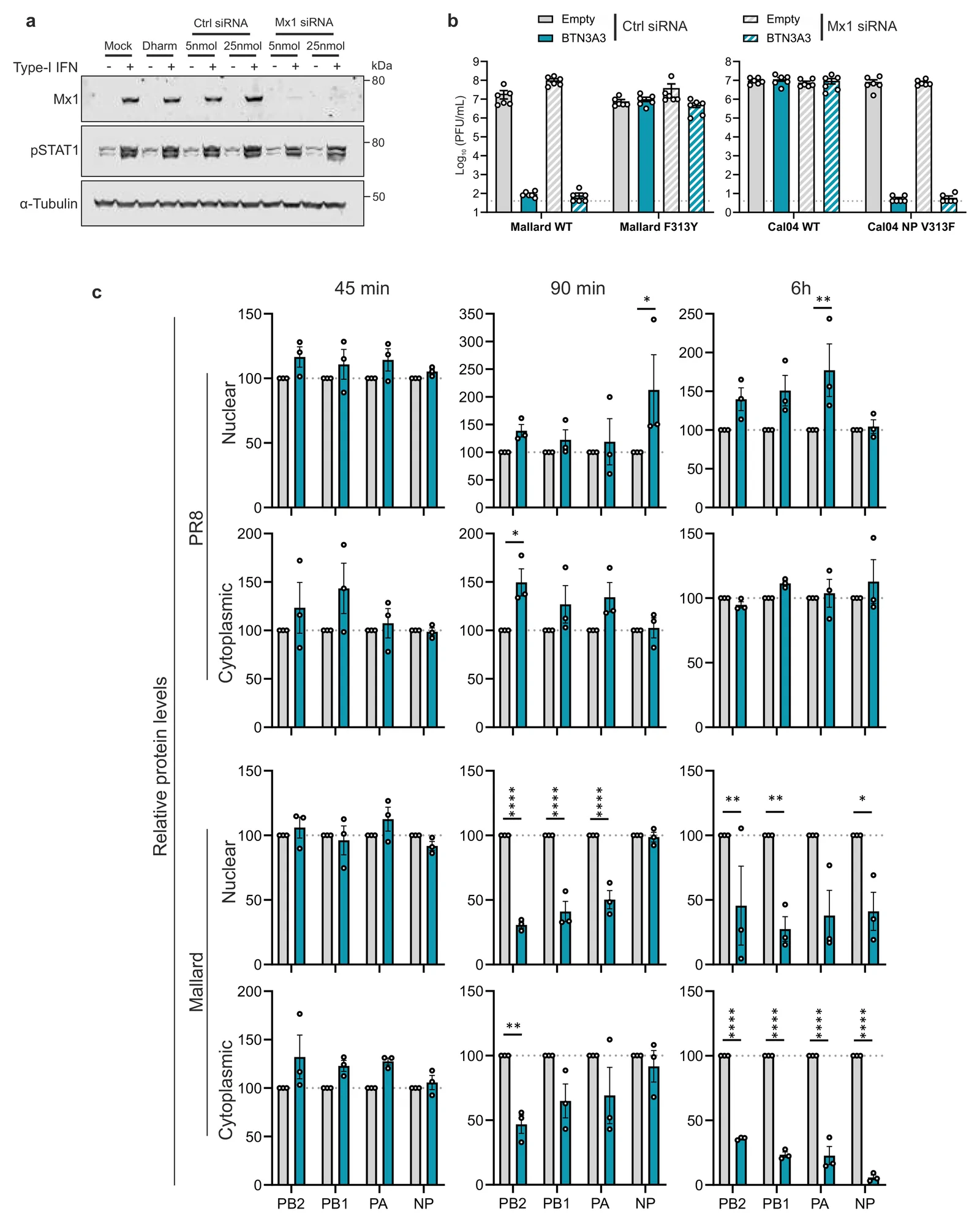
BTN3A3 activity and its relation to Mx1 and vRNP complexes. **a**, Western blotting of cell lysates obtained from A549 cells transfected with the indicated amounts of control or Mx1-targeting pooled siRNAs. Cells were transfected for 48h followed by a 16h type-I IFN treatment. pSTAT1 and α-Tubulin were used as IFN-treatment and loading controls, respectively. For gel source data, see Supplementary Figure 1. **b**, Upon siRNA treatment, A549 Empty and BTN3A3 cells were infected with the indicated viruses at an MOI of 0.001. Supernatants were harvested at 48 hpi and infectious viral titres were measured by plaque assay. Data are mean +/- SEM of 2 technical replicates from 3 independent experiments. **c**, Quantification of cytoplasmic and nuclear levels of vRNP complex proteins at early stages post infection in the presence or absence of BTN3A3. Quantification of 3 independent western blots, one set of which is shown in Fig. 4a. A549-Empty and BTN3A3 overexpressing cells were synchronously infected with PR8 or Mallard at MOI 3. Nuclear/cytoplasm fractionation was performed at 45, 90 mins and 6h post infection. Quantification of vRNP-complex proteins was performed by fluorescence measurements. Cytoplasmic and nuclear viral proteins were normalised to GAPDH and H3, respectively. All values were further normalised to values of A549-Empty cells. Data are mean +/- SEM of 3 independent experiments (each using 2 technical replicates). Statistical significance between groups was measured by a 2-way ANOVA. Comparisons were made between A549-Empty and A549-BTN3A3. NS= non-significant, *p ≤ 0.05, **p ≤ 0.01, ***p ≤ 0.001, ****p ≤0.0001.

NP 313V is specific to classical swine H1N1, which segment 5 entered the human population through reassortment into the 2009 pandemic H1N1. BEAST (Bayesian Evolutionary Analysis Sampling Tree) tip-dating analysis supports a F313V mutation taking place in swine hosts between mid-2002 and the end of 2006 (Extended Data Fig. 6, Supplementary Discussion). Interestingly, pmd09 segment 5, derived from this 313V subclade. We therefore tested if, similarly to PR8, the reversion of NP V313F in pmd09 virus would result in a BTN3A3 sensitive virus. Indeed, unlike its wild-type (WT) counterpart, the mutant Cal04 V313F showed a very pronounced restriction to BTN3A3 (Fig. 3d). Residue 313 had been previously associated with evasion of Mx1 resistance^18,19^. Avian-to-human associated adaptation F313Y, when combined with R100V and/or L283P, exhibits reduced sensitivity to human Mx1^19^. However, we observed that the single F313Y mutation while sufficient to overcome BTN3A3, did not overcome Mx1 restriction (Fig. 3e-f). In addition, loss of function experiments showed that BTN3A3 restriction occurs in an Mx1-independent manner (Extended Data Fig. 7a-b).

We further validated the role of NP residue 313 as a determinant of resistance to BTN3A3 in an *in vivo* experimental model. B6 mice were intranasally transduced with AAV6.2FF vectors expressing either GFP (negative control) or BTN3A3, and subsequently infected with Cal04 WT or V313F mutant reassorted to express PR8 glycoproteins to maximise replication in mouse tissues (6:2 PR8 HA/NA)^31^. AAV achieved efficient expression of our genes of interest in the mouse respiratory tract consistent with abundant GFP and BTN3A3 expression observed in mouse lungs 3 weeks post transduction (Fig. 3g). In challenged mice, Cal04 (6:2 PR8 HA/NA) WT fitness in the lungs was not significantly impaired in BTN3A3-expressing mice compared to controls. Conversely, titres reached by the single mutant Cal04-V313F in BTN3A3-expressing mice were significantly lower than those reached in GFP-expressing mice (Fig. 3h), indicating that BTN3A3 restriction can also occur *in vivo*.

## Mechanisms of BTN3A3 restriction

**Figure 4:**
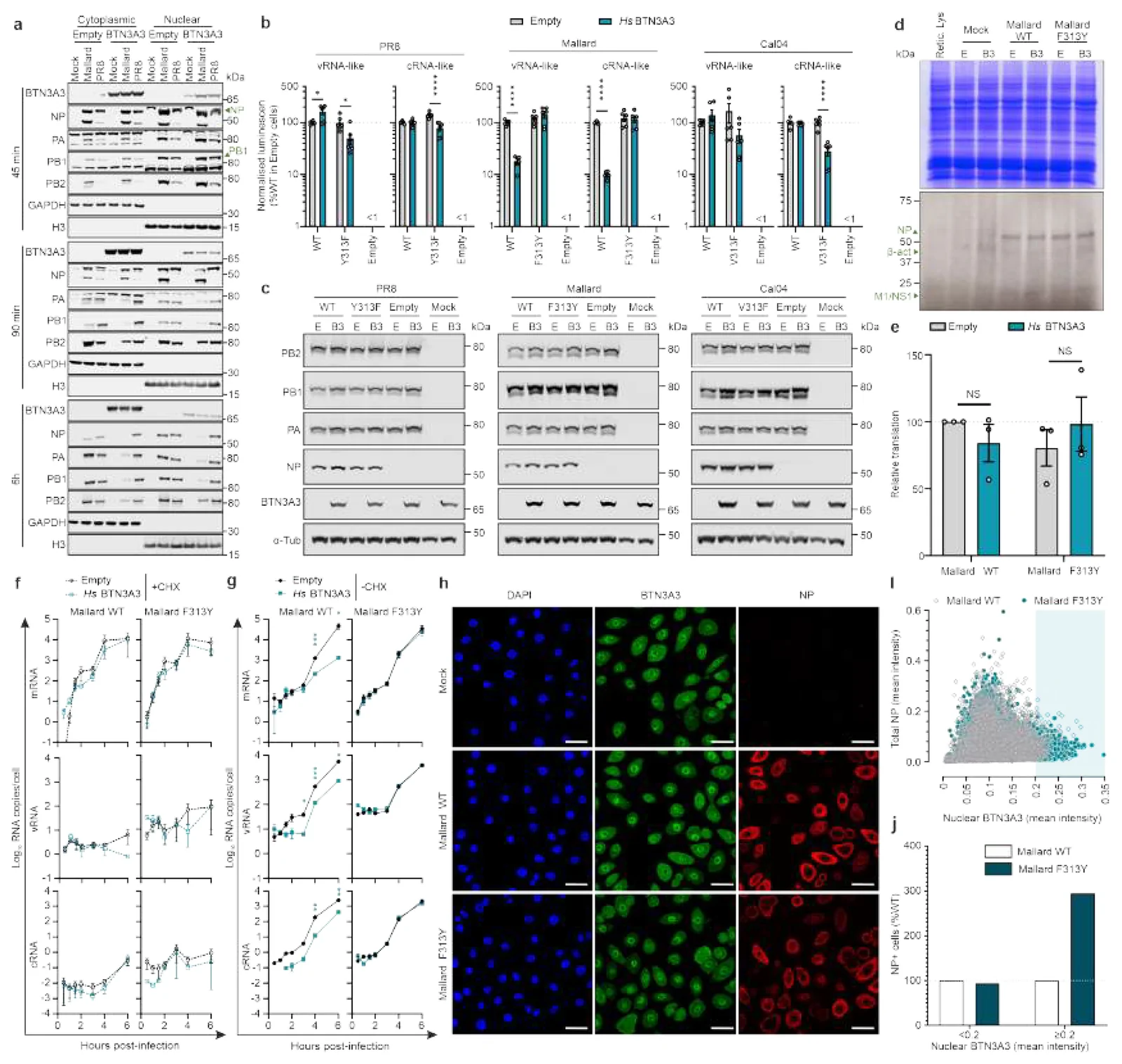
BTN3A3 blocks vRNA transcription. **a**, A549-Empty and A549-BTN3A3 cells were infected with PR8 and Mallard WT (MOI 3). Following nuclear/cytoplasmic fractionation, viral RNP components and cellular markers were detected by western blotting. **b**, Minireplicon assays in 293T-Empty and 293T-BTN3A3 cells of the indicated viruses alongside luciferase-based vRNA- or cRNA-like reporter and transfection control plasmids. 48 h post-transduction luminescence was measured. Values were normalised to WT NP-coding RNPs in 293T-Empty cells. Data are mean +/- SEM of 3 independent experiments (each using 2 technical replicates). **c**, Protein expression of RNP components from 293T-Empty (E) and 293T-BTN3A3 (B3) cells transfected as in (b) were assessed by western blot. **d**, A549-Empty and A549-BTN3A3 cells were pre-treated with CHX and infected at MOI 3. Twelve hpi, total RNA was extracted and equivalent volumes were used in *in vitro* translation assays. Top panel represents Coomassie staining as loading control. Bottom panel exemplifies autoradiography detection. **e**, Densitometry quantification of NP (translated from segment 5 mRNA) from autoradioactive gels. Data are mean +/- SEM of 3 independent experiments and are normalised to Mallard WT in A549-Empty cells. **(f-g)**, A549-Empty and A549-BTN3A3 cells were treated (f) or untreated (g) with 100ug/mL CHX and infected at MOI 3. Viral RNA species were quantified by a 2-step RT-qPCR at indicated time-points. Data are mean +/- SEM of 3 (vRNA and cRNA) or 4 (mRNA) independent experiments. **h**, Representative image of confocal microscopy of hBEC3-KT cells infected with Mallard WT or F313Y at MOI 3. Six hpi, cells were immunostained for NP and BTN3A3 (nuclei in blue). Scale bar = 35µm. **i**, Quantification of total NP and nuclear BTN3A3 intensities for Mallard WT and F313Y. Data constitutes single data points of >3500 cells from 4 independent experiments. **j**, Values from h were stratified based on nuclear BTN3A3 intensity. Data represents relative abundance of total infected cells in each nuclear BTN3A3 intensity ranges. Values were normalised to Mallard WT. Statistical analyses in this figure are the result of multiple t-tests between Empty and BTN3A3 conditions and were performed using the Holm-Šídák method. Annotations are defined as NS=Non-significant, *p≤0.05, **p≤0.01, ***p≤0.001, ****p≤0.0001. Samples in (a) and (c) were derived from the same experiment and gels were processed in parallel. For gel source data, see Supplementary Figure 1.

NP is an essential protein in the IAV life cycle with the primary function of encapsidating the viral genome to ensure effective RNA transcription, replication and packaging^32^. As well as being a structural RNA-binding protein, NP is also a key adapter between virus and host cell processes as it is essential for the nucleo/cytoplasmic trafficking of viral ribonucleoproteins (vRNPs)^32–34^. Hence, to test whether BTN3A3 inhibits vRNP nuclear import we performed synchronised infections of A549-Empty or A549-BTN3A3 cells followed by nuclear/cytoplasmic fractionation at early time points post infection. At 45 minutes post-infection, levels of Mallard vRNP-containing proteins in both cytoplasmic and nuclear fractions were equal between A549-Empty and BTN3A3-expressing cells, suggesting equally efficient virus entry and vRNP nuclear import (Fig. 4a top panel, and related quantification presented in Extended Data Fig. 7c). At 90 minutes post-infection, by which point initial transcription and translation of viral genes had resulted in an increase of viral protein, a difference in viral protein levels was seen between the two cell lines in both cytoplasmic and nuclear fractions (Fig. 4a middle panel, Extended Data Fig. 7c). This difference was further amplified at 6 hpi (Fig. 4a bottom panel, Extended Data Fig. S9). Hence, these data suggested that the initial stages of the virus life cycle including binding, entry and vRNP nuclear import were largely unaffected by BTN3A3 overexpression while subsequent steps could be affected.

**Extended Data Fig. 8:**
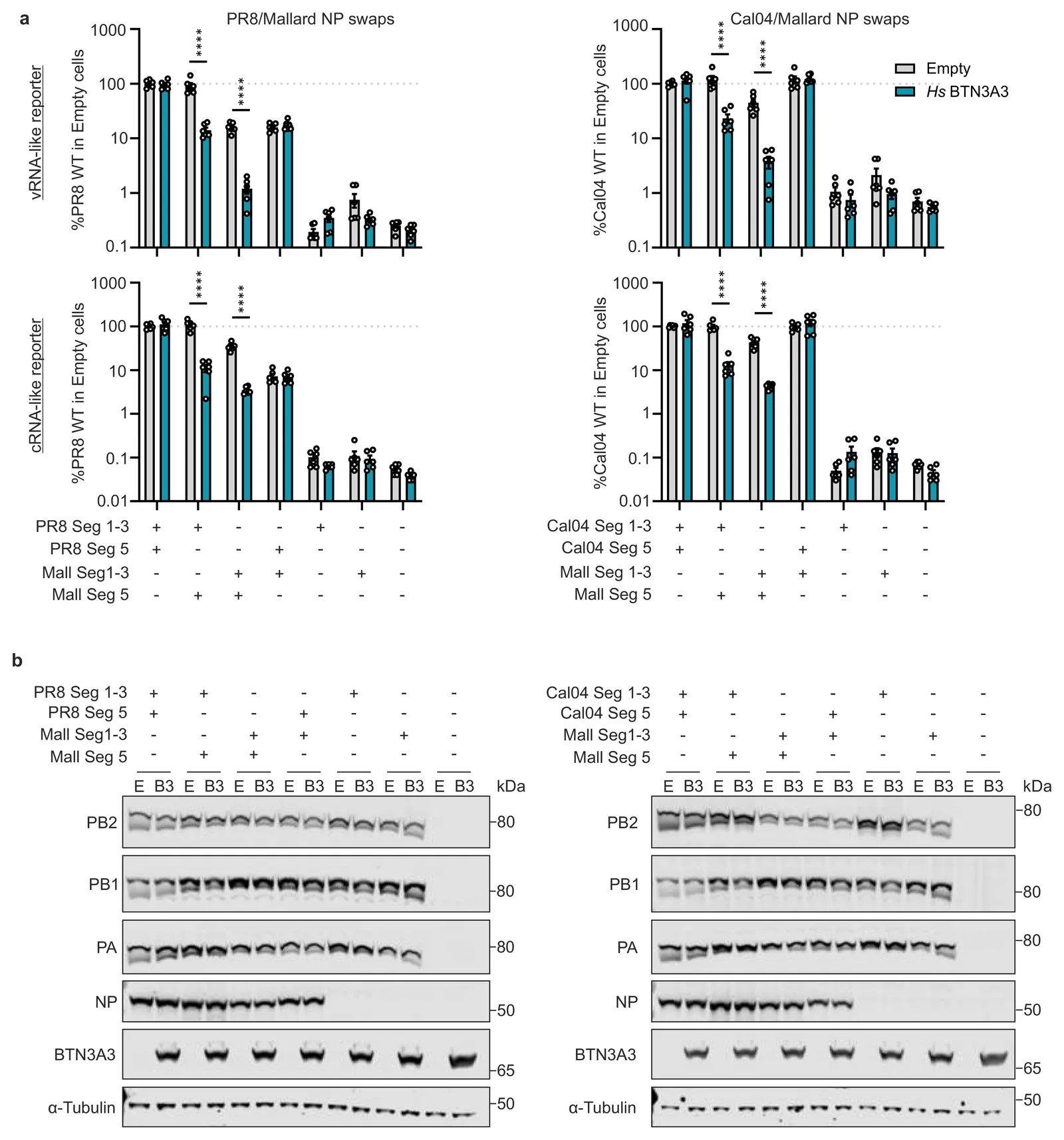
Minireplicon assays with avian/mammalian NP reassortant RNP complexes. **a**, 293T-Empty and 293T-BTN3A3 cells were transfected with pcDNA plasmids encoding for PB2, PB1, PA and NP of the indicated viruses alongside firefly luciferase-coding vRNA- or cRNA-like reporter plasmids. A transfection control plasmid expressing *Renilla* firefly was also added. Forty-eight hours post-transection cells were lysed and firefly and *Renilla* luciferase activities were measured. Values were normalised to PR8 or Cal04 WT replicons with respective NPs transfected in 293T-Empty cells. Data are mean +/- SEM of 3 independent experiments (each using 2 technical replicates). Statistical differences between Empty and *Hs*BTN3A3 overexpressing cells were calculated using multiple t-tests and corrected for multiple comparisons using the Holm-Šídák method. NS= non-significant, *p≤0.05, **p≤0.01, ***p≤0.001, ****p≤0.0001. **b**. Expression levels of PB2, PB1, PA and NP transfected in 293T-Empty (E) or 293T-BTN3A3 (B3) cells were assessed by western blot. α-Tubulin was used as loading control. For gel source data, see Supplementary Figure 1.

To investigate this further, we measured RNP-driven viral transcription/replication and translation using RNP reconstitution minireplicon reporter assays. Using either vRNA- or cRNA-like reporter constructs, the transcription/replication activities of NP 313F-coding RNPs were significantly reduced in BTN3A3-overexpressing cells (Fig. 4b-c; Extended Data Fig. 8). In principle, this could be due to effects on viral transcription, replication, translation or protein stability.

To examine the effects of BTN3A3 in viral primary transcription, A549-Empty and A549-BTN3A3 cells were pre-treated with cycloheximide (CHX; a translation elongation inhibitor) and subsequently infected with Mallard WT or the F313Y mutant. Viral mRNA was left to accumulate for 12h, RNA was extracted and protein synthesis from viral mRNA was measured using a rabbit reticulocyte lysate system. For both Mallard WT and Mallard F313Y, autoradiography analysis revealed equal levels of major viral proteins between A549-Empty and A549-BTN3A3 cells (Fig. 4d-e), suggesting that viral primary transcription was not inhibited by BTN3A3.

We next tested if RNP replication activity was impaired by BTN3A3 using qPCR assays to measure viral mRNA, vRNA and cRNA species at early time points post-infection in the presence or absence of CHX. The ablation of protein synthesis by CHX results in the abrogation/inhibition of IAV genome replication activity which is dependent on newly synthesised NP monomers^35^. Therefore, new vRNA and cRNA synthesis was fully inhibited by CHX (Fig. 4f, middle and bottom panels). Consistent with observations in Fig. 4d-e, in the presence of CHX, Mallard WT mRNA levels were not affected by BTN3A3 (Fig.4f, top panels). In CHX-untreated cells, Mallard WT mRNA levels were insensitive to BTN3A3 in the first 3 hpi, but thereafter were reduced, by nearly 100-fold at 6 hpi. Potentially explaining this late difference in viral mRNA synthesis, BTN3A3 overexpression both reduced and delayed vRNA and cRNA accumulation in Mallard WT-infected cells. In contrast, the BTN3A3-resistant mutant Mallard F313Y showed no differences in the synthesis of any viral RNA species between infected A549-Empty and A549-BTN3A3 cells (Fig. 4f-g, right halves). Collectively these data suggest that BTN3A3 restricts sensitive IAV mainly at the level of viral genome replication.

**Extended Data Figure 9:**
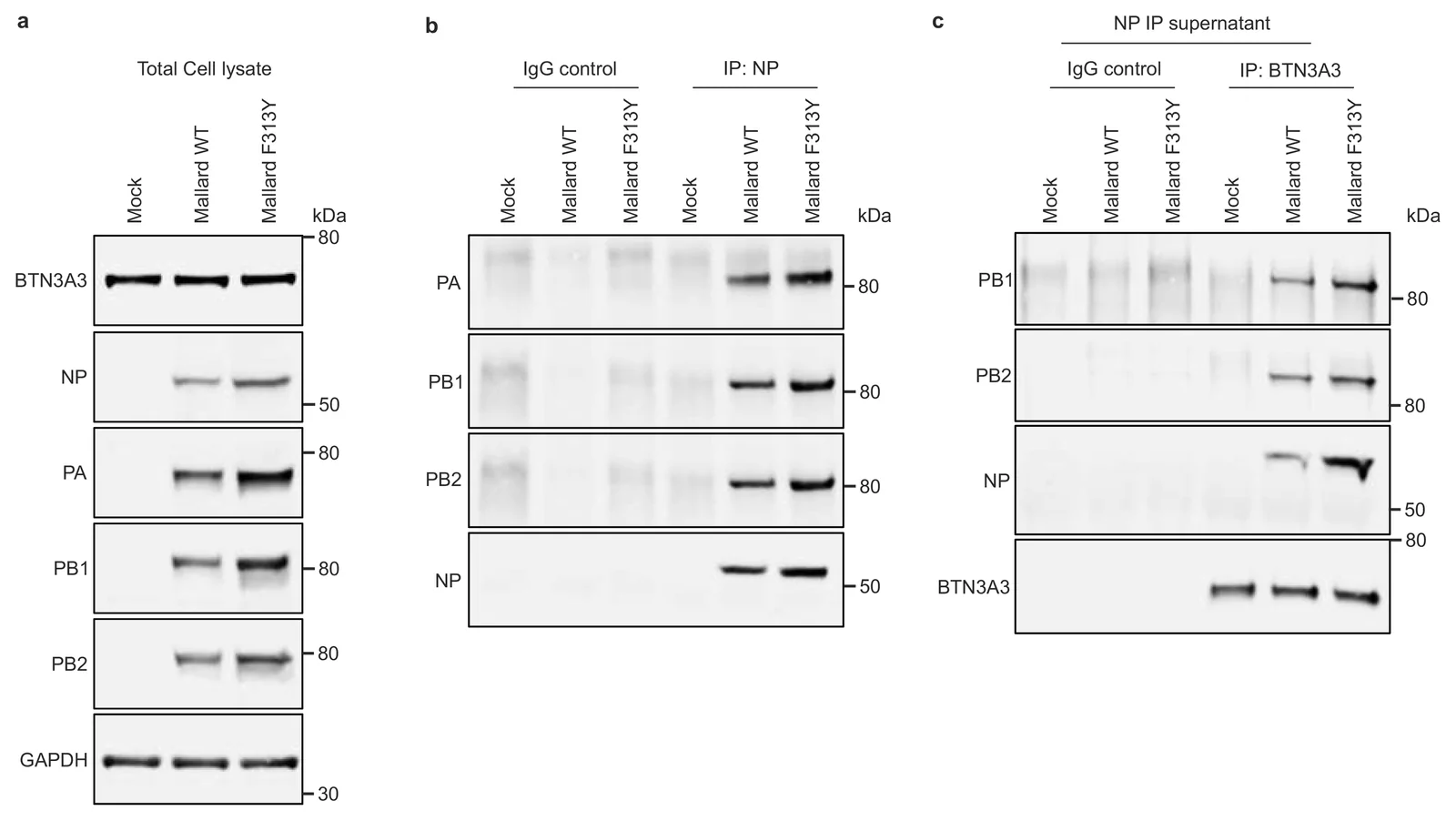
Interactions between NP and BTN3A3. **a**, Western blotting of hBEC3-KT cells infected with IAV Mallard WT or Mallard NP F313Y for 6 h. Expression of NP, PA, PB1, PB2 and GAPDH (loading control) from total cell lysates is shown. **b**, Total cell lysates of infected cells were used to perform NP immunoprecipitation followed by the detection of the remaining RNP complex-forming proteins. **c**, Supernatants of the NP immunoprecipitates was used to perform an additional immunoprecipitation using an anti-BTN3A3 antibody followed by the detection of PB2, PB1 and NP. For gel source data, see Supplementary Figure 1.

**Extended Data Figure 10:**
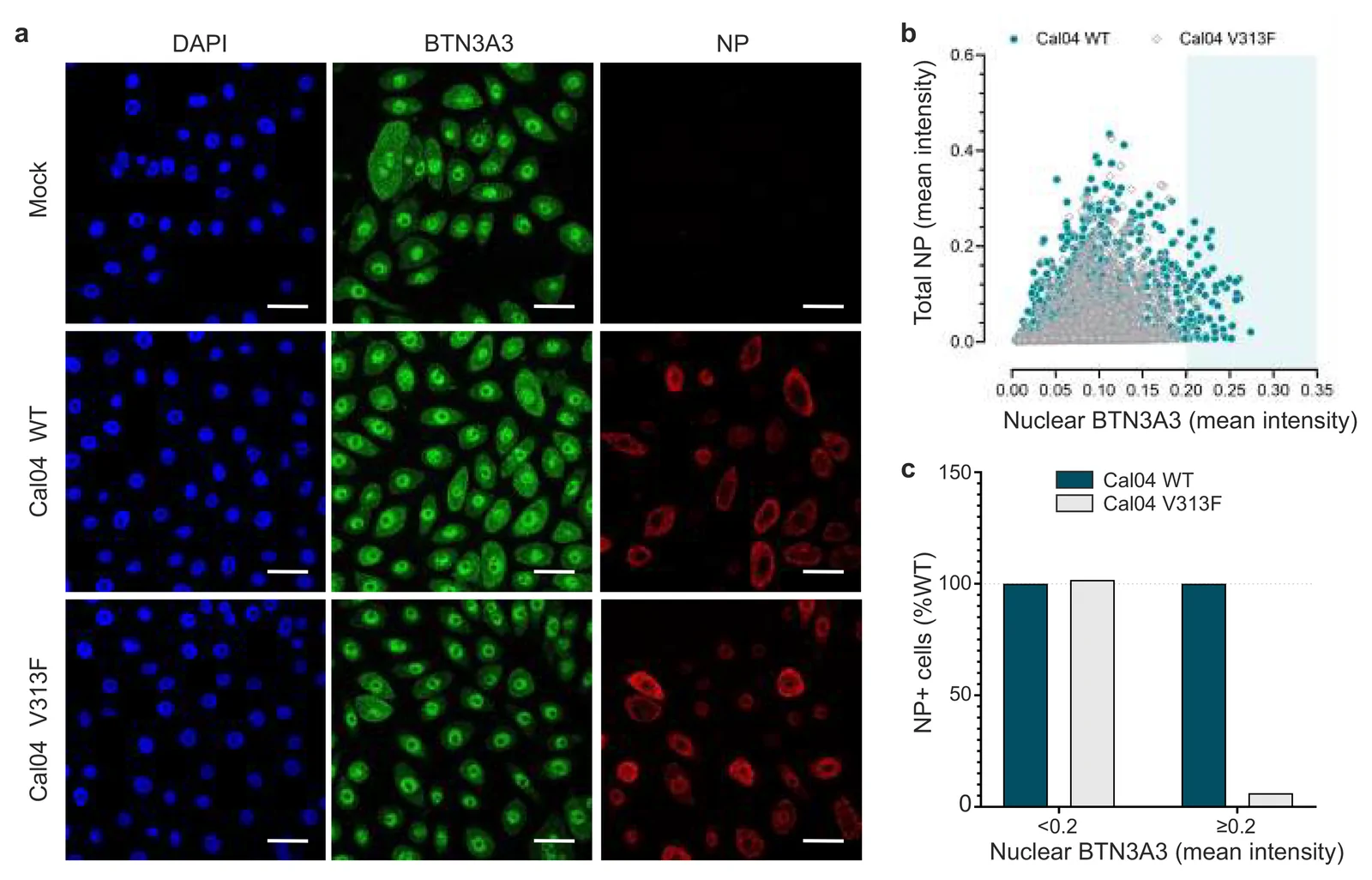
Correlation between NP signal and nuclear BTN3A3. **a**, Representative images of confocal microscopy of hBEC3-KT cells infected with Cal04WT or Cal04 NP V313F at MOI 3. Six hours post infection, cells were immunostained with NP (red) and BTN3A3 (green). DAPI staining (blue) was used as a nuclear marker. Scale bar= 35µm. **b**, Images from >3500 cells from four independent experiments performed as in (**a**) were used to quantify total NP and nuclear BTN3A3 for Cal04 WT and Cal04 NP V313F. **c**, Values from b were stratified based on nuclear BTN3A3 intensity (<0.2 or ≥0.2). Data represents relative abundance of total infected cells present in each of the two nuclear BTN3A3 intensities ranges, taking values obtained with Cal04 WT as 100%.

IAV RNA synthesis takes place in the nucleus, and despite BTN3A3 having been described as a transmembrane protein, localisation of constitutively expressed BTN3A3 in hBEC3-KT cells was predominantly nuclear (Fig. 4h). Interestingly, in these cells, we also found components of the RNP complex, of both avian and mammalian IAV, could also be co-immunoprecipitated with BTN3A3 (Extended Data Fig. 9). Image analysis of infected hBEC3-KT cells showed that, when cells express high levels of nuclear BTN3A3, viral protein synthesis (measured by NP accumulation) of Mallard WT was weaker (Fig. 4i-j). Additionally, NP accumulation from a BTN3A3-resistant mutant was less sensitive to high levels of nuclear BTN3A3. Similar observations were made for Cal04 WT (BTN3A3 resistant) and the BTN3A3-sensitive mutant Cal04 NP V313F (Extended Data Fig. 10).

## Virus genotype of zoonotic avian IAV

**Figure 5:**
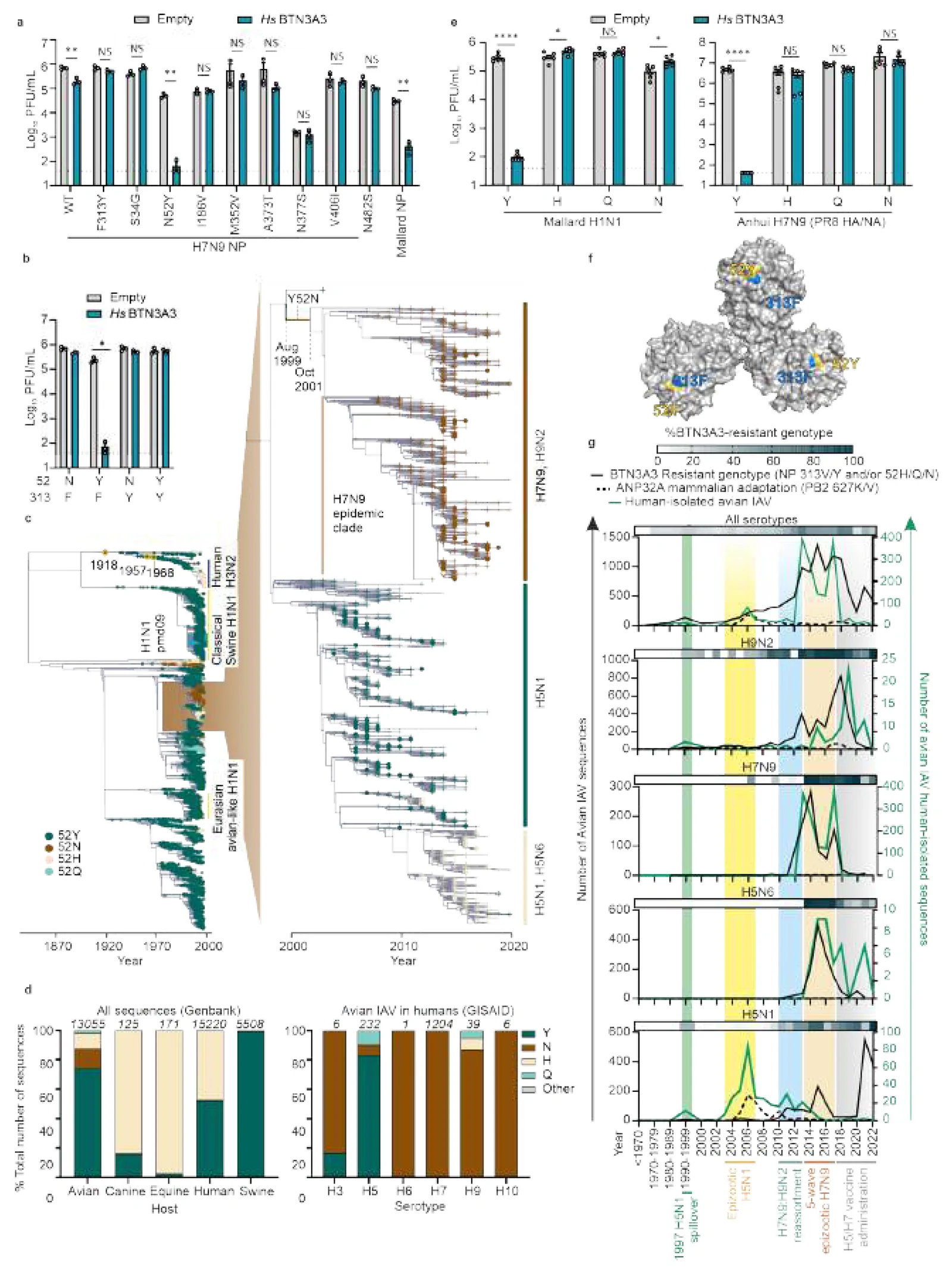
Evasion of BTN3A3 by zoonotic avian IAV. **a-b**, Replication of H7N9 (6:2 PR8 HA/NA) reassortant viruses with the indicated NP mutations in A549-Empty or A549-BTN3A3 cells. Cells were infected at an MOI of 0.001 for 48h and viruses titrated by plaque assay. Data are mean +/- SEM from 3 independent experiments (each using 2 technical replicates). Multiple t-tests were performed using the Holm-Šídák method. **c**, Tip-dated maximum likelihood phylogenies of the filtered IAV NP coding sequence dataset (left) and of all sequences clustering within the highlighted avian IAV clade (right). Tip shapes are coloured by site 52 residues (only residues present in more than 1% of the sequences are shown in the legend). Right: human isolates are annotated with circles and non-human isolates with transparent crosses to highlight human transmission. The branch where the Y52N change took place is annotated on the tree. Serotypes present in more than 10% of the sequences in each subclade are shown on the right. **d**, Identity of amino acid residue in position 52 of all NP proteins available in the NCBI flu dataset used in this study (see Methods) (left) or in those collected from spillovers of avian IAV in humans (GISAID) (right). Numbers over the bars indicate number of sequences. **e**. Infectious virus titres obtained in A549-Empty and BTN3A3 expressing cells with NP 52 mutants of either Mallard or H7N9 6:2 PR8. Cells were infected at an MOI of 0.001 for 48h and viruses titrated by plaque assay. Data are mean +/- SEM from 3 independent experiments (each using 2 technical replicates; statistical analysis performed as in a-b). **f**, Surface structure of trimeric NP (residues 52 and 313 are highlighted). **g**, Chronological association between the accumulation of the BTN3A3-resistant genotype and PB2 627K/V in IAV circulating in avian species or isolated from human patients. Statistical annotations in this figure are defined as NS = Non-significant, *p≤0.05, **p≤0.01, ***p≤0.001, ****p≤0.0001.

We next analysed whether BTN3A3 evasion could be correlated with the zoonotic potential of avian IAV. We focused on avian H7N9 given the high numbers of spillover events caused by these viruses. The NP of H7N9 contains a 313F residue and so theoretically should be restricted by BTN3A3. However, the high number of human infections caused by H7N9, led us to hypothesise that they overcome BTN3A3 restriction through an alternative mechanism. We tested a 6:2 H7N9 reassortant (containing the glycoproteins from PR8 and the internal segments from H7N9) in A549-Empty and A549-BTN3A3 cells. The reassortant H7N9 was poorly restricted by BTN3A3, despite possessing a 313F-coding NP, whereas a 5:3 H7N9 reassortant containing Mallard NP was restricted ~70 fold on average (Fig. 5a). Comparison of H7N9 and Mallard NP sequences highlighted differences at 8 amino acid residues (positions 34, 52, 186, 352, 373, 377, 406 and 482). To determine if any of these amino acid substitutions enabled H7N9 viruses to evade BTN3A3 restriction, we constructed and tested single amino acid mutants, and identified that the NP substitution N52Y rendered H7N9 sensitive to BTN3A3 (Fig. 5a). The effect of 52N was only seen in the presence of 313F; the Y52N mutation was not sufficient to confer BTN3A3-sensitivity in the presence of 313Y (Fig. 5b).

52N-coding NP sequences occur in multiple independent avian IAV sub-lineages. Most importantly, using time-calibrated phylogenetic analysis, we identified an initial switch of NP 52Y to 52N that we estimated occurred between August 1999 and October 2001 and that over time became dominant in the avian H9N2 and the H7N9 lineages from which all human H7N9 epidemic virus NPs have originated (Fig. 5c).

To further explore the importance of NP residue at position 52, we analysed its polymorphism across all strains of IAV. Avian IAV predominantly encode NP 52Y with 25% of the isolates coding for 52N or 52H (Fig. 5d, left). Swine IAV almost universally contain NP 52Y while equine and canine NP sequences present a majority of 52H. Human-derived IAV possess either 52Y or 52H in broadly equal parts (Fig. 5d, left). The substantial prevalence of 52Y in human IAV can be explained by the co-occurrence of NP 313Y which provides resistance to BTN3A3 and is dominant over 52Y (Fig. 5b). Analysis of all subtypes of avian IAV NP sequences obtained from humans (representing spillover events) showed not only the majority 52N in H3, H6, H7, H9 and H10 subtypes, but also less frequent residues 52Q, 52H and 52Y sidechains (Fig. 5d, right, and Supplementary Table 2), with the latter almost universally in the H5 subtype. We show that mutants engineered with 52Q or 52H in either the Mallard or H7N9 backgrounds result in BTN3A3 evasion as efficiently as those possessing 52N (Fig. 5e). Interestingly, despite being separated in the primary sequence of NP, positions 52 and 313 are closely juxtaposed within the NP head domain (Fig. 5f). Moreover, both residues are located on the surface of the NP trimer, indicating that they would be accessible to interactions with other viral and host factors when NP oligomerises to form cRNP and/or vRNP.

**Extended Data Fig. 11:**
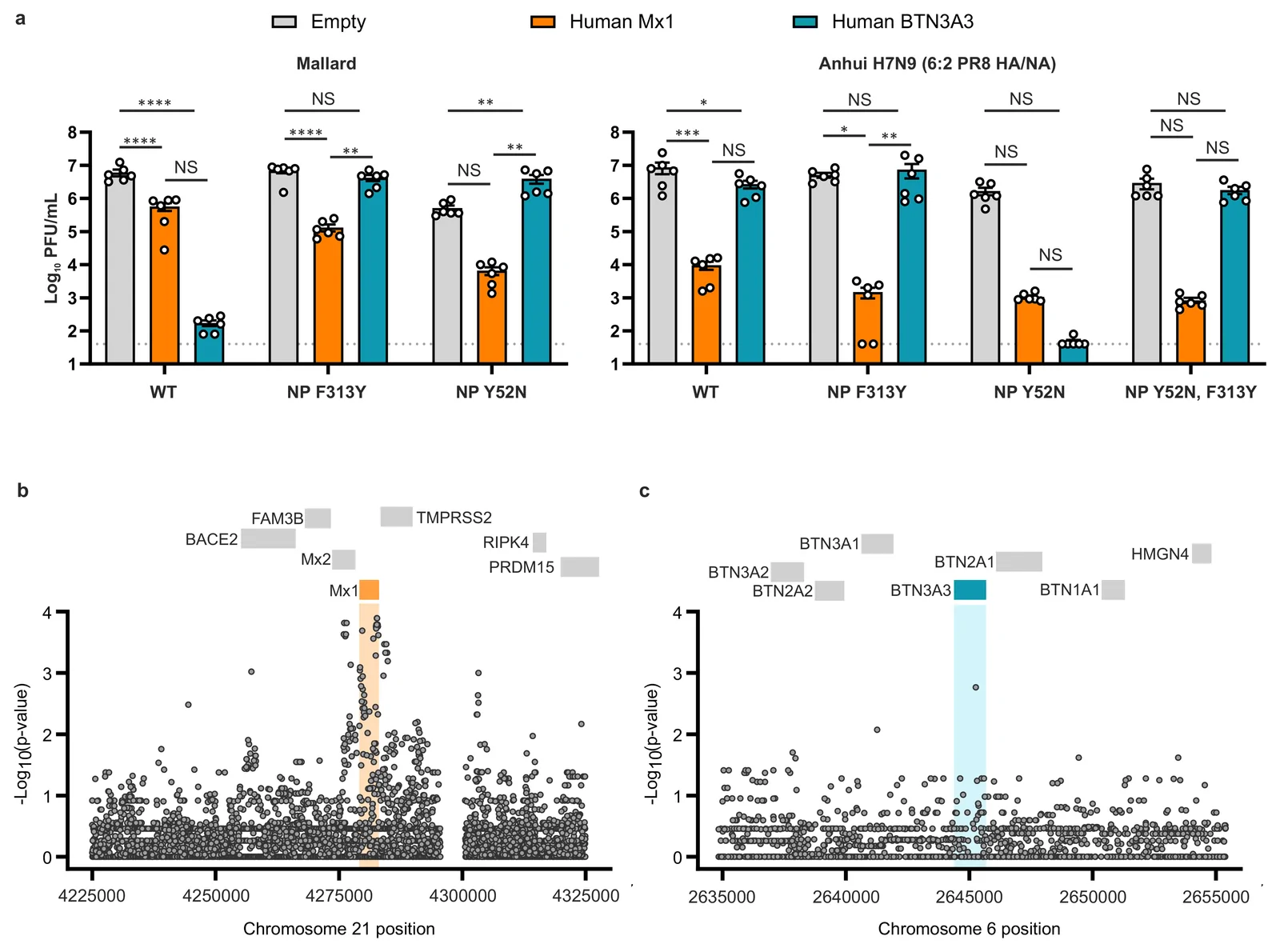
Distinct requirements of NP residues for BTN3A3 and Mx1 evasion, and GWAS analysis of H7N9 patients. **a**, A549 cells overexpressing human Mx1 or BTN3A3 were infected with the mentioned viruses at a MOI of 0.001. Supernatants were harvested at 48 hpi and infectious viral titres were measured by plaque assay. Data are mean +/- SEM of 3 independent experiments (each using 2 technical replicates). Statistical significance between groups was measured by a 2-way ANOVA. NS-non-significant, * p ≤ 0.05, ** p ≤ 0.01, *** p ≤ 0.001, **** p ≤ 0.0001. (**b-c**) GWAS analysis of H7N9-infected patients. Manhattan plots of MX1 (**b**) and BTN3A3 (**c**) genomic regions. Gene-level p-values were acquired using RACER in R from data obtained from Chen, et al., *Science* **373**, 918-922, doi:10.1126/science.abg5953 (2021).

It is important to note that NP residue 52 has been also implicated in the evasion of Mx1 resistance^19,20^. However, by overexpression of BTN3A3 or Mx1, we determined that IAV sensitivity/resistance to these restriction factors showed differential dependence on NP residues 52 and 313 (Extended Data Fig. 11a). The evasion of BTN3A3 restriction conferred by residue 52, is dependent on residue 313, which is not the case for evasion of Mx1 restriction. Interestingly, a correlation in human patients between disease severity induced by H7N9 and certain human MX1 alleles has also been described in GWAS studies^36^. Incidentally, BTN3A3 is very conserved in humans (Supplementary Table 3) and we found no specific association with severe disease and BTN3A3 alleles using the same GWAS analyses (Extended Data Fig. 11b-c).

**Extended Data Fig. 12:**
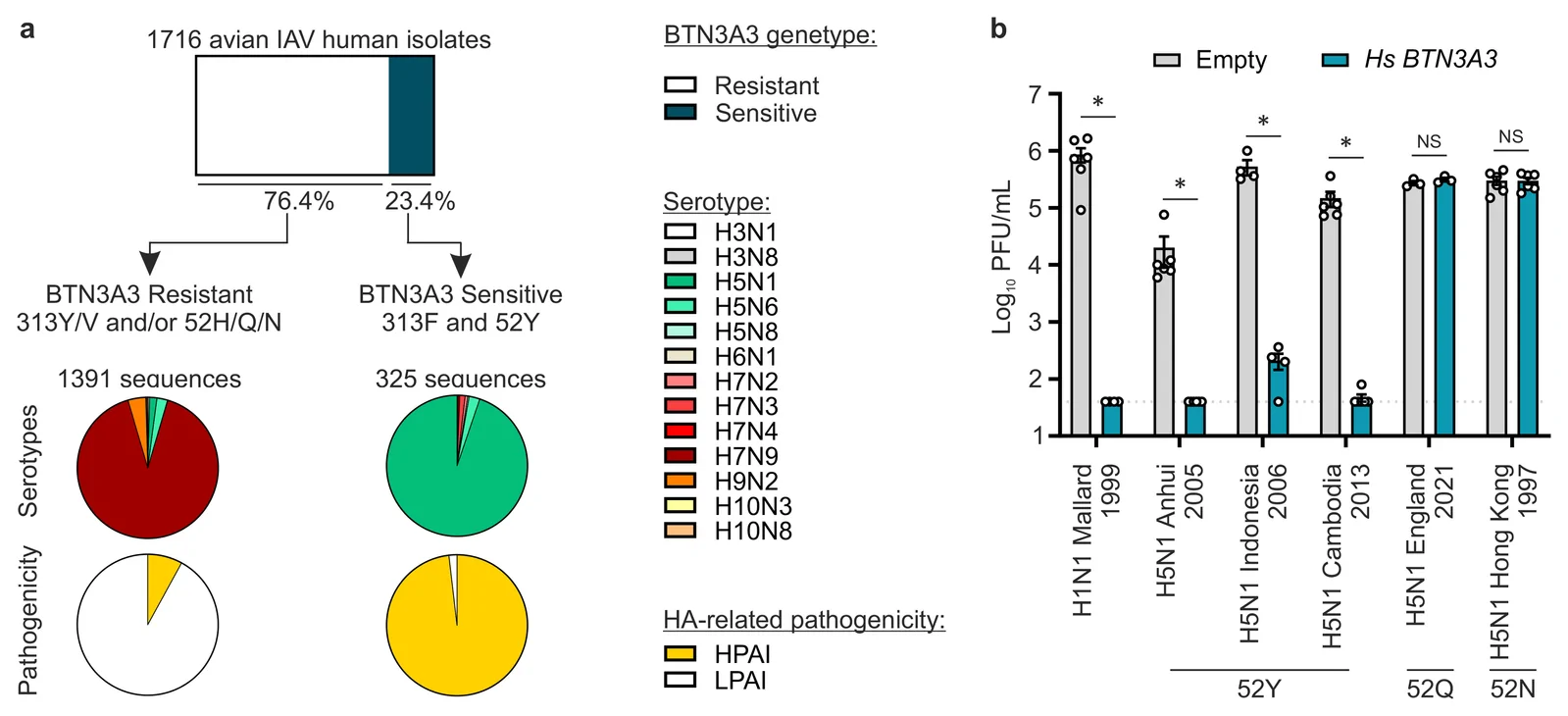
BTN3A3 restriction by highly pathogenic H5N1. **a**, Schematic representation of numbers and characteristics of avian IAV NP sequences identified in human spillover events. Total number of sequences was divided into BTN3A3-resistant or sensitive genotypes based on amino acid residues 313 and 52. NP sequences were matched with their respective HA sequences and highly pathogenic avian influenza (HPAI) viruses were separated from the low pathogenic (LPAI) based on the presence of a polybasic cleavage site (only present in HPAI isolates). **b**, Replication of 7:1 reassortants of PR8 encoding segment 5 from Mallard or H5N1 HPAI viruses in A549-Empty or A549-BTN3A3 expressing cells. Cells were infected at an MOI of 0.001 for 48h and titres were measured by plaque assay. Data are mean +/- SEM of 3 independent experiments (each using 2 technical replicates) with the exception of 52Q which was instead a single technical replicate from 3 independent experiments. Statistical differences between values obtained in empty and BTN3A3 expressing cells were calculated using multiple t-tests and corrected for multiple comparisons using the Holm-Šídák method. NS= non-significant, *p≤0.05, **p≤0.01, ***p≤0.001, ****p≤0.0001.

In summary, a BTN3A3-sensitive viral genotype can be defined as possessing NP coding 313F and 52Y while BTN3A3-resistant viruses have NP 313Y/V and/or 52N/H/Q. Of the ~1700 avian IAV sequences detected in human patients, 77% have a BTN3A3-resistant genotype, while the remaining 23% are BTN3A3-senstive. The latter belong virtually all to highly pathogenic avian influenza H5N1 (308 of 309 sequences; Extended Data Fig. 12a). Using a 7:1 PR8-based reassortants, we experimentally confirmed that viruses harbouring H5N1 NP sequences with a predicted BTN3A3-sensitive genotype were indeed restricted in A549-BTN3A3 cells (Extended data Fig. 12b). Importantly, 99.5% of BTN3A3-resistant viruses isolated from human patients originated from low pathogenic avian influenza (LPAI) lineages.

## Frequency of BTN3A3-resistant avian IAV

We next examined how the frequency of the BTN3A3-resistant genotype changed over time in avian IAV circulating in birds, including those involved in major epizootic events. Analysis of the frequency of BTN3A3-resistant genotype among all IAV isolates (~29,000 sequences; Supplementary Table 4) from birds showed a peak between 2012 and 2019, fluctuating from 49.5 to 62%. This correlates with peaks of avian-to-human IAV zoonotic cases within the same time period (Fig. 5g, first panel).

Avian H9N2 viruses represent the serotype with the highest percentage of BTN3A3-resistant genotype, having reached a maximum of 97% in 2018, along with a high number of detected sequences (Fig. 5g, second panel). The H9N2 G57 genotype is highly promiscuous and is regarded as a main donor of viral internal genes that resulted in viruses spilling over into humans^37^. Through reassortment events, prior to 2013, H9N2 gifted its internal gene segments to the newly emerging H7N9 strain. Consequently, while H9N2 human isolates have been detected sporadically since 1999, H7N9 was first detected in humans in 2013, resulting in a 5-wave epizootic event lasting until 2018 (Fig. 5g, third panel). Moreover, of all avian-isolated H7N9 sequences deposited in GISAID before 2013, only 14% (7 out of 50) display a BTN3A3-resistant genotype (conferred by NP 52H). In contrast, from 2013 onwards, 93.5% of H7N9 (n=849) show a BTN3A3-resistant genotype that is instead conferred by the H9N2 G57-descendant NP 52N.

In addition to H7N9, another example of a G57-segment containing virus is the HPAI H5N6 with its first human infection being detected in 2014^38^, reaching a peak of human infections in 2015-16. Like the H7N9 epizootic event, the chronology of acquisition of BTN3A3-resistance correlates with H5N6 avian-to-human spillovers (Fig. 5g).

For comparative purposes, we also analysed PB2 amino acid 627, as it is well reported that viruses harbouring 627K (or 627V) have a replicative advantage in human cells as they are better supported by human ANP32A than the avian counterpart 627E^15^. In the analysed dataset, ~63% of all avian IAV isolated in human patients possess a PB2 627K/V compared to a frequency of ~3% in viruses circulating in birds. Specifically in the 2013-2018 H7N9 epizootic waves, 74% of viruses isolated from human patients possessed a PB2 627K/V. However, very few avian H9N2, H7N9 and H5N6 circulating in birds harboured the “mammalian”-adapted PB2 627K/V either before, during or after zoonotic events. These data suggest that BTN3A3 resistance is important for spillover whilst the acquisition of 627K/V is selected in humans infected with avian IAV.

As described above, the HPAI H5N1 spillover events do not show overall an association with a BTN3A3-resistant genotype. It is however interesting to note that 10 of 10 sequences publicly available from the first outbreak caused by H5N1 in 1997 in Hong Kong^39^ display a BTN3A3-resistant genotype. In addition, although the number of sequences available from H5N1 circulating in birds from the same period and location are relatively low (n=8), they also all possess a BTN3A3-resistant genotype. Another interesting observation is associated with the waterfowl-origin A/Goose/Guangdong/1/96 (GsGd) lineage of HPAI H5N1 viruses which re-emerged in China in 2003^40^. Unlike H9N2, H7N9 and H5N6, avian isolates of HPAI H5N1 showed a high frequency of the PB2 627K residue, in approximately the same period when an increased number of sequences is available of zoonotic cases caused by these viruses (Fig. 5g, lower panel).

## Discussion

Our study reveals that human BTN3A3 is a powerful barrier for the replication of avian, but not human IAV. Through data obtained *in vitro* in cell culture assays and *in vivo* in experimental animal models, in addition to comprehensive evolutionary analyses, we showed that evasion of human BTN3A3 is one of the risk factors for the zoonotic potential of IAV. To date, avian subtypes H5, H7, H9, and less often H3, H6 and H10, have spilled over and caused human infections^7 41^.

Butyrophilins were discovered originally as proteins involved in lactation and milk production, but they are also known to have immunomodulatory functions^2,42–44^. This group of proteins have not been generally associated with antiviral properties, although one of two studies using ISG libraries against Ebola virus replicons identified BTN3A3 as a restriction factor for this virus^45,46^. The anti-avian IAV properties of BTN3A3 arose in primates. Hence, humans are the only species maintaining endemic infections with influenza viruses that restrict avian IAV replication through this effector. Neither avian species nor swine impose a BTN3A3-related selective pressure on the NP 313 residue.

We showed that BTN3A3 interferes mostly with avian IAV viral RNA replication, at the early stages of the virus life cycle after the entry of the viral ribonucleoprotein complex into the nucleus and primary vRNA transcription.

There are several independent lines of evidence pointing to evasion of BTN3A3 as a key risk factor for the zoonotic potential of IAV. First, all the human endemic IAV viruses, including those that emerged in 1918, are resistant to BTN3A3. Second, the swine H1N1 2009 pandemic virus originated from a BTN3A3 resistant NP 313V clade, that we estimate appeared in pigs between 2002 and 2006, ahead of the start of the 2009 pandemic (Extended Data Fig. 6). Third, evasion of BTN3A3 restriction is a conserved trait of the majority of avian viruses that have successfully spilled over into humans, due to a mutation from a BTN3A3-sensitive 52Y residue to a BTN3A3-resistant N, Q or H residue (or very rarely in H3N8 through the F313V mutation) and further hypothesise that residue 52 masks the effect of 313F^3^. Finally, we showed that avian IAV circulating in birds showed an increased frequency of the BTN3A3-resistant genotype in 2011-2012, ahead of the initial H7N9 outbreak in humans. Indeed, it is only around 2013, when the first human H7N9 cases were identified, that the NP 52N residue became dominant in this lineage.

There are multiple barriers preventing zoonotic cross-species transmission of animal IAV, and BTN3A3 has to be considered one of many rather than the sole determinant of the zoonotic potential of IAV. Mx1 is another restriction factor for avian IAVs. The NP residues 313 and 52 which are critical BTN3A3 evasion/resistance have been associated also with Mx1 resistance^18,19^. We presented *in vitro* data suggesting that, despite affecting similar stages of the virus life cycle and through common amino acid residues, BTN3A3 acts independently from Mx1. Moreover, our *in vivo* experiments were carried out in regular C57BL/6 which, like many other laboratory mouse strains, possess a non-functional Mx1^47,48^, further reinforcing the point that BTN3A3 acts in an Mx1-independent manner. It is also interesting to note a recent study suggesting that IAV NP regulates mitophagy and the Y313F mutation attenuates this process^49^. Hence, at least two independent human restriction factors target a conserved surface of NP, suggesting that this constrained region is a key determinant of the zoonotic potential of avian IAV.

The exception to the rule of BTN3A3 representing a key barrier to avian IAV spillover into humans is represented by highly pathogenic H5N1. HPAI viruses are defined by the presence of a polybasic cleavage sites in their HA protein which may allow the virus a wider cellular tropism, potentially providing the ability to infect BTN3A3 low-expressing cells, or a more efficient replication fitness leading to a higher chance of a successful infection^50^ and therefore overriding the antiviral effects of BTN3A3. Interestingly, only ~ 30% of H5N1 isolated from humans possess the adaptive PB2 627K mutation, as opposed to more than 70% of all the other avian viruses that successfully spilled over into humans. Hence, HPAI H5N1 viruses appear to be able to infect humans in the absence of either a BTN3A3-resistant genotype or PB2 human adapting mutations. These observations suggest that this virus may possess distinctive features that allow it to infect humans more effectively than other avian viruses.

In recent years, the GsGd lineage of HPAI H5 virus that originally emerged in Asia, has caused an increasing number of outbreaks in wild birds, poultry and wild mammals across multiple continents^51^. This lineage displays a high frequency of the BTN3A3-resistant genotype. Of note, is a particularly concerning recent outbreak of H5N1 in a farm housing ~50,000 mink in Spain^52^. Viruses isolated from this outbreak displayed both an ANP32A-linked PB2 T271A mutation, associated with enhanced polymerase activity in mammalian cells^53^, and a NP 52N mutation conferring a BTN3A3-resistant genotype which was acquired in an avian host prior to emergence of the H5N1 virus in mink^52^. Transmission of these viruses within farmed animal populations provide them with the opportunity to further adapt to the mammalian host, in addition to provide further opportunities for onwards transmission to humans. Hence, H5N1 viruses with a BTN3A3-resistant genotype may be better posed in the future to adapt to humans.

Global efforts during the SARS-CoV-2 pandemic and over the years for IAV infections have shown that surveillance based on virus genomic sequences can be a useful tool not only to provide insight into disease spread and epidemiology, but also for the early identification of viruses with undesirable phenotypic traits from a public health perspective. The BTN3A3-resistant genotype is one of the determinants associated with avian IAV spillover in humans that should be considered in risk-assessment frameworks^54^.

## Materials and methods

### Cells, viruses and chemical treatments

MT4 (a gift from Paul Bieniasz, Rockefeller University) suspension cells were cultured in RPMI (RPMI, Gibco) 1640 medium supplemented with 10% foetal bovine serum (FBS, Gibco), 100 U/mL penicillin and 100 µg/mL streptomycin. Madin-Darby canine kidney (MDCK) cells (ATCC), human embryonic kidney cells (293T) (ATCC), human adenocarcinomic alveolar basal epithelial cells (A549) (ATCC) were cultured in Dulbecco’s modified Eagle’s medium (DMEM, Gibco) supplemented with 10% foetal bovine serum (FBS, Gibco), 100 U/mL penicillin and 100 µg/mL streptomycin. MDCK cells expressing Sialyltransferase 1 (MDCK-SIAT, kindly gifted by Prof John McCauley, The Francis Crick Institute) were further supplemented with 50 µg/mL geneticin. hTERT-immortalized primary human foetal lung fibroblasts were generated at the CVR and cultured in minimum essential medium supplemented with 10% FBS, 100 U/mL penicillin, 100 µg/mL streptomycin and 1x non-essential amino acids. Normal human bronchial epithelial cells immortalised with CDK4 and hTERT (hBEC3-KT)^55^ (UT Southwestern Medical Center) were kept in Keratinocyte serum-free medium (Gibco) supplemented with 50 µg/mL of bovine pituitary extract, 5 ng/mL of human recombinant epidermal growth factor, 100 U/mL penicillin and 100 µg/mL streptomycin. MT4 cells and A549 were authenticated using short tandem repeat (STR) analysis carried out by either the DNA Diagnostics Centre (United Kingdom) or Eurofins (United Kingdom), and analysed using the DSMZ online STR analysis tool.

Culture media of SCRPSY-modified cells was further supplemented with 1 µg/mL of puromycin dihydrochloride. All cell lines were regularly tested for mycoplasma contamination and passaged once or twice weekly.

A/Puerto Rico/8/1934 H1N1 (PR8), A/California/04/2009 H1N1 (Cal04), A/mallard/Netherlands/10-Cam/1999 H1N1 (Mallard), 6:2 reassortants of A/Anhui/1/2013 H7N9 (Anhui H7N9) containing PR8 segments 4 and 6 viruses, and other mutants and reassortants described in this study were generated by reverse genetics essentially as described previously^56^. Briefly, ~2×10^6^ 293T cells were transfected in OptiMEM with pDUAL reverse genetics plasmids (250 ng plasmid for each virus segment), using 4 µL of Lipofectamine 2000 according to manufacturer’s instructions. Twenty-four hours post transfection, the media was changed to serum-free DMEM supplemented with 0.14% Bovine Serum Albumin (w/v) and 1 μg/mL of L-(tosylamido-2-phenyl) ethyl chloromethyl ketone (TPCK)-treated trypsin. Virus-containing supernatant was collected after a further 2-day incubation and propagated in MDCK cells. Clarified supernatant was collected when approximately 90% of cells demonstrated cytopathic effect (typically 36 – 72 h post-infection).

Other virus strains used in the study are the following: A/Udorn/307/1972 H3N2 (Udorn H3N2), A/Norway/3275 2018 H3N2 (Norway H3N2), A/Norway/3433/ 2018 H1N1 (Norway H1N1), A/ruddy shelduck/Mongolia/963V/2009 H3N8 (Ruddy shelduck H3N8), A/wild-duck/Italy/17VIR6926-1/2017 H5N2 (Wild duck H5N2), A/duck/Italy/18VIR4932-2/2018 H7N7 (Duck H7N7), A/turkey/Italy/16VIR8643-54/2016 H9N2 (Turkey H9N2). Udorn H3N2, Norway H1N1, and Ruddy shelduck H3N8 were propagated and plaqued in MDCK, while Norway H3N2 was grown and plaqued in MDCK-SIAT. Avian viruses wild duck H5N2, Duck H7N7 and Turkey H9N2 were propagated and plaqued in MDCK overexpressing *Gallus gallus* ANP32A.

Cycloheximide (CHX) treatment consisted of incubation of cells with 100μg/mL for 1h prior to infection. Virus inoculum, and media used throughout the experiment also contained the same concentration of CHX.

### Arrayed ISG overexpression screening

Lentiviral transduction/flow cytometry-based screening was performed as previously described^57^. Briefly, MT4 cells were seeded in 96-well plates and further transduced with two SCRPSY-based lentiviral vector libraries consisting of 525 human and 345 macaque ISGs. At 48h post transduction, cells were infected with either PR8-GFP, Mallard-GFP or Cal04 7:1 PR8 segment 8 GFP (rescued as previously described^58^). After 10 h, cells were fixed and the percentage of transduced (TagRFP-positive) and IAV-infected (GFP-positive) cells was determined by flow cytometry using a Guava EasyCyte flow cytometer (Millipore).

The screens in figure 2e were performed in a similar manner. MT4, AA2, A549 naïve and overexpressing human ACE2 or 293T suspension cells were transduced with lentiviral ISG-expressing library as described above^59^. Forty-eight hours post transduction, cells were infected with reporter-expressing virus aiming to achieve a 10-50% infection. The panel of viruses used in figure 2e included: AdV, Human mastadenovirus C (Adenovirus 5); PRV, Suid herpesvirus 1 (Pseudorabies); HSV-1, Human herpesvirus 1; BoHV-1, Bovine herpesvirus 1; RVFV, Rift Valley fever phlebovirus; SFTSV, Dabie bandavius (Severe fever with thrombocytopenia syndrome virus); BUNV, Bunyamwera orthobunyavirus; BTV, Bluetongue virus; Rotavirus, Simian Rotavirus A/SA11; CHPV, Chandipura vesiculovirus; VSV, Indiana vesiculovirus; SFV, Semliki Forest virus; CHIKV, Chikungunya virus; HIV-1, Human immunodeficiency virus 1; IAV PR8, A/Puerto Rico/8/1934 (H1N1); IAV Cal04, A/California/04-061-MA/2009 (H1N1) rescued with PR8 segment 8; IAV Mallard, A/Mallard/Netherlands/10-Cam/1999 (H1N1); MeV, Measles Ed-Zag vac; SeV, Murine respirovirus (Sendai virus); PIV5, Mammalian orthorubulavirus 5 (Parainfluenza virus 5 or simian virus 5); hMPV, human Metapneumovirus; hRSV, human orthopneumovirus (human respiratory syncytial virus); bRSV, Bovine orthopneumovirus (bovine respiratory syncytial virus); PIV3, human respirovirus 3 (Parainfluenza virus 3); and Zika virus.

### Influenza A virus infection

Monolayers of A549, MRC5T and hBEC3-KT cells were washed once with PBS and infected with virus diluted in serum-free medium for 1h at 37°C (MOI used is specified in the relevant figures). Medium was replaced with complete maintaining medium (for qRT-PCR or western blot analysis) or serum-free medium supplemented with 0.14% BSA and 1 µg/mL TPCK-treated bovine pancreas trypsin (growth kinetics and quantification of infectious virus titres). Infectious titres were determined by plaque assay.

### Virus quantification by plaque assay

Confluent monolayers of MDCK cells (1×10^6^ or 2×10^6^ cells seeded the day before infection in 12- or 6-well plates, respectively) were washed once with PBS, infected with 10-fold serial dilutions of virus and incubated for 1 h at 37°C to allow virus adsorption to the cells. After virus removal, cells were overlaid with DMEM including 0.14% BSA, 1 µg/mL TPCK-treated trypsin and 1.2% Avicel and incubated for 3 days at 37°C, 5% CO2. After removing the overlay, cells were fixed with PBS/ 4%formaldehyde and stained with a 20% methanol/ 10% acetic acid/ 0.2% Coomassie Blue solution for at least 1h. Staining solution was rinsed under tap water, plates were air-dried and plaques were then counted. Plaque assays for the avian viruses A/wild-duck/Italy/17VIR6926-1/2017 H5N2, A/duck/Italy/18VIR4932-2/2018 H7N7 and A/turkey/Italy/16VIR8643-54/2016 H9N2 were performed as described but using MDCK cells overexpressing galline ANP32A. Plaque assays for Cal04 backbone viruses were performed essentially as described but using MDCK-SIAT cells and a 3-day incubation period at 35°C. Cells were then fixed, fixing solution was removed and cells were washed with PBS and permeabilised using PBS/0.2% (v/v) Triton X-100 for 10 minutes. After two PBS washes, cells were incubated with a mouse anti-NP antibody (diluted 1:1000 in PBS/ 2% BSA) for 1 h on a rocking platform at room temperature. Following two PBS washes, cells were incubated with goat anti-mouse IgG-HRP conjugated secondary antibody (diluted 1:1000 in PBS/ 2% BSA) for 1h in the same conditions. Cells were washed three times with PBS, TrueBlue peroxidase substrate was then added, and cells were incubated at room-temperature covered from direct light. When blue-stained plaques were visible, cells were washed with water, allowed to dry, and plaques were counted (under a stereo microscope, if required).

### siRNA-mediated gene silencing

MCR5T (5×10^5^ cells/well), hBEC3-KT (5×10^5^ cells/we//) or A549-based cells (3.5 x10^5^ cells/well) were seeded before transfection of siRNA molecules targeting BTN3A1, BTN3A3 or Mx1. Silencing of BTN3A1 and BTN3A3 was achieved using commercially available siRNAs: BTN3A1 siRNA1 (Thermofisher Scientific assay ID s21922), BTN3A1 siRNA2 (Thermofisher Scientific assay ID s21923), BTN3A1 siRNA3 (Qiagen material number 1027415 catalogue number SI00163772), BTN3A1 siRNA4 (Qiagen material number 1027415 catalogue number SI03021683), BTN3A3 siRNA1 (Thermofisher Scientific assay ID s20305), BTN3A3 siRNA2 (Thermofisher Scientific assay ID s20306), BTN3A3 siRNA3 (Qiagen material number 1027415 catalogue number SI00093660), BTN3A3 siRNA4 (Qiagen material number 1027415 catalogue number SI00093681). Negative controls were also purchased from the same two companies: Neg ctrl 1 (Thermofisher Scientific catalogue number AM4641), Neg ctrl 2 (Qiagen catalogue number 1022076). Silencing of MX1 was accomplished using a commercially available pool of four different siRNAs (Dharmacon catalogue number L-011735-00) with the respective negative control (Dharmacon catalogue number D-001810-10). When transfecting, 20 pmol of siRNA was used with 3 μL of DharmaFECT 2 (Horizon Discovery) per well according to the manufacturer’s protocol.

### RNP reconstitution minireplicon reporter assay

Sub-confluent monolayers of 293T-Empty and 293T-BTN3A3 cells (2×10^5^ cells seeded in 24-well plates the previous day) were co-transfected in triplicate with 50 ng of each pcDNA3.1+ plasmids encoding PB2, PB1, PA and NP along with 10 ng of transfection control plasmid (CMV-driven expression of Renilla luciferase) and 50 ng of a PolI-driven expression of vRNA or cRNA-like firefly luciferase reporter plasmids. As a negative control, transfections lacking the NP plasmid (empty pcDNA vector was used to balance plasmid intake) were also performed. Two days post-transfection, medium was removed and cells were lysed with 120 µL of reporter lysis buffer. Cell debris was scraped off, lysates were collected into clean tubes and clarified by centrifugation (10,000 rpm for 5 minutes at 4°C) in a benchtop centrifuge. Luminescence was measured from 20 μL of lysate in opaque 96-well plates by using 25 μL of either Luciferase assay reagent II or STOP & Glo® (Dual-Luciferase® reporter assay system) in an automatic injector and plate reader (injection speed: 200 μL/second; gap: 0.5 seconds; integration time: 10 seconds).

### *In vitro* translation assay

Total cellular RNA was isolated using from infected and CHX-treated cells using Qiagen RNeasy Kit according to the manufacturer’s instructions. *In vitro* translation reactions were performed by using 3 μL of RNA in a TNT® Coupled Reticulocyte Lysate system supplemented with 2 μCi of [^35^S] cysteine and [^35^S] methionine mix and incubated at 30ºC for 90 minutes. The reactions were denatured by mixing 1:1 in 4X Laemmli buffer and further heated for 5 minutes at 95°C. Lysates were further analysed by SDS-PAGE followed by Coomassie staining and autoradiography of polyacrylamide gels. Gels were fixed with three 10-minute incubations in a 50% methanol/ 10% acetic acid solution, transferred onto 3mm Whatman filter paper, covered with cling film and dried in a gel dryer for 3h at 80°C for 2 h under vacuum. Dried gels were placed in a sealed cassette with an X-ray film for 48h and films were developed using a Konica SRX-101A X-ograph film processor following manufacturer’s protocol.

### RT-qPCR

Total RNA of CHX treated and/ or infected cells was extracted using the RNAdvance Blood kit (Beckman Coulter Life Sciences), including a DNase treatment, following the manufacturer’s instructions. Extracted and DNase-treated RNA was used to perform strand-specific 2-step qRT-PCR targeting the three different influenza RNA species, as previously described^60^. Briefly, a 5’ tagged primer is used for the cDNA synthesis, the reverse complement of which was then used as a forward primer in the real-time PCR step. Therefore, even in the event of primer-unspecific cDNA synthesis only the cDNA generated with the tagged primer will be detected and amplified in the real-time PCR. cDNA synthesis was performed with 5 µL of extracted RNA using the RevertAid kit (Thermofisher) in a VeritiTM 96 well Thermal Cycler. qPCR reactions were performed with 2 µL of cDNA using the Brilliant III Ultra-Fast qPCR mastermix. qPCR was performed and analysed using the QuantStudio® 3 thermocycler and software systems (ThermoFisher Scientific). A list of primers and probes can be found in supplementary information. *In vitro*-synthesised T7-transcribed viral mRNA, vRNA and cRNA-like transcripts were diluted and used as a standard curve. Relative quantification of viral RNA species was performed against GAPDH.

### Cell fractionation

A549-Empty and A549-HsBTN3A3 cells were infected with PR8 and Mallard at an MOI of 3 for either 45 minutes, 90 minutes or 6 h. Cells were then washed in ice-cold PBS and lysed with 50 mM Tris-HCl pH 7.5, 10 mM KCl, 5 mM MgCl2, 0.5% NP40, 1 mM DTT, 10mM sodium b-glycerophosphate and Halt Protease inhibitor (1X, EDTA-free) (Thermo Fisher 78429). Lysates were kept on ice for ten minutes (gently vortexed every 2-3 minutes) and centrifuged at 1000g for 5 minutes at 4°C. The supernatant (cytoplasmic fraction) was transferred to a clean 1.5 mL tube and the nuclear fraction was washed thrice with the above-mentioned lysis buffer (centrifugations done for 5 minutes at 1000 g). The nuclear fraction was then incubated on ice for 30 minutes with 2X LDS (Thermo Fisher NP0007), 2X sample reducing agent (Thermo Fisher NP0004), Denarase (final concentration 25U/mL) (c-LEcta 20804-100k) and 10 mM MgCl2. The nuclear fraction was sonicated ten times for 20 seconds with 10 second intervals, before being centrifuged at 12000 g for 15 minutes. The supernatant was collected, and the pellet discarded. The cytoplasmic fraction was denatured using 1X LDS and 1X sample reducing agent.

In Fig 4a, given the different times post-infection and the different amounts of accumulated viral proteins between them, the three presented data sets originated from different volumes of lysate and different exposure times.

### Immunoprecipitation

hBEC3-KT cells were infected with Mallard WT and Mallard F313Y at an MOI of 3 for 6h and subsequently lysed^61^. Lysates were pre-clarified overnight at 4°C with protein A agarose beads (Sigma Aldrich P3476). 4 mg of protein lysates were then incubated with 12 µg of either anti-NP or IgG control antibody overnight at 4°C. Supernatants from the α-NP and IgG control were further incubated overnight at 4°C with 12 µg of anti-BTN3A3 and IgG control antibody, respectively. Fifteen µL of Protein-A agarose beads were added for an hour the next morning. IPs were then washed with 50 mM Tris-HCl, 500mM sodium chloride and 0.5% TritonX-100 followed by 50 mM Tris-HCl only before being eluted using 2X LDS and 2X sample reducing agent. Immunoprecipitates were lysed and further subjected to immunoblotting targeting NP, PB2, PB1, PA and BTN3A3.

### Immunoblotting

Total cell, immunoprecipitates or lysates from fractionation experiments were heated at 80°C for 5 minutes and subjected to polyacrylamide gel electrophoresis using 3-(N-morpholino) propanesulfonic acid (MOPS) running buffer and transferred to polyvinylidene difluoride (PVDF) (Merck Millipore IPFL00010) membranes at 30V for 90 minutes. Membranes were blocked for 1h with 1X Tris-buffered saline with 0.2% Tween® 20 (TBST)/5% milk, washed 4 times with 1xTBST and stained with primary antibodies diluted in 5% BSA BSA/0.01% sodium azide overnight at 4°C. After four 10-minute 1xTBST washes, secondary antibodies were diluted in blocking buffer and incubated for 1h at room temperature protected from direct light. Following four more washes in 1xTBST, membranes were imaged using the LI-COR CLx-Odyssey Imaging platform. Densitometry analysis was performed using the Image Studio™ Lite Software.

### Immunofluorescence staining

The desired cell lines were seeded on 13 mm round glass coverslips in 24 well dishes. After infection/ transfection, cells were washed twice with PBS and fixed with 4% formaldehyde in PBS for 20 minutes followed by three PBS washes. Cells were permeabilised with PBS/1% Triton X-100 for 10 minutes at room temperature. Following three washes with PBS, cells were blocked with 1 mL PBS/1% BSA for 1h followed by incubation for 1h with 200 µL of primary antibodies at the appropriate dilutions (for the detection of BTN3A3 in hBEC3-KT cells, the primary antibody was incubated overnight at 4°C). Three 1 mL PBS/0.2% Tween20 washes were then executed to remove unbound antisera followed by incubation of secondary antibodies in the same volume. 4’,6-Diamidino-2-phenylindole (DAPI) (100 ng/mL) was diluted in blocking buffer and incubated for 10 minutes. When using HCS CellMask™ Deep Red Stain (2 µg/mL – ThermoFisher Scientific), this was added after DAPI staining for 30 minutes followed by three PBS washes. After labelling steps were completed, coverslips were washed three more times with water and mounted upside down on glass slides with AF1 Mounting media (CitiFluor™). Coverslips were imaged using the ZEISS LSM 710 or LSM 880 (with Airyscan) confocal laser scanning microscopes. Quantification of nuclear and cytoplasmic proteins from confocal images was performed using a pipeline created on CellProfilerTM. Pipeline details are provided in supplementary information.

### Immunohistochemistry

We analysed sections (3 μm thick) of formalin-fixed and paraffin-embedded (FFPE) healthy human lung (n=2) and respiratory nasal epithelium tissues (n=5). Health tissues were purchased from Amsbio (USA) and were obtained from 5 female and 1 male donors ranging in age between 31 and 62 years old of Caucasian and Asian ethnicity (1 of them of unknown ethnicity). Tissues were stained with a rabbit anti-BTN3A3 antibody (HPA 007904, Atlas antibodies) for 90 minutes at room temperature after pressure cooking in sodium citrate. For visualisation, EnVision Detection System HRP, peroxidase/DAB (rabbit, K500711, Agilent) was used according to manufacturer’s instructions. The specificity of the antibody was validated on FFPE A549-Empty and A549-HsBTN3A3 cell pellets. Negative control sections included an isotype control on a consecutive serial section. Slides were scanned with a Leica Aperio Versa 8 slide scanner (Leica Biosystems) and images were acquired with a Aperio ImageScope software (Leica Biosystems).

Mouse lung tissues were stained with a rabbit anti-BTN3A3 antibody (HPA 007904, Atlas antibodies) and anti-GFP antibody (Cell Signalling 2555S) for 90 minutes at room temperature after pressure cooking in sodium citrate. For visualisation, EnVision Detection System HRP, peroxidase/DAB (rabbit, Agilent K500711) was used according to manufacturer’s instructions.

### AAV vectors

AAV vector genome plasmids were engineered to encode a ubiquitous CASI promoter^62^ driving expression of the human BTN3A3 gene followed by a WPRE^62^ and a SV40 polyA signal all contained between AAV2 inverted terminal repeats. AAV vectors were produced by co-transfection of human embryonic kidney 293 cells with genome and packaging plasmids as described previously^62^. Vectors pseudotyped with AAV6.2FF were purified by use of a heparin column^62^. AAV vector titres were determined by quantitative polymerase chain reaction (qPCR) analysis as described elsewhere^63^.

### Mouse infections

Six-week old female C57BL/6 mice were purchased from Envigo (UK). Animals were maintained at the University of Glasgow under specific pathogen free conditions in accordance with UK home office regulations (Project License PP1902420) and approved by the University of Glasgow ethics committee. Following 7 days of acclimatization, the mice were briefly anesthetized using inhaled isoflurane and transduced with 1×10^11^ virus genomes of adeno-associated virus (AAV). Mice received either AAV-GFP or AAV BTN3A3 in 50 µL PBS intranasally. Twenty-one days post AAV instillation mice were infected with Cal04 and Cal04 V313F (both 6:2 with PR8 HA/NA). Group sizes were chosen on the bases of a pilot experiment to assess AAV transduction and previous experience^64^. No specific randomisation were conducted. Analysis of data was conducted in an unbiased manner but no specific blinding was used for the researchers carrying out virus titration from mice tissues. Mice were briefly anesthetized using inhaled isoflurane and intranasally infected with 5 x10^3^ PFU of each virus in 20 μL of PBS. A total of 4 infected mice displayed technically poor infections with bubbles forming from the nostrils and therefore were discarded from the experiment. All mice were euthanized by cervical dislocation 3 days post infection. Weight loss for all groups did not exceed the humane cut-off point of 20%. Once euthanised, mouse lungs were extracted and frozen at -80°C. Lungs were thawed, weighted and homogenised with 1 mL of DMEM. Homogenates were cleared by centrifugation (3000 rpm, 4°C) and titrated by plaque assay.

### *In silico* identification of BTN3 homologs

To identify proteins expressed by other species, homologous to the human BTN3 genes, we performed a Blastp search (v.2.8.1) against all available members of the Haplorrhini suborder in the NCBI blast protein refseq database version 5 (e value cutoff 1e-60, as of 6th of April 2021)^65^. The human BTN3A3 protein sequence (NP_008925.1) was used as a probe for the BLAST search. The isoform with the longest sequence was kept for each protein product annotated with the same name. Similarly, Blastp with human BTN3A3 was used for identifying proteins expressed by non-primate species susceptible to IAV infection: *Gallus, Anas platyrhynchos, Equus caballus*, and *Sus scrofa* and more distant human paralogues (Extended Data Fig. 2, Supplementary Table 5).

Protein members of the butyrophilin 3 subfamily retrieved from the BLAST search were manually cross-checked with proteins in the Ensembl database^66^ and if the protein sequences were not identical between the two databases the sequence with highest similarity to the human BTN3A3 protein sequence was retained. A total of 30 proteins were retrieved from the following species: Pan troglodytes, *Cebus imitator, Equus caballus, Homo sapiens, Gorilla gorilla gorilla, Chlorocebus sabaeus, Macaca mulatta, Pongo abelii, Carlito syrichta, Mandrillus leucophaeus, Callithrix jacchus, Nomascus leucogenys, Rhinopithecus roxellana*.

A custom set of Pfam hmm profiles were used for identifying the conserved domains in the proteins, comprising the immunoglobulin V-set domain (PF07686), CD80-like C2-set immunoglobulin domain (PF08205), PRY (PF13765) and SPRY (PF00622) domains. All protein sequences were scanned with the profile set using hmmscan (HMMER 3.3)^67^. The best hit for each identified domain was extracted from the protein sequence aligned with the respective domain segments using mafft (v7.453, -- maxiterate 1000 --localpair)^68^. Protein alignments were converted to codon alignments using pal2nal^69^. Phylogenies for each separate domain alignment (Extended Data Fig. 4) and concatenated domain sequences (Extended Data Fig. 2) were reconstructed using iqtree (version 1.6.12)^70^ with the best suited substitution model selected by the iqtree ‘-m TEST’ option and 10,000 ultrafast bootstrap replicates.

### IAV phylogenetic analysis

A total of 35,477 full-length NP coding sequences unique on the nucleotide level (identical sequences collapsed) were retrieved from the NCBI Flu database (https://www.ncbi.nlm.nih.gov/genomes/FLU/Database/nph-select.cgi?go=database), as of the 8th of June 2021, sampled until the end of 2020), only including type A influenza sequences annotated to have been isolated from avian, canine, equine, human and swine hosts. Sequences with ambiguous nucleotides and internal stop codons were removed, resulting in a dataset of 34,079 sequences. The corresponding protein sequences were aligned using mafft (v7.453, --maxiterate 1000 --localpair)^68^ and then converted to a codon alignment with pal2nal^69^. Metadata associated with each sequence accession were retrieved and tabulated. Numbering of NP amino acid residues was assigned based on the PR8 sequence. Therefore, the 6 amino acid N-terminal extension of 2009 pH1N1 viruses were not considered for the residue numbering (i.e., amino acid residue M6 was considered M1).

To reduce oversampling of related sequences the dataset was clustered with a minimum sequence identity of 0.99 using MMseqs2 (--min-seq-id 0.99 --cov-mode 0)^71^. One representative was kept from each cluster leading to a filtered dataset of 14,665 sequences. The codon alignment of the filtered set was used to reconstruct a phylogeny with iqtree under a GTR+I+G4 model (selected as the most appropriate model with the ‘-m TEST’ option)^70^. The resulting phylogeny was then time-calibrated using TreeTime^72^ (Fig. 3c). Eleven sequences with annotated dates inconsistent with the root-to-tip regression were subsequently excluded from the analysis.

To explore the H7N9 epidemic NP clade in more detail, representative sequences from this broader avian NP clade (Fig. 5c) as well as the unfiltered sequences from each representative’s corresponding cluster were retrieved (3,150 sequences). The codon alignment of these NP sequences was used to infer a more detailed maximum likelihood phylogenetic reconstruction of this particular clade (iqtree under a GTR+I+F+G4 model with 10,000 Ultrafast bootstrap replicates)^70^ and time-calibrated using TreeTime as described above^72^ (Fig. 5c). All phylogenies were visualized using the ggtree R package^73^, unless stated otherwise. Tree statistics were analysed using the ete3 Python package^74^.

### GISAID sequence analysis

Protein sequences from atypical, avian-only serotypes (H1-H18) were retrieved from the GISAID database (http://gisaid.org/) for avian and human hosts (Supplementary Tables 2, 4). Isolates were filtered for having all 8 segments and having been sampled until the 1^st^ of January 2023. The NP, HA and PB2 proteins were downloaded for each isolate and aligned using mafft (v7.453, -- maxiterate 1000 --localpair)^68^. NP and PB2 residues at sites of interest and HA polybasic cleavage site presence (identified as having 3 or more K/R residues at the corresponding region) were summarised along with sequence metadata using Python3. GISAID acknowledgments for all analysed sequences are provided in Supplementary Tables 7-8.

### Biosafety considerations

All experiments were performed in accordance with national regulations. Work was approved by the local GM Safety Committee (University of Glasgow GM Centre 223) and licensing authorities of the United Kingdom (Health & Safety Executive). To investigate the determinants of BTN3A3 resistance, mutations were introduced into either the low-pathogenicity avian strain H1N1 Mallard (A/mallard/Netherlands/10-Cam/1999, H1N1), the human strain Cal04, PR8, or a (6+2) reassortant of an avian H7N9 strain with PR8. The H7N9/ PR8 reassortant contained the ‘internal’ genes from H7N9 and the ‘external’ (glycoprotein) genes from PR8. PR8 is a human IAV strain that is greatly attenuated due to extensive laboratory passage in the mouse^75,76^. In terms of biosafety, two classes of mutation were designed. The first class of mutation aimed to convert a BTN3A3 sensitive phenotype into a resistant one. This was only carried out for the low-pathogenicity avian H1N1 Mallard strain. As noted in the Results section, many circulating avian lineages already possess a BTN3A3 resistant genotype and therefore none of the mutants we generated pose a greater biosafety risk than viruses already in circulation. The second class of mutation aimed to convert a BTN3A3 insensitive phenotype into a sensitive one. These mutations, which were carried out with PR8, Cal04 and H7N9/PR8 strains, would be expected to attenuate viral growth in humans still further and would therefore reduce biosafety risks.

Experiments with IAV described above were carried out at Biosafety Level 2. Most of the fluorescent viruses used in Fig 2e were also used in Biosafety Level 2. Experiments performed with SFTSV, CHIKV, SARS-CoV2, BTV-8 and HIV-1 were conducted at Biosafety Level 3.

### Reproducibility

Original source images for data obtained by electrophoresis, shown as cropped images in the figures of this study, are presented in Supplementary Figure 1. Images shown throughout the manuscript are representative images of experiments as follows. Western blotting in Figure 1c shows similar levels of BTN3A3 knockdown using four distinct guides in two different cell lines. Figure 1c shows one of three independent experiments, which were then analysed in panel d. The micrographs of Figure 1e show a field of tissue sections collected from healthy nasal or lung tissues from one donor and probed with BTN3A3 or isotype control antibodies. Images are representative of several other fields of the same section. Similar images were obtained in the same experiment from four other donors for the respiratory nasal epithelium and one additional donor for the lungs. Results obtained in the lungs were further validated by the transcriptomic data shown in ED Figure 1c-d. Western blotting in Figure 2a was carried out to show overexpression of the respective BTN3 proteins, and it shows the specificity of antibodies against such proteins. These and other anti-BTN3 antibodies were then used for several experiments (Figures 3e, 4a, 4c, ED Figure 1a, e, ED Figure 3, ED Figure 8, ED Figure 9). Image of western blotting in Fig. 2c is representative of two independent experiments. Images in Figure 3e are western blots carried out once to validate the expression of Mx1 and BTN3A3 in stable cells then used for experiments shown in Figure 3f. Micrographs in Figure 3g are representative images of lung sections from single mock-, AAV-GFP- and AAV-BTN3A3-transduced mouse lungs. Similar images were obtained in 9 additional mice (a total of three mice per condition). Images from western blots of Figure 4a and d are representative of three independent experiments. Western blot images in Figure 4c were performed once and represent expression controls of one of the three experiments shown in Figure 4b. Micrographs in panel 4e are representative images of 4 independent experiments (further analysed in Figure 4i-j). Western blotting in ED Figure 1a shows similar levels of BTN3A1 knockdown using four distinct guides in two different cell lines. ED Figure 1 shows one of three independent experiments that were analysed in panel b. Western blots in ED Figure 1e represents an experiment showing expression of BTN3A3, RSAD2 and pSTAT1 following IFN treatments in four different cell lines. Western blotting in ED Figure 3b was performed from one of the two independent experiments carried out in ED Figure 3a. Image of western blotting in ED Figure 7a shows the siRNA-driven Mx1 knockdown of one of the three experiments carried out in ED Figure 7b. Western blotting in ED Figure 8b was performed from one of the three independent experiments carried out in ED Figure 8a and validates the expression of RNP proteins. ED Figure 9a, b and c are representative images of western blotting of three independent experiments. Micrograph in ED Figure 10a is a representative image of 4 independent experiments (further analysed in panels b and c).

## Extended Data

## Supplementary Material

Peer Review File

Reporting Summary

Supplementary Discussion

Supplementary Figure 1

Supplementary Figure 2

Supplementary Table 1

Supplementary Table 2

Supplementary Table 3

Supplementary Table 4

Supplementary Table 5

Supplementary Table 6

Supplementary Table 7

Supplementary Table 8

Supplementary Table 9

## Data Availability

Alignments and raw phylogenetic data related to this study can be found in the following GitHub repository: https://github.com/spyros-lytras/BTN3A3_IAV. Source data related to the animal experiments illustrated in Fig. 3h are available in Supplementary Table 9. Gel source data are available in Supplementary Figure 1.
