# Supplementary material for: BTN3A3 evasion promotes the zoonotic potential of influenza A viruses
Source: Nature. Author manuscript; Available in PMC 2026 Oct 15. (PMC7619554; doi:10.1038/s41586-023-06261-8)
Supplement: Supplementary file 1 [file EMS219322-supplement-Peer_Review_File.pdf]

Peer Review File

Manuscript Title: BTN3A3 evasion promotes the zoonotic potential of influenza A viruses

Reviewer Comments & Author Rebuttals

Reviewer Reports on the Initial Version:

Referees' comments:

Referee #1 (Remarks to the Author):

This paper is terrific. A tour de force in many ways, and there is no doubt that the authors have identified an important restriciton factor. With that important point made (fantastic work!) I want to focus on what I consider to be a mjor issue with the paper.

Really the most exciting and practical application of this work would be if, as the authors argue, the mutations in NP they identify that permit a degree of escape from BTN3A3 restriction were indeed useful for predicting successful spillover into humans. Here, I will define successful spillover as not just infection of a human but establishment of a new viral lineage in humans, obviously with human to human transmission potential.

How do the authors assess this potential? First, they note that several avian-origin viruses isolated from humans do indeed contain NP mutations that provide BTN3A3 escape. Indeed, the title of their paper pretty much implies (to my way of reading it) that all avian flu viruses that jump into humans evade BTN3A3 restriction

“Zoonotic avian influenza viruses evade human BTN3A3 restriction"

However, as the authors note, but do not do an adequate job of explaining, the majority of the isolates that the authors considered actually do not evade BTN3A3 restriction, the H5N1 isolates. This can be seen in Fig. 6D, but is obscured by using % on the Y axis instead of number of cases.

So the title is problematic and should at the least have “Some” added to the beginning.

Next, there is a huge issue with ascertainment bias here. Isolates from humans of avian-origin flu viruses are tiny in number (less than 300) compared to the number of such cases that seroprevlance estimates suggest must have occurred (at least in the 100s of thousands). See, for example, the high seroprevalence of H5N1 and H9N2 in Egyptian poultry workers:

https://academic.oup.com/jid/article/211/9/1399/853445
H5N1 and H9N2 in Egyptians

And an estimated 0.077% seroprevalence of H7N9 in humans in China:

https://www.ncbi.nlm.nih.gov/pmc/articles/PMC7431636/

That's about a million people.

So, what can we learn from this? First, the vast, vast, vast majority of avian-origin flu cases in humans is unascertained, likely because they are mild. And, second, there have been ENORMOUS numbers of human infections with BTN3A3-evading mutations that have not caused successful spillover. Indeed, every single one the we know of, to date.

BTN3A3 evasion is likely a very good predictor of increased pathogencity in humans. But it is a very poor predictor of which flu viruses are likely to successfully spillover and cause the next pandemic. (And the fact that there is VERY limited transmission of H7N9 human to human does not change that.)

Referee #2 (Remarks to the Author):

This work identifies BTN3A3 as potent restriction factor of avian influenza viruses (IAVs) in human cells which targets the viral nucleoprotein (NP) by recognizing a domain that includes amino acids at positions 313 and 52. Unlike avian IAVs that have an F residue at position 313, human IAVs have a Y at this position and thus escape restriction by BTN3A3. The authors further show that avian IAVs such as the H7N9 strain which recently spilled over into humans evade BTN3A3 restriction by having substituted the Y at position 52 in NP by an N residue. These novel findings are fascinating and provide new insights into the mechanisms that can restrict zoonotic infections of humans with avian IAVs. This new knowledge will greatly contribute to future risk assessment studies of emerging IAVs.

Major criticism:

1. Although the authors set out to identify interferon-induced genes (ISGs) with anti-flu activity, they eventually identified restriction factor BTN3A3 that is constitutively expressed in the human respiratory tract. It thus appears that BTN3A3 was included in the ISG library for wrong reasons. This fact deserves more discussion: Is BTN3A3 indeed part of the innate immune system or does it represent an intrinsic (non-regulated) restriction factor affecting influenza viruses? Some additional experiments should be performed with human primary airway epithelial cells to determine whether the BTN3A3 gene is regulated to some extent by type I or type III IFN.

2. The data set presented in figure 5 is not sufficient evidence for the conclusion that BTN3A3 restricts sensitive viruses at the level of transcription. Available results indicate that the earliest steps of the replication cycle such as particle entry and migration of vRNPs to the nucleus are not affected. However, it remains unclear whether the interaction of BTN3A3 with NP of sensitive viruses affects viral polymerase activity. This important mechanistic question should be clarified by measuring primary viral transcription (viral polymerase activity at early times post infection in the presence of cycloheximide) in control cells and cells overexpressing BTN3A3.

3. The notion that BTN3A3 can restrict influenza viruses in vivo is not supported sufficiently well by

the data presented in figure 4g. The experiment in which mice were transduced with BTN3A3-expressing lentivirus shows that only two of five animals had reduced viral titers in the lungs on day 3 post-infection with the sensitive virus. Obviously, a larger number of animals should be tested to strengthen this important claim.

4. It would be of interest to know whether the BTN3A3 is polymorphic and whether patients suffering from accidental infection with avian H5N1 or H7N9 flu viruses carry altered BTN3A3 alleles. Available sequencing data deposited in public libraries should be analyzed.

Minor issues:

- Data shown in suppl. figs 1a and 1b are not included well in the story. What is the reason for the high IFN resistance of the mallard virus? Further, the legend says that IFN-λ was used for the experiment shown on the right hand panel of figure 1a. However, the panel says it was IFN-γ. What is correct?

- The true number of ISGs tested in this study is difficult to reconcile from the illustration shown in Figure 1a. The drawing suggests that 871 (527+344) ISGs were tested. However, according to the text above the drawing a total of 752 ISGs were analyzed. What is correct?

- Some of the viruses listed in Fig. 2e are not grouped correctly: CHIKV is a positive-strand virus, whereas VSV is a negative-strand virus.

- Line 376: reference 27 is not suitable to back up this statement.

- Material and Methods section. Please describe the characteristics of the siRNAs targeting Mx1, as well as BTN3A3 (1-4), or provide references.

- Text page 7, lines 25 and 26. The statement „Avian-to-human mutation F313Y....... exhibits increased Mx1 sensitivity“ is wrong. The opposite is true.

- The Mx nomenclature is not consistent throughout the text and figures (Mx1, Mx-1). Please adhere to the rules.

Referee #3 (Remarks to the Author):

IFN-stimulated genes (ISGs) control viral infection, and their rapid evolution has implicated them in regulating cross-species transmission of viruses. Here, Pinto, et al. investigate the activity of primate ISGs in regulating replication of human- and avian-origin influenza viruses. By performing a high-throughput screen of ISGs for anti-influenza activity, they identify BTN3A1 and BTN3A3 as genes that selectively impair influenza viruses from avian hosts. A convincing and well-controlled series of experiments validates the ability of BTN3A3, and to a lesser extent BTN3A1, to block infection and replication by avian influenza isolates, but not human influenza isolates or any of the 24 diverse

viruses they tested. Phylogenetic analyses indicate that the BTN3A locus underwent gene duplication and diversification, with the antiviral phenotype arising after new and old-world primate lineages diverged. While the authors clearly identified paralogs with antiviral activity only in old-world monkeys and apes, the underlying changes that convey antiviral activity are not delineated. Evolutionary analysis also mapped resistance to BTN3A3 to a specific residue in the viral nucleoprotein (NP) in circulating human influenza isolates. Interestingly, a distinct escape variant is present in H7N9 viruses that have spilled over to humans, demonstrating convergent evolution. These results highlight the novelty of the authors' discovery of BTN3A3 as a restriction factor and the importance of evading BTN3A proteins during cross-species transmission. The authors express BTN3A3 in mouse lungs to test in vivo relevance, which is laudable, but the results are indeterminant. In addition, mechanistic studies suggest that BTN3A3 interferes with the viral replication machinery, but the data fail to identify exactly where in the life cycle this occurs, how BTN3A3 functions to restrict influenza virus, and why this is specific to avian isolates. Thus, the identification, validation and genetic interactions between BTN3A and influenza viruses are generally clearly defined and well supported. However, the in vivo relevance and mechanism of restriction are unresolved.

1. AAV vectors are used to deliver BTN3A3 to the lungs of mice to test its roles in vivo. The authors achieve impressive transduction of these animals. Despite high levels of BTN3A3 expression, only 2 of 5 animals demonstrate reduced viral titers when infected with susceptible virus. The remaining 3 animals showed titers indistinguishable from resistant virus. Moreover, these results are significant when the correct comparisons are made. It would appear more relevant to compare titers from resistant virus (Cal04) to sensitive virus (Cal04 V313F) in BTN3A3 transduced mice, as this directly addresses the strain-specific inhibitory activity of BTN3A3.
2. The mechanism by which BTN3A3 inhibits the viral replication machinery, when this occurs during infection, and how strain-specificity is conferred all remain unresolved. The authors show that the early stages of infection (up to nuclear import of vRNPs) are unaffected by BTN3A3, leading them to investigate its effect on vRNP activity. However, they use indirect measures to conclude that BTN3A3 blocks transcription. vRNPs template both transcription and replication. Given that newly replicated genomes can also serve as templates for further transcription, the levels of replication and transcription are intimately linked. The assays deployed by the authors cannot delineate a specific effect on transcription. Further, none of the experiments shed light on how BTN3A3 achieves its antiviral activity.
a. the authors claim a decrease in mRNA levels at early time points post-infection for susceptible virus in BTN3A3-expressing cells, presumably before replication has begun (Fig 5c). However, this reduction is not significant until 4hpi – the only time point where mRNA is significantly lower – and well after genome replication has occurred. If anything, the more pronounced and consistent reduction in cRNAs at all times during infection might point to a defect in replication.
b. Well-developed infection assays exist to selectively test viral transcription in the absence of genome replication or genome replication in the absence of viral transcription. Data along these lines are needed to support claims that BTN3A3 blocks transcription.
c. pDUAL vectors are used to express viral proteins in minireplicon assays. This adds a serious confounding effect. pDUAL expresses mRNA and genomic vRNA. Functional polymerases complexes will thus act not only on the reporter gene of the minireplicon assay, but also on these additional vRNAs to amplify the polymerase and NP itself. This self-amplification can artificially inflate

differences between functional and restricted polymerase complexes. Oddly, while the minireplicon assays report differences in polymerase activity (Fig 5b, ED Fig 11a-b), this is not reflected in blotting for viral proteins that might be expected to also show similar patterns if they are expressed from pDUAL (ED Fig 11c). There appears to be a disconnect between these two sources of data and the use of pDUAL vectors adds unnecessary complications.
d. While BTN3A3 clearly shows preference in restricting avian-style NP, how specificity if conferred is unknown. Immunoprecipitation shows BTN3A3 interacts equally well with sensitive WT Mallard RNPs and resistant Mallard RNP F313Y (ED F12c).
e. While resistance maps to NP, the authors never show that BTN3A3 interacts with NP. Interactions studies in ED Fig12 included all viral proteins.
3. Evolutionary analyses and functional assays identify a collection of primate paralogs with and without antiviral activity. Yet, the evolutionary difference(s) that confer the antiviral phenotype are not identified. This is particularly intriguing as their phylogeny makes it appear that this activity might have arisen 4-5 independent times.
4. Control blots verifying equivalent protein expression are missing in several experiments. In some cases, these are essential to confirm the protein is actually expressed when the authors report that a paralog does not have antiviral activity. See Fig 3b, 3c, ED Fig 4d, Fig 4e, Fig 6a.

Minor:
1. The authors nicely show that the antiviral activity of BTN3A3 and MX1 function independently of each other, even if they both act on the same sites of NP. Nonetheless, caution is warranted in the discussion of viral evolution, as evolutionary pressure causing changes at NP 52/313 are likely some combination of BTN3A3 and MX1.
2. Fig 6h models structural differences for various avian NPs. Modelling is not necessary for 52Q/313F as several structures have been solved for avian NP (e.g. 2Q06, 7DKG, 7DXP. These structures show inherent flexibility of sidechains at this position.
3. Page 14 line 25 – please clarify that these data are present in Fig 2e, not 3a.
4. Fig 6 legend lacks notations for panel 6f, which is misassigned at a duplicated 6e.
5. When referring to NP from 2009 pH1N1, please note how amino acid numbering was assigned. 2009 pH1N1 NP contains an N-terminal addition relative to all other NPs which can confuse numbering when compared to other NPs.
6. Page 11 Line 11 – please clarify, as non-human primates can also be naturally infected with IAV (Karlsson et al. 2012 DOI: 10.3201/eid1809.120214).

Author Rebuttals to Initial Comments:

Reviewer #1

We thank Reviewer # 1 for their supportive statement "*This paper is terrific. A tour de force in many ways, and there is no doubt that the authors have identified an important restriction* factor…"

The referees raised some criticism that we address below.

... Here, I will define successful spillover as not just infection of a human but establishment of a new viral lineage in humans, obviously with human to human transmission potential.

We define "spillover" differently from the reviewer. We considered spillover a simple transmission event of an animal virus in humans with no or limited subsequent human-to-human transmission. The establishment of a new lineage would require further human "adaptation" which, in the case of influenza A viruses, it has solely happened following a pandemic event. This terminology is the one used by others (*e.g.* https://pubmed.ncbi.nlm.nih.gov/27834632/) although scientists of different communities may use the term in different contexts.

Despite the differences in terminology, "spillover" (as we define it) is a critically important event as it (i) can have a severe (even lethal) impact on human health (e.g. in 2013 an H7N9 variant caused more than 600 deaths) and (ii) it is the necessary first step for the establishment of a pandemic virus.

Our revised manuscript articulates this point at page 3, lines 1-7:

"*Occasionally, spillover of avian IAV into humans may result in severe or even lethal disease[9]. For example, in 2013 an H7N9 variant, resulting from multiple reassortment events of different avian viruses[10], caused more than 600 deaths in humans[11] and has since re-emerged in different outbreak waves[12]. These spillover events are not typically followed by extensive human-to-human transmission chains, but they are a risk to global health as they could enable the first step towards adaptation to the human host and the generation of pandemic IAV strains[1].*"

Really the most exciting and practical application of this work would be if, as the authors argue, the mutations in NP they identify that permit a degree of escape from BTN3A3 restriction were indeed useful for predicting successful spillover into humans….How do the authors assess this potential? First, they note that several avian-origin viruses isolated from humans do indeed contain NP mutations that provide BTN3A3 escape. Indeed, the title of their paper pretty much implies (to my way of reading it) that all avian flu viruses that jump into humans evade BTN3A3 restriction: "Zoonotic avian influenza viruses evade human BTN3A3 restriction"
However, as the authors note, but do not do an adequate job of explaining, the majority of the isolates that the authors considered actually do not evade BTN3A3 restriction, the H5N1 isolates. This can be seen in Fig. 6D, but is obscured by using % on the Y axis instead of number of cases. So the title is problematic and should at the least have "Some" added to the beginning.

We agree with the reviewer that the dataset presented in our original submission (including fig 6D) was not the optimal reflection of the scientific importance of our findings. Since then, we have been able to significantly expand our dataset by downloading from the GISAID EpiFLU database all viral sequences of human isolates of serotypes normally circulating in avian species. As mentioned above, compared to the 253 isolates analysed in the original submission, we have now investigated ~1700 sequences. Moreover, chronological associations between circulating avian IAV with BTN3A3-resistant genotypes and human avian IAV infections has been seen for many minor and major spillover events, further reinforcing the need for BTN3A3 resistance to facilitate avian-to-human transmission of avian IAV.

The number of sequences and the results are now far more compelling and are articulated at Pag. 11, line 16 to Pag 13, line 7 and illustrated in Fig. 6 (especially panel 6g) and Extended data Figs.

S13 and S14). In the interest of the readability of this rebuttal letter we haven't cut and pasted this section of the manuscript here.

In summary the updated body of work provides more lines of evidence supporting the role of BTN3A3 resistance in the zoonotic potential of many avian IAV, with the only exception being HPAI H5N1 (also discussed in detail in the revised manuscript). These are synthesised in the Discussion (pag. 14, Line 7) as follows.

"There are several independent lines of evidence pointing to evasion of BTN3A3 as a key risk factor for the zoonotic potential of IAV. First, all the human endemic IAV viruses, including those that emerged in 1918, are resistant to BTN3A3. Second, the swine H1N1 2009 pandemic virus originated from a BTN3A3 resistant NP 313V clade, that we estimate appeared in pigs between 2002 and 2006, ahead of the start of the 2009 pandemic (Extended Data Fig. S7). Third, evasion of BTN3A3 restriction is a conserved trait of the majority of avian viruses that have successfully spilled over into humans, due to a mutation from a BTN3A3-sensitive 52Y residue to a BTN3A3-resistant N, Q or H residue (or very rarely in H3N8 through the F313V mutation) and further hypothesise that residue 52 masks the effect of 313F given the proximity of the two residues in the available NP crystal structures[4]. Finally, we showed that avian IAV circulating in birds showed an increased frequency of the BTN3A3-resistant genotype in 2011-2012, ahead of the initial H7N9 outbreak in humans. Indeed, it is only around 2013, when the first human H7N9 cases were identified, that the NP 52N residue became dominant in this lineage."

We recognise that we had a portion of the text in the original submission that could have been misunderstood as giving the impression that BTN3A3 was "the" only barrier to avian IAVs spillover. This is not the case. As described in the manuscript, there are several barriers hampering cross-species transmission of avian IAVS in humans. BTN3A3 is just one of them. Presumably, successful spillover events may occur with viruses that can overcome some of these blocks. While successful adaptation and sustained human-to-human transmission would occur only when all of these blocks can be overcome. Hence, we changed the title to reflect the point made by the reviewer and what we highlight above. The new title now reads: "Evasion of BTN3A3 is a risk factor for the zoonotic potential of influenza A viruses"

Next, there is a huge issue with ascertainment bias here. Isolates from humans of avian-origin flu viruses are tiny in number (less than 300) compared to the number of such cases that seroprevlance estimates suggest must have occurred (at least in the 100s of thousands). See, for example, the high seroprevalence of H5N1 and H9N2 in Egyptian poultry workers: https://academic.oup.com/jid/article/211/9/1399/853445

H5N1 and H9N2 in Egyptians And an estimated 0.077% seroprevalence of H7N9 in humans in China:

https://www.ncbi.nlm.nih.gov/pmc/articles/PMC7431636/

That's about a million people. So, what can we learn from this? First, the vast, vast, vast majority of avian-origin flu cases in humans is unascertained, likely because they are mild. And, second, there have been ENORMOUS numbers of human infections with BTN3A3-evading mutations that have not caused successful spillover. Indeed, every single one the we know of, to date.

BTN3A3 evasion is likely a very good predictor of increased pathogenicity in humans. But it is a very poor predictor of which flu viruses are likely to successfully spillover and cause the next pandemic. (And the fact that there is VERY limited transmission of H7N9 human to human does not change that.)

We agree that the reviewer was right to make this statement on the bases of the data presented in the original manuscript. We agree that for every symptomatic virus infection for which we have sequences information, there will be many others for which we don't have sequences at all, and these are mostly clinically asymptomatic infections that can be identified with serological surveillance (but lack sequencing data). As result, the percentage of seroprevalence will always be higher than the available number of sequences, as per the serology studies provided by the referee. However, the provided examples refer to serological studies carried in the context of infections with either a highly pathogenic H5N1 (one that we show do not necessarily require a BTN3A3 resistance genotype for spilling over) or for H7N9 and H9N2. The latter two represent LPAI viruses with nearly 100% of BTN3A3 resistance in human-isolated viral sequences.

Moreover, in the revised manuscript we analysed avian viruses circulating in birds. For H7N9 and H9N2 viruses, more than 90% of birds-isolated sequences from 2013 onwards (the date of the first wave of H7N9 in humans) possess a BTN3A3-resistant genotype (data obtained from 900 sequences for H7N9 and 3258 for H9N2).
Hence, all the most recent analyses suggest that BTN3A3-resistance is a feature of most viruses that are able to spillover in humans.

Referee #2 (Remarks to the Author):

We thank the reviewer for his helpful comments and supportive statements *"These novel findings are fascinating and provide new insights into the mechanisms that can restrict zoonotic infections of humans with avian IAVs. This new knowledge will greatly contribute to future risk assessment studies of emerging IAVs."*

The referee made the following constructive criticism.

Major criticism:

1. Although the authors set out to identify interferon-induced genes (ISGs) with anti-flu activity, they eventually identified restriction factor BTN3A3 that is constitutively expressed in the human respiratory tract. It thus appears that BTN3A3 was included in the ISG library for wrong reasons. This fact deserves more discussion: Is BTN3A3 indeed part of the innate immune system or does it represent an intrinsic (non-regulated) restriction factor affecting influenza viruses? Some additional experiments should be performed with human primary airway epithelial cells to determine whether the BTN3A3 gene is regulated to some extent by type I or type III IFN.

BTN3A3 is indeed an ISG and it is included in our ISG libraries because it has been identified in our published comparative interferome study (doi: 10.1371/journal.pbio.2004086). Data in the study can be mined using our online tool (http://isg.data.cvr.ac.uk/) that will show upregulation of BTN3A3 mRNA of 2.26 log2FC in response to type-I IFN.

However, as requested by the reviewer, we have also tested 4 different human pulmonary cell lines (Extended data Fig. S2) and by western blotting showed that BTN3A3 is not only constitutively expressed in all 4 cell lines but also upregulated by both type-I and type-II IFN. Hence, BTN3A3, like other virus restriction factors (TRIM5 and TRIM22 for example) can be considered both an intrinsic and an innate immune factor.

2. The data set presented in figure 5 is not sufficient evidence for the conclusion that BTN3A3 restricts sensitive viruses at the level of transcription. Available results indicate that the earliest steps of the replication cycle such as particle entry and migration of vRNPs to the nucleus are not affected. However, it remains unclear whether the interaction of BTN3A3 with NP of sensitive viruses affects viral polymerase activity. This important mechanistic question should be clarified

by measuring primary viral transcription (viral polymerase activity at early times post infection in the presence of cycloheximide) in control cells and cells overexpressing BTN3A3.

We thank Reviewer # 2. This comments were very useful and indeed correct. We have carried out two different additional experiments. The first one is by testing primary transcription using cycloheximide (CHX, a translation elongation inhibitor). With this experiment, A549-Empty and A549-BTN3A3 cells were pre-treated with CHX and subsequently infected with Mallard WT or F313Y. Viral mRNA was left to accumulate for 12h, RNA was extracted and applied into reticulocytes lysates supplemented with ^{35}S-tagged amino acids. In these lysates, viral mRNA was translated, protein synthesis visualised by autoradiography and quantified (now Fig. 5d). Both Mallard WT and F313Y showed equivalent levels of translation of viral polypeptides between A549-Empty and A549-BTN3A3 cells (Fig. 5e), suggesting that viral primary transcription is not inhibited by BTN3A3. Moreover, we also performed our PCR assays (distinguishing vRNA, cRNA and mRNA) in the presence and absence of CHX and showed that major differences are only seen for vRNA and cRNA whilst differences in mRNA levels are only seen at later stages post infection. Hence, we can more convincingly conclude that the block appears to be mainly at the level of virus genome replication as initially suggested by the reviewer.

We have revised accordingly the text in the summary and in the text associated with the description of Fig. 5 (Pag. 8-9).

3. The notion that BTN3A3 can restrict influenza viruses in vivo is not supported sufficiently well by the data presented in figure 4g. The experiment in which mice were transduced with BTN3A3-expressing lentivirus shows that only two of five animals had reduced viral titers in the lungs on day 3 post-infection with the sensitive virus. Obviously, a larger number of animals should be tested to strengthen this important claim.

As requested by the reviewer, we have repeated the *in vivo* experiments and now we have higher numbers of individual mice (n=12-14) for each experimental group. This provides more statistical power to the observed differences. We have adjusted Fig. 4h which now includes the new data, as well as the statistical analyses as suggested by the reviewer.

4. It would be of interest to know whether the BTN3A3 is polymorphic and whether patients suffering from accidental infection with avian H5N1 or H7N9 flu viruses carry altered BTN3A3 alleles. Available sequencing data deposited in public libraries should be analyzed.

We added the GWAS data in Extended Data. Unlike Mx1, there are no BTN3A3 SNPs associated with avian IAVs infections. We inserted this point in the Discussion (Pag. 15, lines 4-7) and presented the results in Extended Data, Fig. S15.

"Interestingly, a correlation in human patients between disease severity induced by H7N9 and certain human MX1 alleles has also been described in GWAS studies57. Incidentally, BTN3A3 is very conserved in humans (Extended data Table S4) and no specific association with severe disease and BTN3A3 alleles were found using the same GWAS analyses (Extended data Fig. S15)."

Minor issues:

- Data shown in suppl. figs 1a and 1b are not included well in the story. What is the reason for the high IFN resistance of the mallard virus? Further, the legend says that IFN-λ was used for the experiment shown on the right hand panel of figure 1a. However, the panel says it was IFN-γ. What is correct?

We value the reviewer's suggestions and agree that Fig. S1A and B are indeed not needed. Therefore, given the high number of extended data figures already present, this figure was deleted.

- The true number of ISGs tested in this study is difficult to reconcile from the illustration shown in Figure 1a. The drawing suggests that 871 (527+344) ISGs were tested. However, according to the text above the drawing a total of 752 ISGs were analyzed. What is correct?

We thank the reviewer for pointing this out. We have recounted our numbers of tested ISGs and amended both Fig. 1a and any associated text. With some ISGs, multiple cDNAs were tested, making the number of ISGs and the number of wells in the screen plates not equal. For transparency, we now provide the entirety of the screen data in Extended Data Table S1.

- Some of the viruses listed in Fig. 2e are not grouped correctly: CHIKV is a positive-strand virus, whereas VSV is a negative-strand virus.

As in the previous comment, we thank the reviewer for pointing this out. These 2 viruses have now been correctly placed in Fig. 2e.

- Line 376: reference 27 is not suitable to back up this statement.

We have added an appropriate reference as suggested.

- Material and Methods section. Please describe the characteristics of the siRNAs targeting Mx1, as well as BTN3A3 (1-4), or provide references.

We have now provided the required information in materials and methods section "siRNA-mediated gene silencing" (Page 19, line 18).

- Text page 7, lines 25 and 26. The statement „Avian-to-human mutation F313Y....... exhibits increased Mx1 sensitivity" is wrong. The opposite is true.

We thank the reviewer for spotting this. We have now fixed the sentence.

- The Mx nomenclature is not consistent throughout the text and figures (Mx1, Mx-1). Please adhere to the rules.

We apologise and we have now used the standard nomenclature throughout the text.

Referee #3 (Remarks to the Author):

We thank the reviewer for the supportive statements *…A convincing and well-controlled series of experiments validates the ability of BTN3A3, and to a lesser extent BTN3A1, to block infection and replication by avian influenza isolates, but not human influenza isolates or any of the 24 diverse viruses they tested. …Evolutionary analysis also mapped resistance to BTN3A3 to a specific residue in the viral nucleoprotein (NP) in circulating human influenza isolates. Interestingly, a distinct escape variant is present in H7N9 viruses that have spilled over to humans, demonstrating convergent evolution. These results highlight the novelty of the authors' discovery of BTN3A3 as a restriction factor and the importance of evading BTN3A proteins during cross-species transmission.*

The reviewer provides some constructive criticism and ask for additional experiments and controls.

1. AAV vectors are used to deliver BTN3A3 to the lungs of mice to test its roles in vivo. The authors achieve impressive transduction of these animals. Despite high levels of BTN3A3 expression, only 2 of 5 animals demonstrate reduced viral titers when infected with susceptible virus. The remaining 3 animals showed titers indistinguishable from resistant virus. Moreover, these results are significant when the correct comparisons are made. It would appear more

relevant to compare titers from resistant virus (Cal04) to sensitive virus (Cal04 V313F) in BTN3A3 transduced mice, as this directly addresses the strain-specific inhibitory activity of BTN3A3.

We have repeated the experiments as requested by Reviewer # 3 and # 2 (see response to reviewer # 2 point 3).

2. The mechanism by which BTN3A3 inhibits the viral replication machinery, when this occurs during infection, and how strain-specificity is conferred all remain unresolved. The authors show that the early stages of infection (up to nuclear import of vRNPs) are unaffected by BTN3A3, leading them to investigate its effect on vRNP activity. However, they use indirect measures to conclude that BTN3A3 blocks transcription. vRNPs template both transcription and replication. Given that newly replicated genomes can also serve as templates for further transcription, the levels of replication and transcription are intimately linked. The assays deployed by the authors cannot delineate a specific effect on transcription. Further, none of the experiments shed light on how BTN3A3 achieves its antiviral activity.

a. the authors claim a decrease in mRNA levels at early time points post-infection for susceptible virus in BTN3A3-expressing cells, presumably before replication has begun (Fig 5c). However, this reduction is not significant until 4hpi – the only time point where mRNA is significantly lower – and well after genome replication has occurred. If anything, the more pronounced and consistent reduction in cRNAs at all times during infection might point to a defect in replication.

b. Well-developed infection assays exist to selectively test viral transcription in the absence of genome replication or genome replication in the absence of viral transcription. Data along these lines are needed to support claims that BTN3A3 blocks transcription.

The referee is right, indeed the new experiments that we carried out measuring primary transcription and the PCR assays in the presence or absence of CHX suggest that BTN3A3 acts at the level of replication (see detailed response to point 2 by Referee # 2)

c. pDUAL vectors are used to express viral proteins in minireplicon assays. This adds a serious confounding effect. pDUAL expresses mRNA and genomic vRNA. Functional polymerases complexes will thus act not only on the reporter gene of the minireplicon assay, but also on these additional vRNAs to amplify the polymerase and NP itself. This self-amplification can artificially inflate differences between functional and restricted polymerase complexes. Oddly, while the minireplicon assays report differences in polymerase activity (Fig 5b, ED Fig 11a-b), this is not reflected in blotting for viral proteins that might be expected to also show similar patterns if they are expressed from pDUAL (ED Fig 11c). There appears to be a disconnect between these two sources of data and the use of pDUAL vectors adds unnecessary complications.

As requested by the reviewer we have now repeated the experiments (and replicated our previous results) using pcDNA plasmids which are solely expression plasmids and for which protein expression is not influenced by viral polymerase activity. The data now shown in Fig. 5B and Extended Figure S10 are from experiments performed with these new plasmids. The relevant section in materials and methods has been corrected to describe these changes (Pag. 19, Lines 3-8).

d. While BTN3A3 clearly shows preference in restricting avian-style NP, how specificity if conferred is unknown. Immunoprecipitation shows BTN3A3 interacts equally well with sensitive WT Mallard RNPs and resistant Mallard RNP F313Y (ED F12c).

e. While resistance maps to NP, the authors never show that BTN3A3 interacts with NP. Interactions studies in ED Fig12 included all viral proteins.

We carried out immunoprecipitation assays in virus infected cells, which was the most physiological way to do these assays. It's well known that IAV NP is a very "sticky" protein and co-IPs with this protein in transfection experiments could not provide us with a clear answer with regards to a direct interaction with BTN3A3. The reason behind specific antiviral activity of BTN3A3 against avian viruses remains to be determined. It is possible that BTN3A3 binds differently to NP from different viruses resulting in sensitivity or resistance, or that different adaptors in these interactions influence the phenotype. Understanding why BTN3A3 restricts only avian-style NP despite binding to NPs from sensitive and resistant viruses will be a major undertaking that will have to be developed in other papers. As for any other major restriction factor, it will take likely the contribution of several laboratories and several studies to define the mechanisms of action in

detail. We could take the example of Mx1, which was discovered several decades ago and yet some of these questions have not been completely solved.

3. Evolutionary analyses and functional assays identify a collection of primate paralogs with and without antiviral activity. Yet, the evolutionary difference(s) that confer the antiviral phenotype are not identified. This is particularly intriguing as their phylogeny makes it appear that this activity might have arisen 4-5 independent times.

This is a question we have asked ourselves during the development of this study. However, there are no immediate conserved features that we can identify between primates BTN3A3 with or without restriction activity against avian IAVs (we have added in Annex I an alignment for the Reviewer's perusal). While this is an interesting aspect of BTN3A3 restriction, it is probably secondary to the many aspects that we have highlighted in the manuscript and it will need to be developed in another study.

4. Control blots verifying equivalent protein expression are missing in several experiments. In some cases, these are essential to confirm the protein is actually expressed when the authors report that a paralog does not have antiviral activity. See Fig 3b, 3c, ED Fig 4d, Fig 4e, Fig 6a.

Western blots to check protein expression for Fig 3b have been performed using anti human BTN3A1-3 and are now the new Fig 3c. Regarding Fig 4e, western blotting of Mx1 and BTN3A3 from the used stable cell lines are now added to the new Fig 4. We considered the reference of the reviewer to Fig 6a to be a typo as Fig. 6A refers to viral titres acquired by plaque assays. We also performed westerns controlling minireplicon assays presented now in Fig 5c and Fig. S10 and controlled for NP, BTN3A3, PA, PB1, PB2 and α-Tubulin as loading control.

Fig 3c in the original submission has now been moved to Extended Data Fig S3. Protein expression of the constructs tested in Extended Data Fig. S3 was not possible using commercially available antibodies due to their very high genetic divergency compared to human BTN3A1-3. Therefore, we attempted to tag these BTN genes by the introduction of a C-terminal FLAG. Despite their protein expression being successfully detected using an anti-FLAG antibody, the addition of FLAG to human BTN3A3 used as a control abolished its antiviral activity and therefore this strategy could not be used.
The experiments carried out shown in Extended Data Fig. 4 are largely confirmatory. The antiviral activity of BTN3A3 has evolved after the divergence of old and new world monkeys. Indeed this paralog evolved through duplication events in primates.
We tested all the human paralogues and the BNTN3 orthologues in species infected by IAVs and confirmed that none of the other animal species possess antiviral activity. All these genes were cloned exactly in the same way as the human BTN3A3 in the same lentivirus vector and none of them showed any antiviral activity. The lentivirus vector expresses the RFP fluorophore and data are obtained in transduced cells only. None of the genes, as unsurprising given the sequence divergence, displayed any antiviral activity. Nonetheless, we have added in the Legend of Extended Fig. S3 that protein expression could not be verified by western blotting in these specific experiments.

Minor:

1. The authors nicely show that the antiviral activity of BTN3A3 and MX1 function independently of each other, even if they both act on the same sites of NP. Nonetheless, caution is warranted in the discussion of viral evolution, as evolutionary pressure causing changes at NP 52/313 are likely some combination of BTN3A3 and MX1.

We agree, this is a good point. We have modified slightly the discussion to take on board the comments of the reviewer as follows (Pag. 15, lines 13-18):

"It is however relevant that two independent human restriction factors target a conserved surface of NP, suggesting that this constrained region is a key determinant of the zoonotic potential of avian IAV. Hence, Mx1 and BTN3A3 could have independent but synergistic effects on blocking avian IAV replication and may apply evolutionary pressures in similar areas of NP."

2. Fig 6h models structural differences for various avian NPs. Modelling is not necessary for 52Q/313F as several structures have been solved for avian NP (e.g. 2Q06, 7DKG, 7DXP. These structures show inherent flexibility of sidechains at this position.

We agree with the reviewer and deleted the panels in question.

3. Page 14 line 25 – please clarify that these data are present in Fig 2e, not 3a.
4. Fig 6 legend lacks notations for panel 6f, which is misassigned at a duplicated 6e.

We thank the reviewer for pointing this out. These errors have been fixed.

5. When referring to NP from 2009 pH1N1, please note how amino acid numbering was assigned. 2009 pH1N1 NP contains an N-terminal addition relative to all other NPs which can confuse numbering when compared to other NPs.

Yes, we are aware of the 6 amino acid N-terminal extension present in NP of pmd09 viruses. However, in order to match most NPs, we considered pmd09-related NP sequences to start from the second methionine codon. This has now been made explicit in the materials and methods section "IAVs phylogenetic analysis", page 25, lines 23-26.

6. Page 11 Line 11 – please clarify, as non-human primates can also be naturally infected with IAV (Karlsson et al. 2012 DOI: 10.3201/eid1809.120214).

We agree with the reviewer. We have modified slightly the sentence as to take on board this reference (Pag. 13, lines 28-31).
Hence, humans are the only species maintaining endemic infections with influenza viruses that restrict avian IAV replication through this effector. Neither avian species nor swine impose a BTN3A3-related selective pressure on the NP 313 residue."

We thank again the reviewers for their constructive criticism and we hope that the manuscript is now ready for publication in Nature.

Sincerely

Massimo Palmarini

Encl Annex I

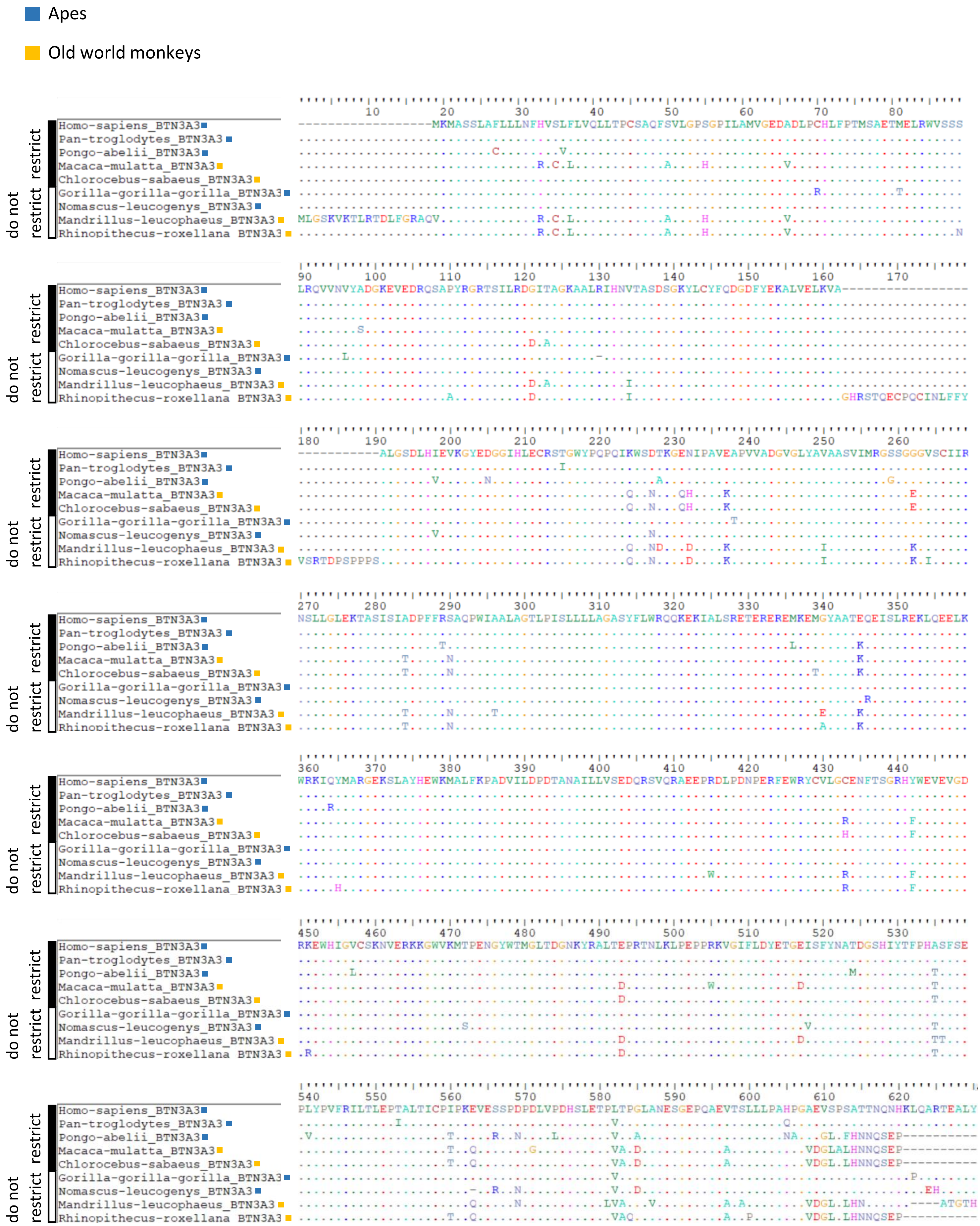

Apes
Old world monkeys
restrict
do not restrict
Homo-sapiens_BTN3A3
Pan-troglodytes_BTN3A3
Pongo-abelii_BTN3A3
Macaca-mulatta_BTN3A3
Chlorocebus-sabaeus_BTN3A3
Gorilla-gorilla-gorilla_BTN3A3
Nomascus-leucogenys_BTN3A3
Mandrillus-leucophaeus_BTN3A3
Rhinopithecus-roxellana_BTN3A3

Reviewer Reports on the First Revision:

Referees' comments:

Referee #2 (Remarks to the Author):

The authors performed all additional studies that I had requested, and the new results were included in the revised version of this manuscript. The story is now truly fascinating.

Referee #3 (Remarks to the Author):

The authors provided a comprehensive and compelling response to reviews; the experimental attention to detail and thoroughness are appreciated.

A major concern was the in vivo phenotype. The revision includes more animals, increasing the power of the analyses and providing stronger evidence that BTN3A3 restricts influenza virus in vivo, dependent on the identify of NP aa313.

The authors have also identified a major step in replication that is blocked by BTN3A3. Primary transcription was assessed with a functional in vitro translation assay. These data indicate the BTN3A3 does not impact transcription from incoming RNPs. Strand-specific RT-qPCR then confirmed that the primary effect is on genome replication. These data identify a discrete step in the viral lifecycle affected by BTN3A3, consistent with the functions of its target NP.

How BTN3A3 differentially impacts NP F313 but not NP Y313 remains unresolved. The authors show that it probably does not occur at the level of interactions between BTN3A3 AND NP. However, exploring this additional aspect is not needed to support the main conclusions of the paper that BTN3A3 is a restriction factors the protects primates from avian IAV infection.

Referee #4 (Remarks to the Author):

I have read the manuscript by Pinto et al and the responses to the comments by previous reviewers. This is an interesting and well-written paper that shines new light on host-pathogen interactions.

While I agree that the description of BTN3A3's role in limiting avian but not human IAVs is interesting, I do feel that it lacks some context regarding work that described the potential impact of these substitutions as much as a decade earlier. These studies lack the very deep characterizations BTN3A3 found in this study, but the relationship between this study and previous studies should be clearly described in the main text.

https://pubmed.ncbi.nlm.nih.gov/36588386/
https://journals.plos.org/plospathogens/article?id=10.1371/journal.ppat.1003279

Other than that, this study represents an impressive amount of work.

Author Rebuttals to First Revision:

We thank all the reviewers for their supportive statements and insightful comments throughout the reviewing process. There is only one outstanding issue highlighted by Reviewer # 4. The reviewer commented "*While I agree that the description of BTN3A3's role in limiting avian but not human IAVs is interesting, I do feel that it lacks some context regarding work that described the potential impact of these substitutions as much as a decade earlier. These studies lack the very deep characterizations BTN3A3 found in this study, but the relationship between this study and previous studies should be clearly described in the main text*".

The reviewer suggested the following publication

https://journals.plos.org/plospathogens/article?id=10.1371/journal.ppat.1003279 (Manz et al, 2013) describing how influenza virus escapes restriction by human MxA through mutations in NP. This is an important manuscript as it shows that there are different "patches" in NP that affect the ability of human Mx1 to preferentially block avian IAVs compared to mammalian IAVs.

We feel that we had already placed our study in the context of the study by Manz et al. (and the one from another group published in 2015 – reference 19). First, we had already referred to this this study both in the Results and Discussion sections. In the results (resubmitted manuscript page 7, lines 6-7) we state "**Interestingly, residue 313 had been previously associated with evasion of Mx1 resistance**[18,19]." And it is exactly for this reason that we embarked in a series of experiments to show that BTN3A3 acts independently from Mx1 (Fig. 3e-f; Extended Data Fig. 7a-b). In the Discussion (page 13, line 32 - page 14, line 11), we reiterated this point further and referred again to the papers above; "**Mx1 is another restriction factor for avian IAVs. The NP residues 313 and 52 which are critical BTN3A3 evasion/resistance have been associated also with Mx1 resistance**[18,19]**. We presented in vitro data suggesting that, despite affecting similar stages of the virus life cycle and through common amino acid residues, BTN3A3 acts independently from Mx1. Moreover, our in vivo experiments were carried out in regular C57BL/6 which, like many other laboratory mouse strains, possess a non-functional Mx1**[48,49]**, further reinforcing the point that BTN3A3 acts in an Mx1-independent manner. It is however relevant that two independent human restriction factors target a conserved surface of NP, suggesting that this constrained region is a key determinant of the zoonotic potential of avian IAV**".

We did notice however that in our Supplementary Discussion, where we discuss the "Molecular dating of the F313V NP change", we had not referred to the paper by Manz et al, but we have now introduced this reference and highlighted that these residues had been described in the context of Mx1 sensitivity.

The referee also highlights a recent publication (https://pubmed.ncbi.nlm.nih.gov/36588386/) (Zhang et al, 2023) published after our original submission suggesting that the nucleoprotein of influenza A virus inhibits the innate immune response by inducing mitophagy and the Y313F mutation affects negatively this process._ We have now added a reference to this paper in the Discussion (Pag 14, line 7) **"It is also interesting to note a recent study suggesting that IAV NP regulates mitophagy and the Y313F mutation attenuates this process49. Hence, at least two independent human restriction**

factors target a conserved surface of NP, suggesting that this constrained region is a key determinant of the zoonotic potential of avian IAV”.
