# Supplementary material for: BTN3A3 evasion promotes the zoonotic potential of influenza A viruses
Source: Nature. Author manuscript; Available in PMC 2026 Oct 15. (PMC7619554; doi:10.1038/s41586-023-06261-8)
Supplement: Supplementary file 2 [file EMS219322-supplement-Reporting_Summary.pdf]

nature portfolio

Corresponding author(s): Massimo Palmarini

Last updated by author(s): May 5, 2023

Reporting Summary

Nature Portfolio wishes to improve the reproducibility of the work that we publish. This form provides structure for consistency and transparency in reporting. For further information on Nature Portfolio policies, see our Editorial Policies and the Editorial Policy Checklist.

Statistics

For all statistical analyses, confirm that the following items are present in the figure legend, table legend, main text, or Methods section.

n/a	Confirmed	
☐	☒	The exact sample size (*n*) for each experimental group/condition, given as a discrete number and unit of measurement
☐	☒	A statement on whether measurements were taken from distinct samples or whether the same sample was measured repeatedly
☐	☒	The statistical test(s) used AND whether they are one- or two-sided<br>*Only common tests should be described solely by name; describe more complex techniques in the Methods section.*
☒	☐	A description of all covariates tested
☐	☒	A description of any assumptions or corrections, such as tests of normality and adjustment for multiple comparisons
☐	☒	A full description of the statistical parameters including central tendency (e.g. means) or other basic estimates (e.g. regression coefficient) AND variation (e.g. standard deviation) or associated estimates of uncertainty (e.g. confidence intervals)
☐	☒	For null hypothesis testing, the test statistic (e.g. *F*, *t*, *r*) with confidence intervals, effect sizes, degrees of freedom and *P* value noted<br>*Give P values as exact values whenever suitable.*
☐	☒	For Bayesian analysis, information on the choice of priors and Markov chain Monte Carlo settings
☒	☐	For hierarchical and complex designs, identification of the appropriate level for tests and full reporting of outcomes
☒	☐	Estimates of effect sizes (e.g. Cohen's *d*, Pearson's *r*), indicating how they were calculated

Our web collection on statistics for biologists contains articles on many of the points above.

Software and code

Policy information about availability of computer code

Data collection	Sequences of BTN paralogues/homologues were sourced from NCBI and/or ensemble. IDs are provided in Supplementary Table 5.<br>NP coding sequences unique on the nucleotide level (identical sequences collapsed) were retrieved from the NCBI Flu database (https://www.ncbi.nlm.nih.gov/genomes/FLU/Database/nph-select.cgi?go=database, as of the 8th of June 2021, sampled until the end of 2020).<br>Moreover, during the revision, influeza A segment 5 sequences were also retrieved from GISAID EpiFlu (Supplementary Table s2-4).<br>Acknowledgments for each avian IAV human and bird isolates used in this study are stated in Supplementary Tables 7-8.
Data analysis	Data visualisation and statistical analysis: GraphPad Prism 8<br>Immunofluorescence imaging acquisition: Zen Microscopy Software (Zeiss)<br>Cytoplasmic/nuclear fluorescent intensity quantification: Cell Profiler<br>Sequence homology search: Blast v.2.8.1<br>HMM profile protein domain search: HMMER v.3.3<br>Sequence alignment: Mafft v.7.453<br>Maximum likelihood phylogenetic inference: iqtree v.1.6.12<br>Sequence alignment manipulation: pal2nal v.14<br>Sequence clustering: MMseqs2 v.13.45111<br>Phylogeny time calibration: TreeTime v.0.9.0<br>Coding languages: Python v.3.8.5, R v.4.1.3<br>Phylogeny summary and manipulation: ete3 v.3.1.2<br>Sequence data summary and manipulation: biopython v.1.76<br>Tabular information summary and manipulation: pandas v.1.2.4

Phylogeny visualisation: ggtree v.3.2.1, FigTree v.1.4
Bayesian phylogenetic inference: BEAST v.1.10.4, BEAUti v.1.10.4, LogCombiner v.1.10.4

For manuscripts utilizing custom algorithms or software that are central to the research but not yet described in published literature, software must be made available to editors and reviewers. We strongly encourage code deposition in a community repository (e.g. GitHub). See the Nature Portfolio guidelines for submitting code & software for further information.

Data

Policy information about availability of data

All manuscripts must include a data availability statement. This statement should provide the following information, where applicable:

- Accession codes, unique identifiers, or web links for publicly available datasets
- A description of any restrictions on data availability
- For clinical datasets or third party data, please ensure that the statement adheres to our policy

Alignments and raw phylogenetic data related to this study can be found in the following GitHub repository: https://github.com/spyros-lytras/BTN3A3_IAV. Source data related to the animal experiments illustrated in Fig. 3h are available in Enlighten Research Data at the following link: http://dx.doi.org/10.5525/gla.researchdata.1425. Gel source data are available in Supplementary Figure 1.

Human research participants

Policy information about studies involving human research participants and Sex and Gender in Research.

Reporting on sex and gender	*Use the terms sex (biological attribute) and gender (shaped by social and cultural circumstances) carefully in order to avoid confusing both terms. Indicate if findings apply to only one sex or gender; describe whether sex and gender were considered in study design whether sex and/or gender was determined based on self-reporting or assigned and methods used. Provide in the source data disaggregated sex and gender data where this information has been collected, and consent has been obtained for sharing of individual-level data; provide overall numbers in this Reporting Summary. Please state if this information has not been collected. Report sex- and gender-based analyses where performed, justify reasons for lack of sex- and gender-based analysis.*
Population characteristics	*Describe the covariate-relevant population characteristics of the human research participants (e.g. age, genotypic information, past and current diagnosis and treatment categories). If you filled out the behavioural & social sciences study design questions and have nothing to add here, write "See above."*
Recruitment	*Describe how participants were recruited. Outline any potential self-selection bias or other biases that may be present and how these are likely to impact results.*
Ethics oversight	*Identify the organization(s) that approved the study protocol.*

Note that full information on the approval of the study protocol must also be provided in the manuscript.

Field-specific reporting

Please select the one below that is the best fit for your research. If you are not sure, read the appropriate sections before making your selection.

☒ Life sciences ☐ Behavioural & social sciences ☐ Ecological, evolutionary & environmental sciences

For a reference copy of the document with all sections, see nature.com/documents/nr-reporting-summary-flat.pdf

Life sciences study design

All studies must disclose on these points even when the disclosure is negative.

Sample size	For experiments involving quantification of BTN3A3 and NP from immunofluorescence samples, more than 3500 cells were analysed from 4 independent experiments (Figure 5d-f and Extended data figure 13).<br>For the animal experiments, group sizes were chosen on the bases of a pilot experiment to assess AAV transduction and previous experience.
Data exclusions	Lung titres from mice which intranasal infection was not successful were discarded from the analysis (Figure 4g). No other data was excluded.
Replication	For experiments involving quantification of BTN3A3 and NP from immunofluorescence samples, biological variability was observed between experiments. All data from the 4 independent experiments was combined and presented. All replication attempts for the remaining experiments were successful and presented in the respective figures.
Randomization	Fields of infected of mock-infected cells were selected randomly for imaging purposes. For the animal experiments No specific randomisation were conducted. Analysis of data was conducted in an unbiased manner but no specific blinding was used for the researchers carrying out virus titration from mice tissues.

Blinding	Blinding was not performed.

Reporting for specific materials, systems and methods

We require information from authors about some types of materials, experimental systems and methods used in many studies. Here, indicate whether each material, system or method listed is relevant to your study. If you are not sure if a list item applies to your research, read the appropriate section before selecting a response.

Materials & experimental systems

n/a	Involved in the study
☐	☒ Antibodies
☐	☒ Eukaryotic cell lines
☒	☐ Palaeontology and archaeology
☐	☒ Animals and other organisms
☒	☐ Clinical data
☒	☐ Dual use research of concern

Methods

n/a	Involved in the study
☒	☐ ChIP-seq
☐	☒ Flow cytometry
☒	☐ MRI-based neuroimaging

Antibodies

Antibodies used	Primary antibodies:<br>Rabbit anti-NP polyclonal - Novus Biologicals (NBP2-16965) - used for immunoprecipitation (IP - 3µg/mg of protein lysate) and western-blot (WB - 1:1000)<br>Mouse anti-NP monoclonal C43 - AbCam (ab128193) - used for WB (1:1000)<br>Mouse anti-NP monoclonal AA5H - AbCam (ab20343) - used Immunofluorescence (IF) (1:2000)<br>Rabbit anti-MBP-NP polyclonal (antiserum 2915) - Custom made - used WB (1:500)<br>Rabbit anti-BTN3A3 polyclonal - Atlas Antibodies (HPA007904) - used for IP (3µg/mg of protein lysate), IF (1:200) and immunohistochemistry (IHC) (1:250)<br>Rabbit anti-BTN3A3 polyclonal - Proteintech (15896-1-AP) - used for WB (1:750)<br>Mouse anti-BTN3A3 monoclonal 1A3B5 - Proteintech (67560-1-Ig) used for WB (1:1000)<br>Rabbit anti-PA polyclonal - Genetex (GTX118991) - used for WB (1:1000)<br>Rabbit anti-PB1 polyclonal - Thermo Fisher (PA5-34914) - used for WB (1:1000)<br>Rabbit anti-PB2 polyclonal - Genetex (GTX125926) - used for WB (1:1000)<br>Rabbit anti-Histone H3 polyclonal - AbCam (ab1791) - used for WB (1:1000)<br>Rabbit anti-GAPDH monoclonal 14C10 - Cell Signaling Technology (2118S) used for WB (1:10000)<br>Mouse anti-alpha-Tubulin monoclonal DM1A - Sigma-Aldrich (T6199) used for WB (1:5000)<br>Rabbit anti-GFP - Cell Signaling (2555s) used for IHC (1:1500)<br>Rabbit anti-RSAD2 monoclonal (D5T2X) - Cell Signaling (13996S) used 1:1000<br><br>Isotype controls:<br>Rabbit IgG isotype control - Thermo Fisher (02-6102) - used for IP (3µg/mg of protein lysate)<br>Rabbit serum - Sigma-Aldrich (R4505) - used for IHC (1:250 or 1:1500)<br><br>Secondary antibodies:<br>Anti-rabbit IgG (H+L) (DyLight™ 800 4X PEG Conjugate) - Cell Signaling Technology (5151S) - used for WB (1:10000)<br>Anti-mouse IgG (H+L) (DyLight™ 680 Conjugate) - Cell Signaling Technology (5470S) - used for WB (1:10000)<br>Goat anti-Mouse IgG (H+L) Cross-Adsorbed Secondary Antibody, Alexa Fluor™ 555 - Thermo Fisher (A-21422) - used for IF (1:2000)<br>Goat anti-Rabbit IgG (H+L) Cross-Adsorbed Secondary Antibody, Alexa Fluor™ 488 - Thermo Fisher (A-11008) - used for IF (1:400)
Validation	NBP2-16965: Validation showed by the manufacturer for several techniques (western blot, immunohistochemistry, immunofluorescence, immunoprecipitation and sandwich ELISA). Validated by comparison of mock-infected and infected cells lysates. Migrates at the expected molecular weight.<br>ab128193: Validation showed by the manufacturer for several techniques (western blot, ELISA, immunofluorescence and flow cytometry). It's has been cited 29 times.<br>ab20343: Validation showed by the manufacturer for several techniques (immunofluorescence and immunohistochemistry). It's has been cited 82 times.<br>Custom made rabbit MBP-NP (2915) primary antibody: Validated by comparison of mock-infected and infected cells lysates. Migrates at the expected molecular weight (Digard et al, 1999 - DOI: 10.1128/JVI.73.3.2222-2231.1999)<br>HPA007904: Validation showed by the manufacturer for several techniques (immunohistochemistry and western blot). Immunohistochemistry validation was performed by Orthogonal validation of protein expression using IHC by comparison to RNA-seq data of corresponding target in high and low expression tissues.<br>15896-1-AP: Validation showed by the manufacturer for several techniques (western-blot, immunoprecipitation and immunofluorescence).<br>67560-1-Ig: Validation showed by the manufacturer for western-blot.<br>GTX118991: Validation showed by the manufacturer for several techniques (western-blot and immunofluorescence). Validated by comparison of mock-infected and infected cells lysates. Migrates at the expected molecular weight. Cited 40 times.<br>PA5-34914: Validation showed by the manufacturer for several techniques (western-blot, immunofluorescence and CUT&RUN). Cited 4 times.<br>GTX125926: Validation showed by the manufacturer for western-blot and immunofluorescence. Cited 49 times.

ab1791: Validation showed by the manufacturer for several techniques (western blot, immunocytochemistry, ChIP and Immunoprecipitation). It's has been cited more than 3000 times.
2118S: Validation showed by the manufacturer for several techniques (western blot, immunohistochemistry, immunofluorescence and flow cytometry). It's has been cited more than 5000 times.
T6199: Validation showed by the manufacturer for western blot and immunofluorescence. It's has been cited more than 2000 times.
2555s: Validation showed by the manufacturer for western blot and immunohistochemistry. It's has been cited more than 200 times.
13996S: Validation showed by the manufacturer for western blot. It's has been cited 14 times.

Eukaryotic cell lines

Policy information about cell lines and Sex and Gender in Research

Cell line source(s)	MT4 (gift from Paul Bieniasz, Rockefeller University); Madin-Darby canine kidney (MDCK) cells (ATCC); human embryonic kidney cells (293T) (ATCC); A549 (ATCC); MDCK cells expressing Sialyltransferase 1 (MDCK-SIAT), kindly gifted by John McCauley, The Francis Crick Institute). hTERT-immortalized primary human foetal lung fibroblasts were generated at the CVR. Normal human bronchial epithelial cells immortalised with CDK4 and hTERT (hBEC3-KT) (UT Southwestern Medical Center).
Authentication	MT4 cells and A549 were authenticated using short tandem repeat (STR) analysis carried out by either the DNA Diagnostics Centre (United Kingdom) or Eurofins (United Kingdom), and analysed using the DSMZ online STR analysis tool.
Mycoplasma contamination	Cells were tested for mycoplasma contamination on a bi-monthly basis (average). All experiments were performed in mycoplasma negative cells.
Commonly misidentified lines (See ICLAC register)	No commonly misidentified cell lines were used.

Animals and other research organisms

Policy information about studies involving animals; ARRIVE guidelines recommended for reporting animal research, and Sex and Gender in Research

Laboratory animals	6 week old female C57BL/6 mice
Wild animals	No wild animals were used in this study.
Reporting on sex	Only female mice were used in the study for practical reasons. Male mice fight with each other when housed in the same cage.
Field-collected samples	No field-collected samples were used in this study
Ethics oversight	Animals were maintained at the University of Glasgow under specific pathogen free conditions in accordance with UK home office regulations (Project License PP1902420) and approved by the University of Glasgow ethics committee.

Note that full information on the approval of the study protocol must also be provided in the manuscript.

Flow Cytometry

Plots

Confirm that:

- [x] The axis labels state the marker and fluorochrome used (e.g. CD4-FITC).
- [x] The axis scales are clearly visible. Include numbers along axes only for bottom left plot of group (a 'group' is an analysis of identical markers).
- [x] All plots are contour plots with outliers or pseudocolor plots.
- [ ] A numerical value for number of cells or percentage (with statistics) is provided.

Methodology

Sample preparation	Cell lines were infected with the mentioned GFP-tagged viruses, trypsined and fixed in 4% formaldehyde.
Instrument	Millipore GUAVA easyCyte HT
Software	Flow cytometry data acquisition was performed using InCyte 3.3 and analysed using FlowJo (v10.6.1).
Cell population abundance	Physical cell sorting was not performed. Only percentage of RFP- and GFP-positive cells was quantified.
Gating strategy	Provided in Supplementary Figure 2

- [x] Tick this box to confirm that a figure exemplifying the gating strategy is provided in the Supplementary Information.
