# Supplementary material for: BTN3A3 evasion promotes the zoonotic potential of influenza A viruses
Source: Nature. Author manuscript; Available in PMC 2026 Oct 15. (PMC7619554; doi:10.1038/s41586-023-06261-8)

Supplementary Figure 1

Fig 1c and Fig ED – MRC5T

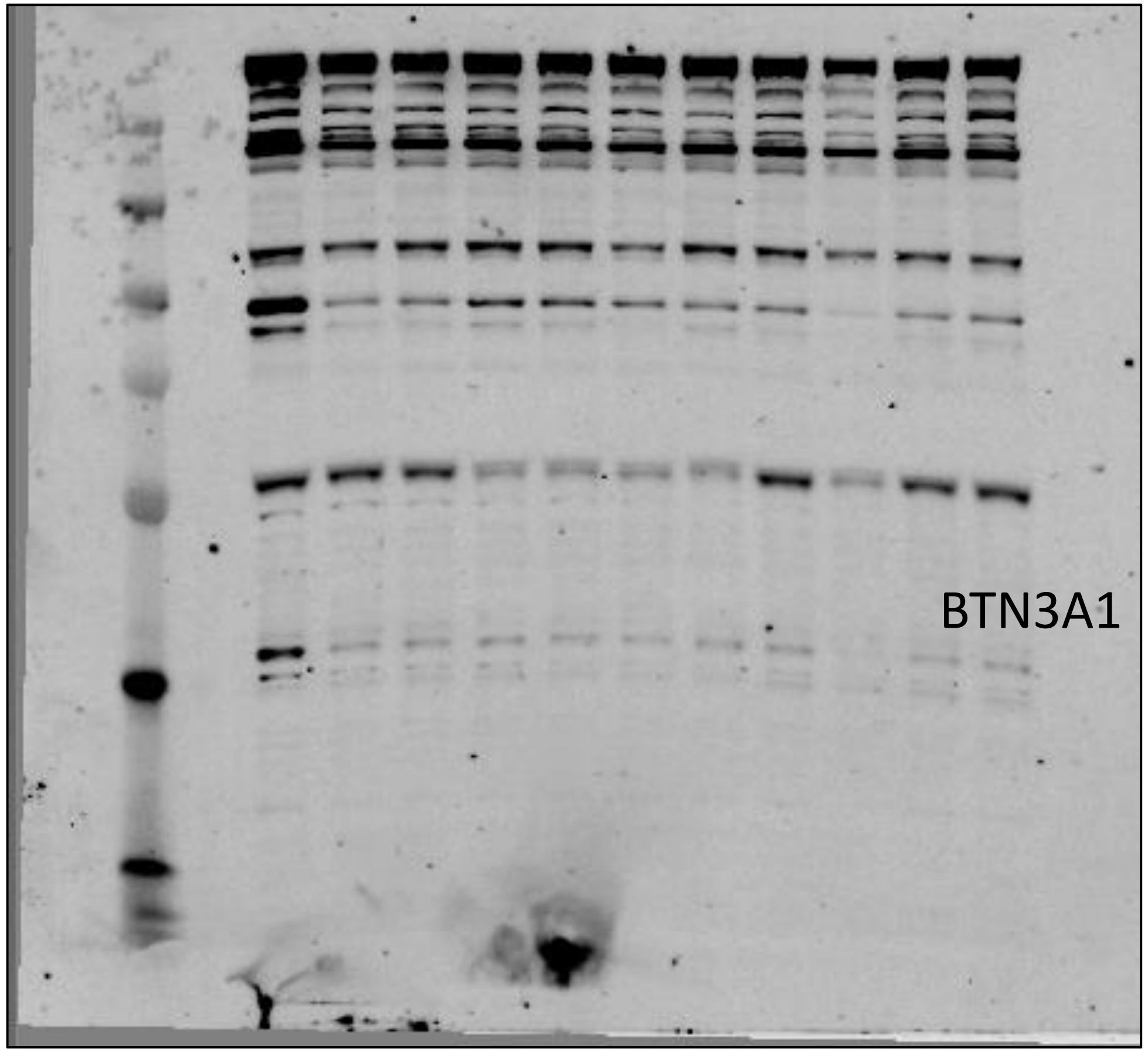

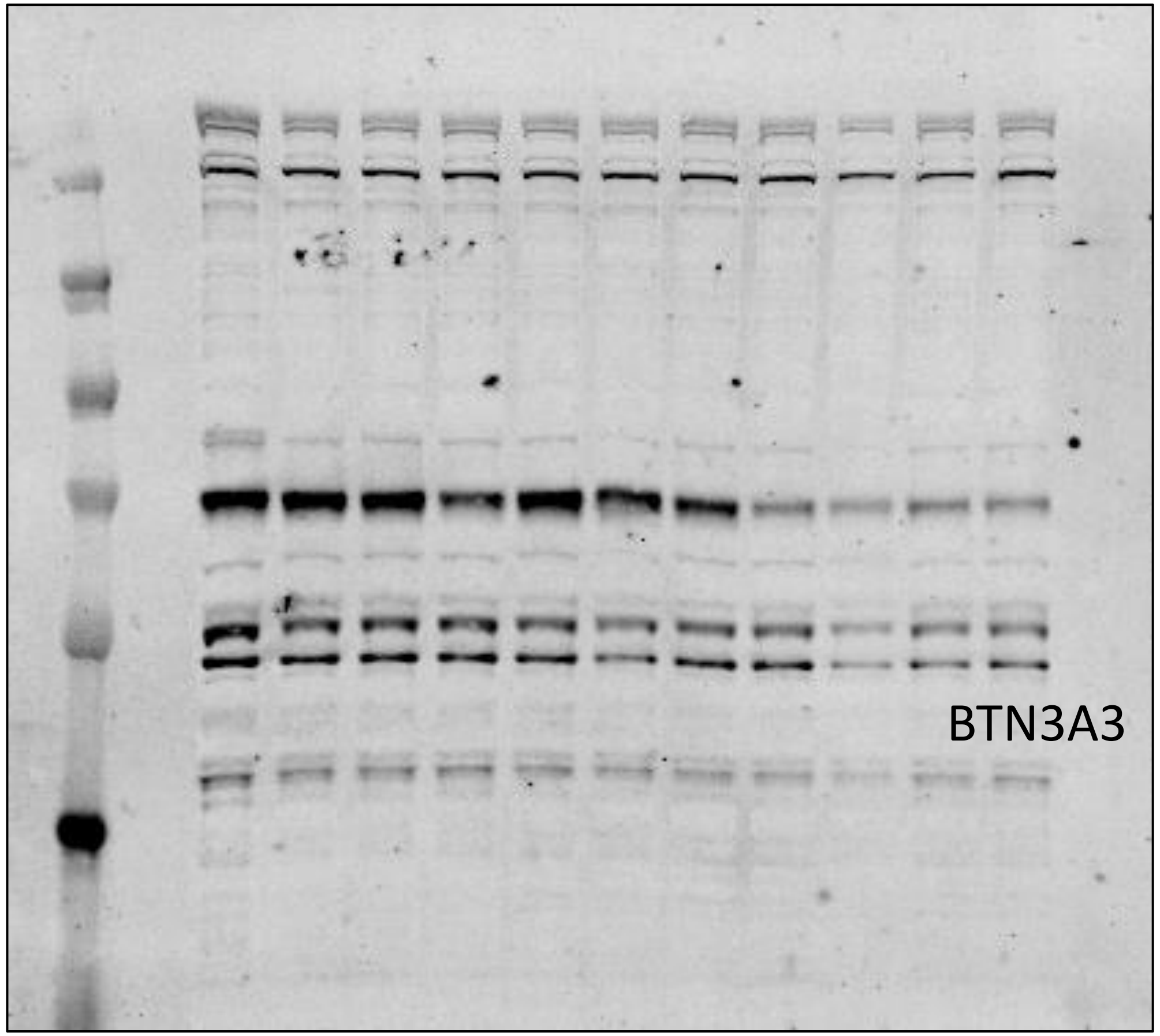

Fig 1c and Fig ED – hBEC3-KT

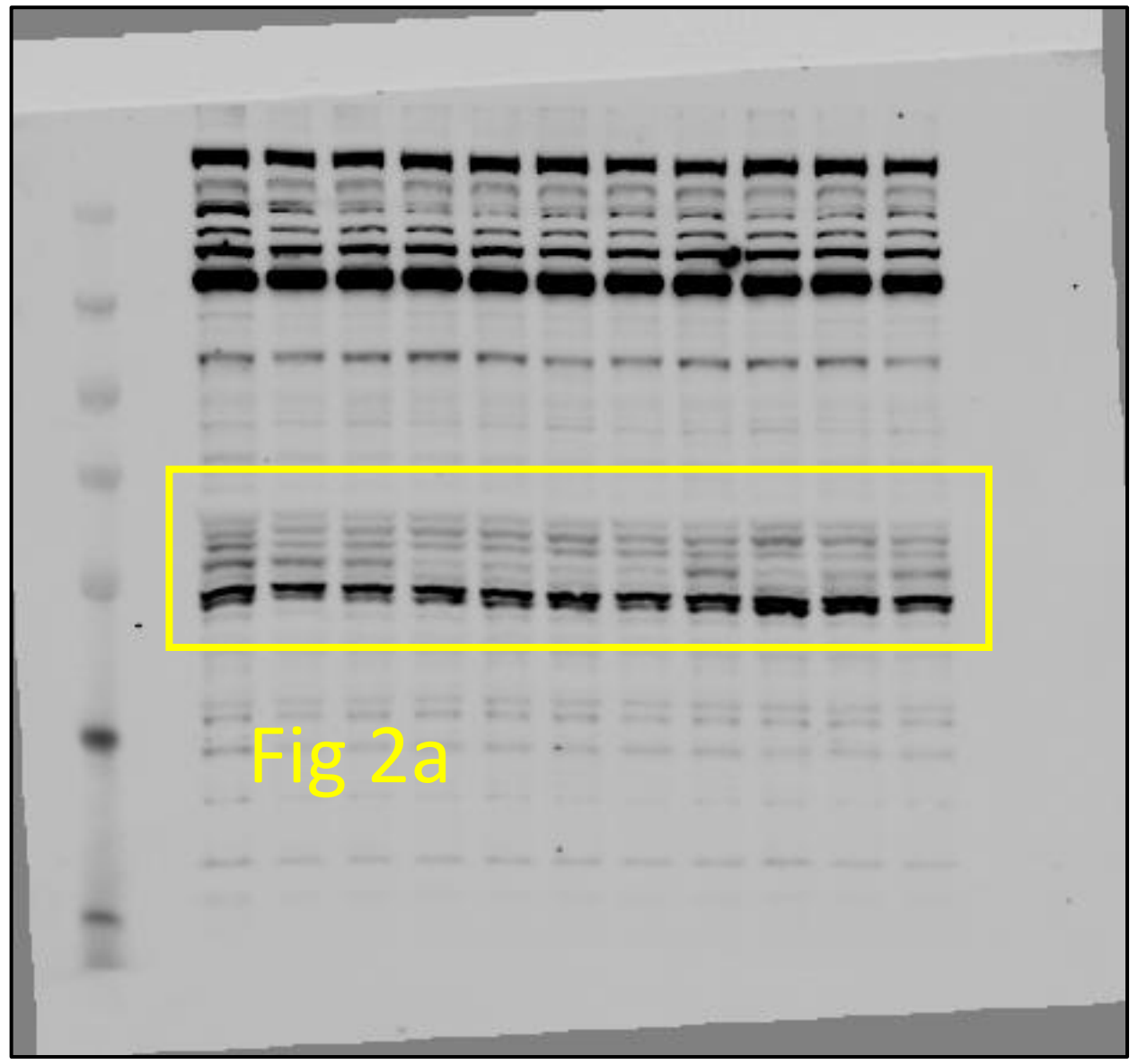

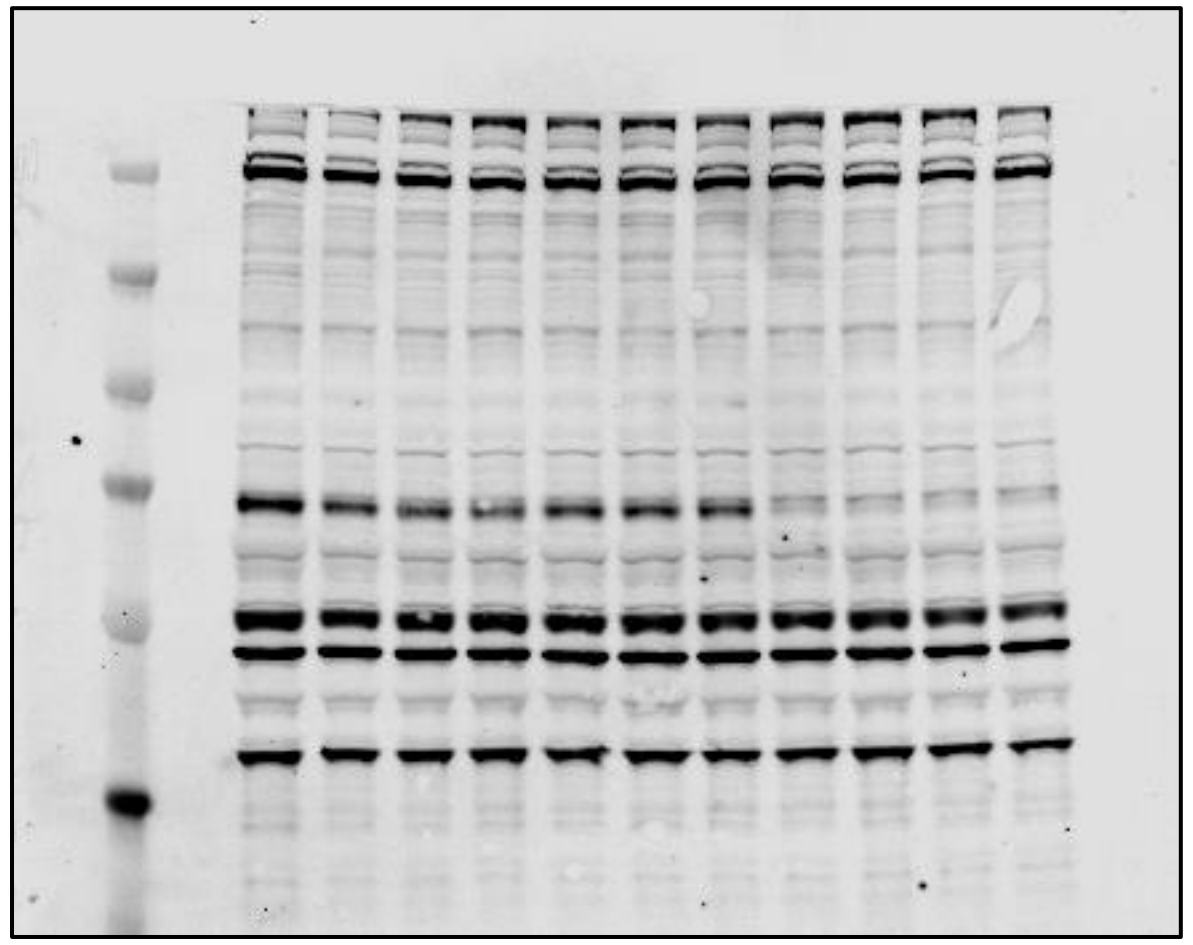

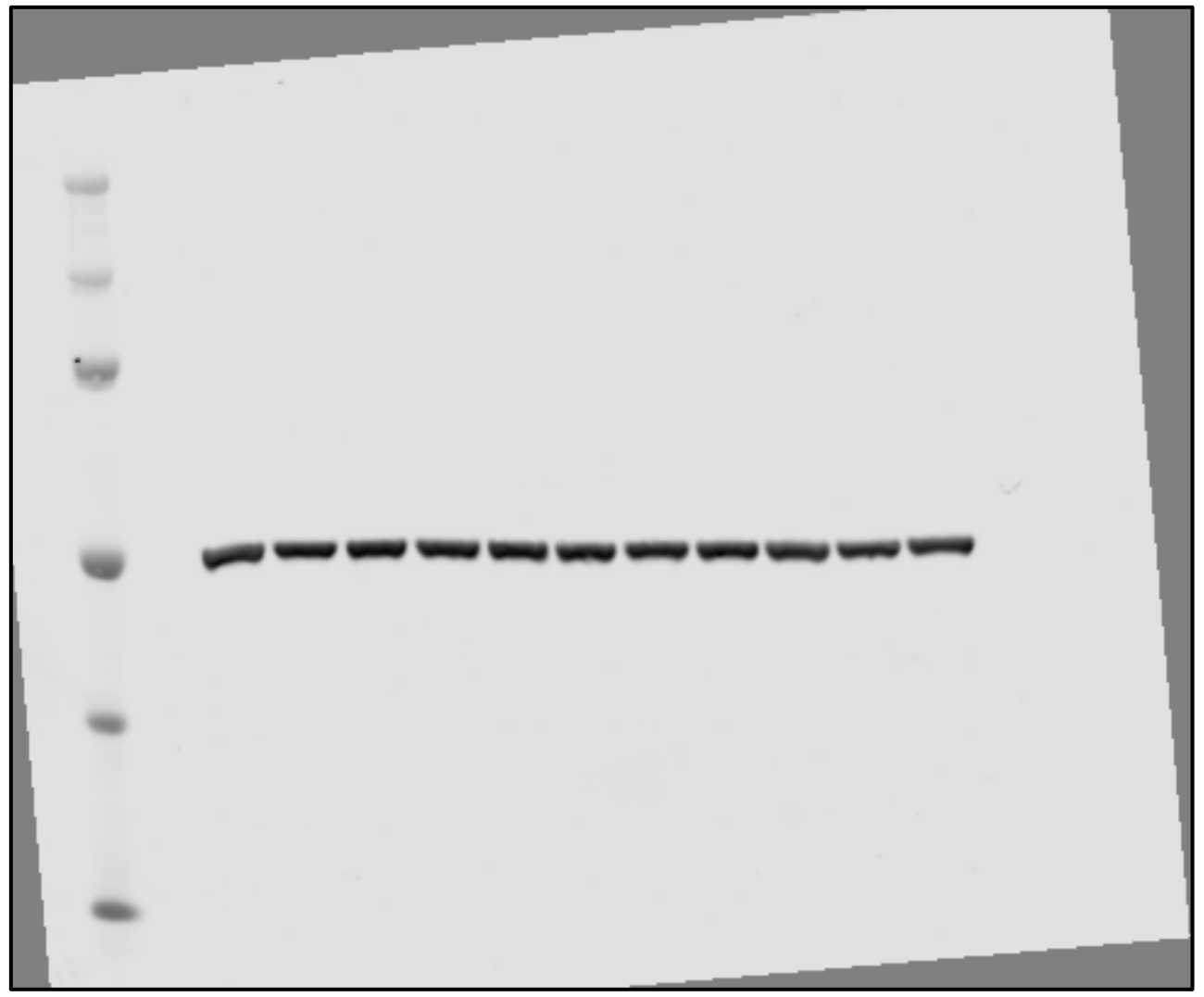

Fig. 2a

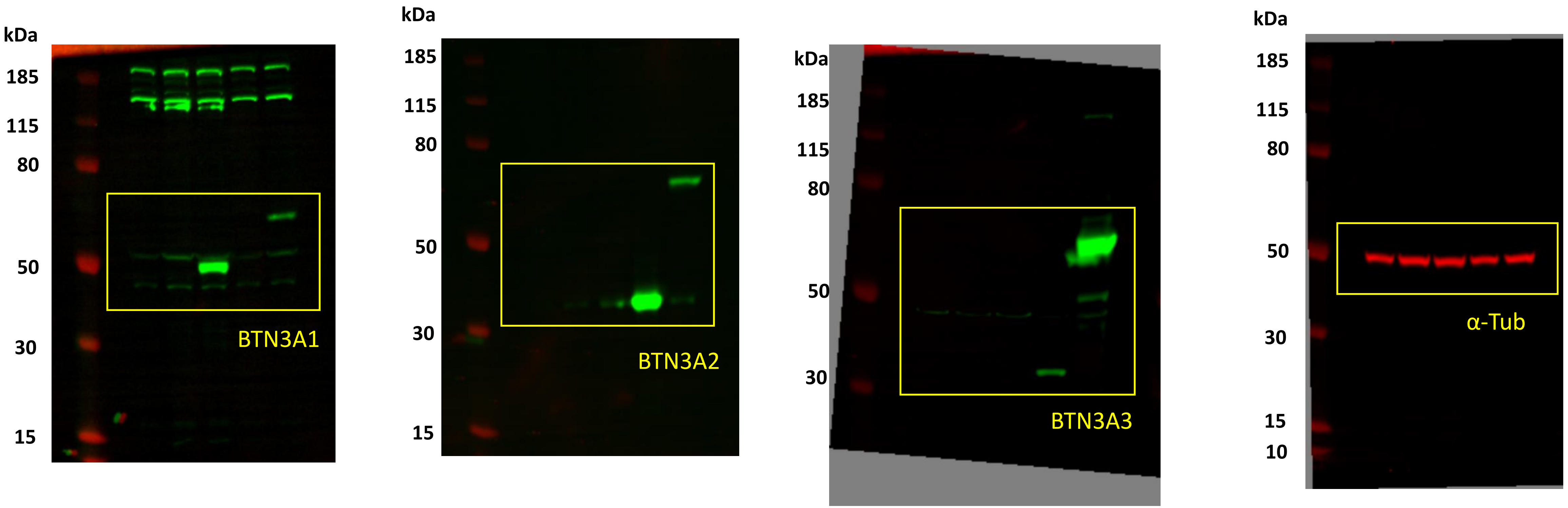
kDa
185
115
80
50
30
15
BTN3A1
kDa
185
115
80
50
30
15
BTN3A2
kDa
185
115
80
50
30
BTN3A3
kDa
185
115
80
50
30
15
10
α-Tub

Fig. 2c – PR8

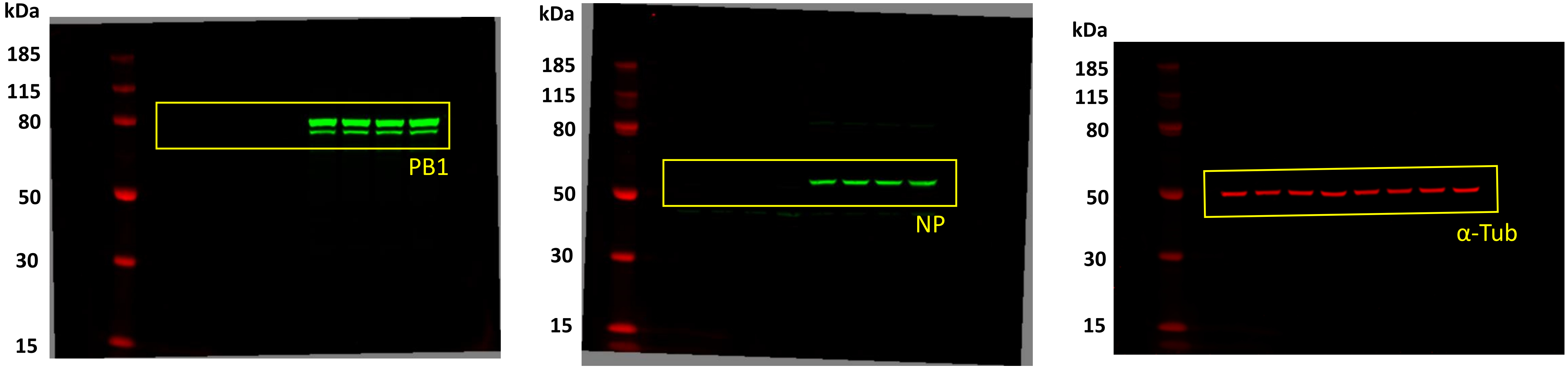
kDa
185
115
80
50
30
15
PB1
kDa
185
115
80
50
30
15
NP
kDa
185
115
80
50
30
15
α-Tub

Fig. 2c – Mallard

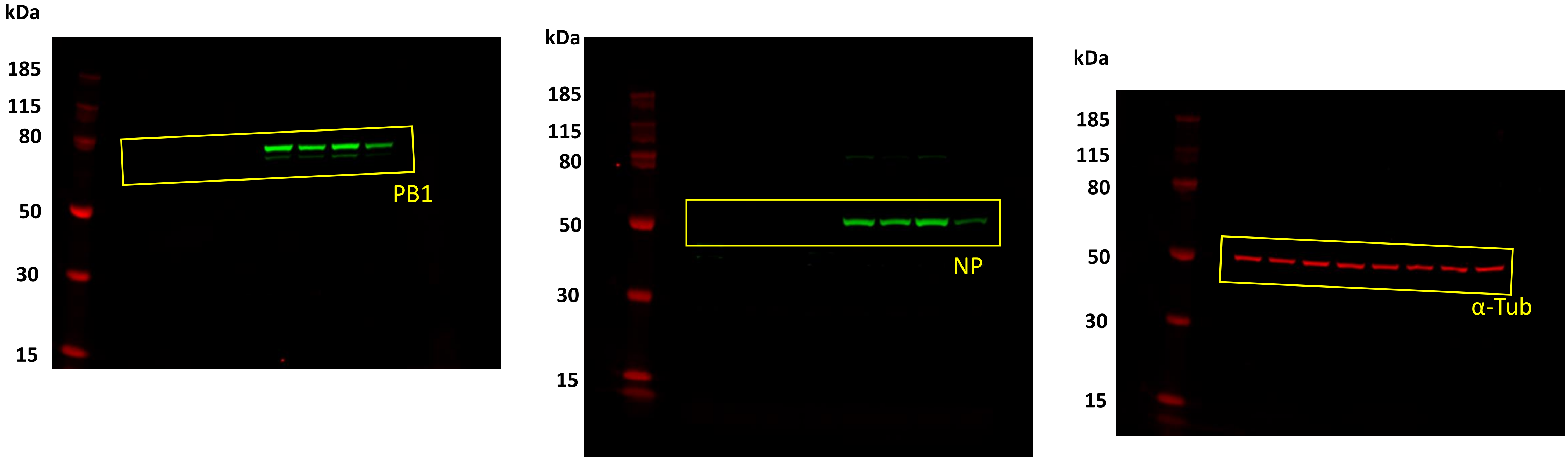

Fig. 3e

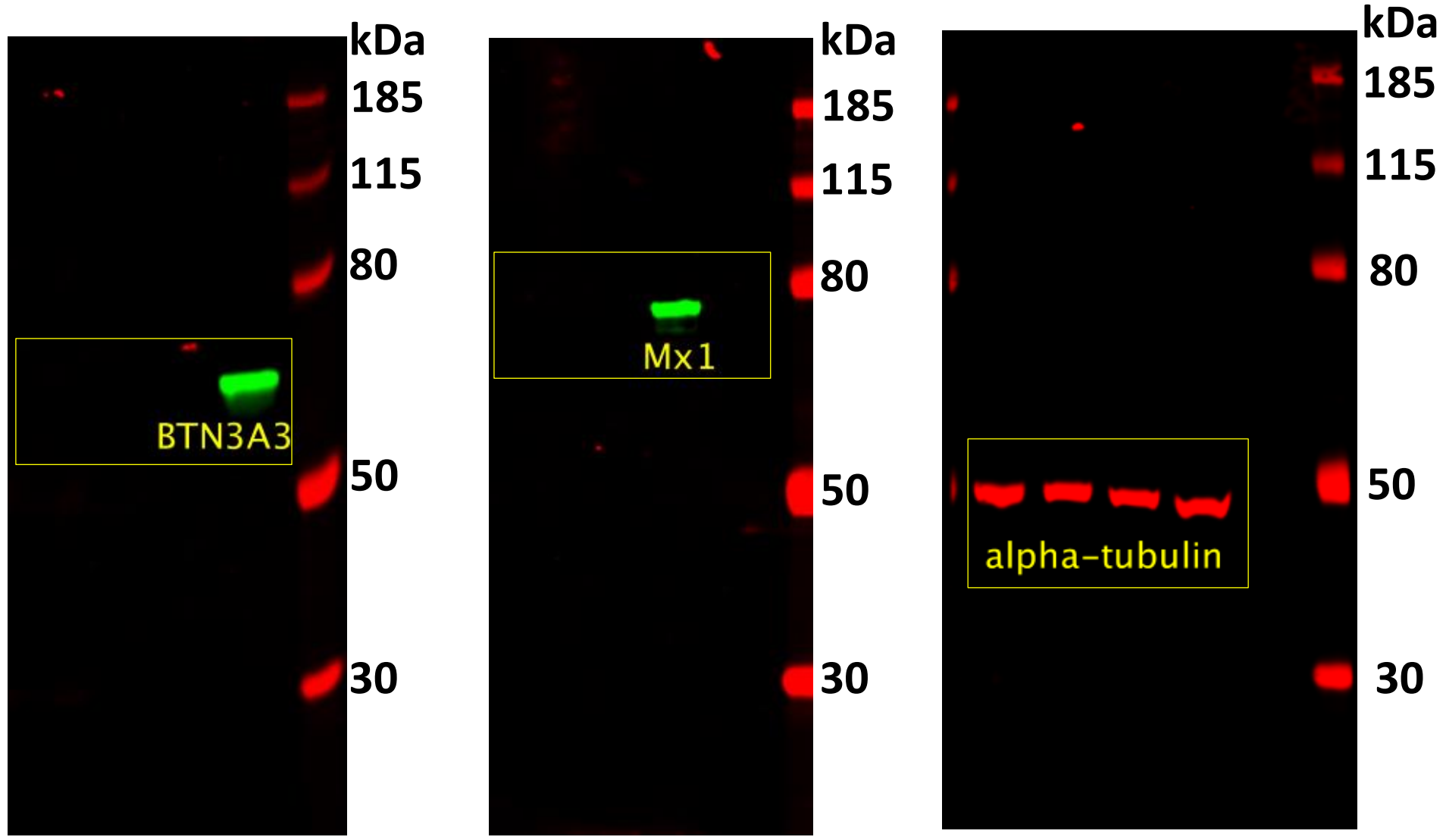

The molecular weight markers used for western blots is **PageRuler™ Plus Prestained Protein Ladder, 10 to 250 kDa (Thermo Scientific™, 26619)**, unless stated otherwise. The 65 and 25kDa markers do not fluoresce as well as the other molecular weight markers when imaged on the LICOR CLx Odyssey and have therefore not been depicted in any of the original scans. The brightness/contrast of certain original scans has been increased to highlight the molecular weight markers.
In certain blots [**Figure 4a (45 minutes fractionation) – PB1, BTN3A3 & Extended Data Figure 9a – PB1**] the signal from the red channel is stronger than the signal from the molecular weight markers. Consequently the molecular weight markers are not visible on these blots.

Figure 4a - 45 minutes fractionation

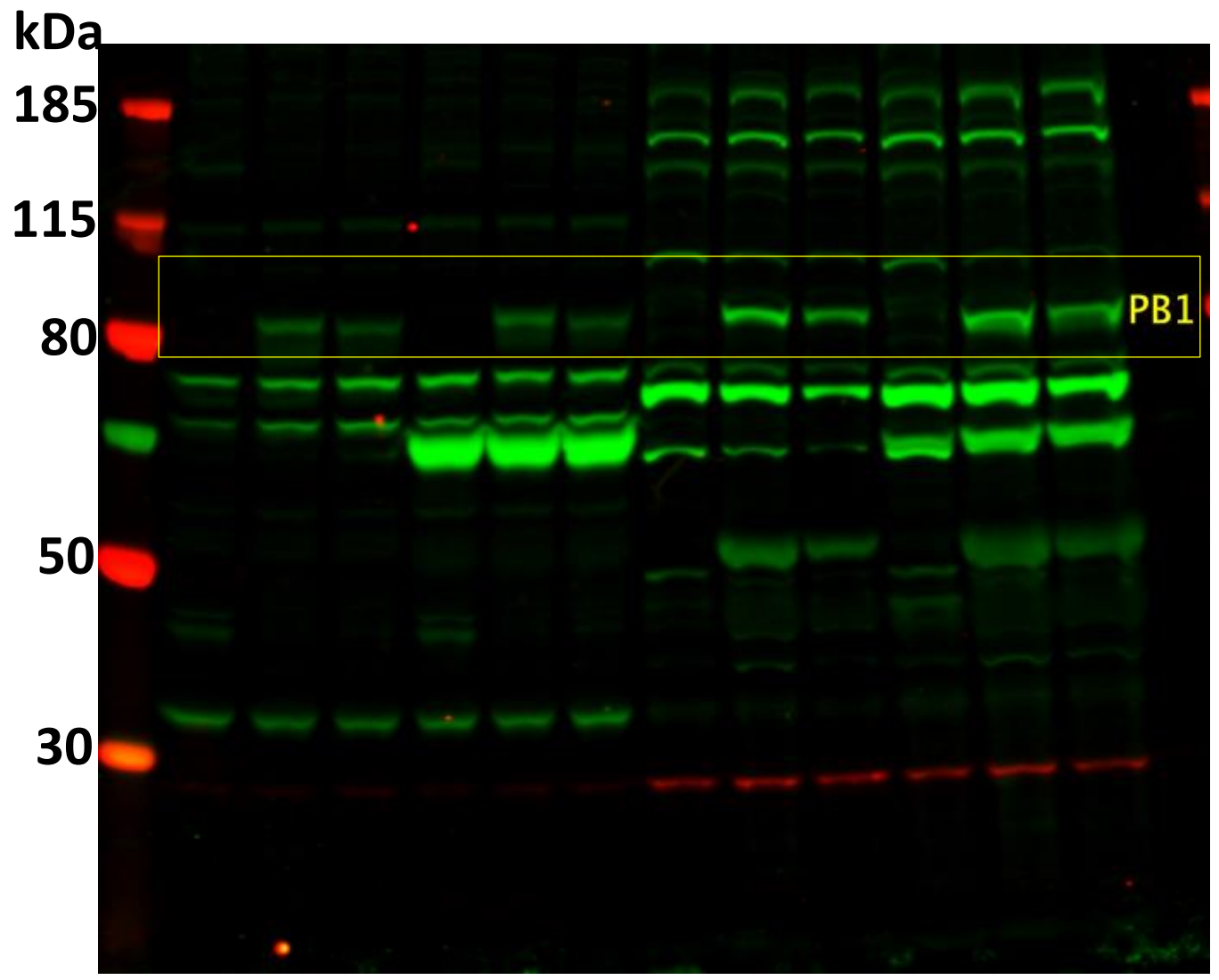

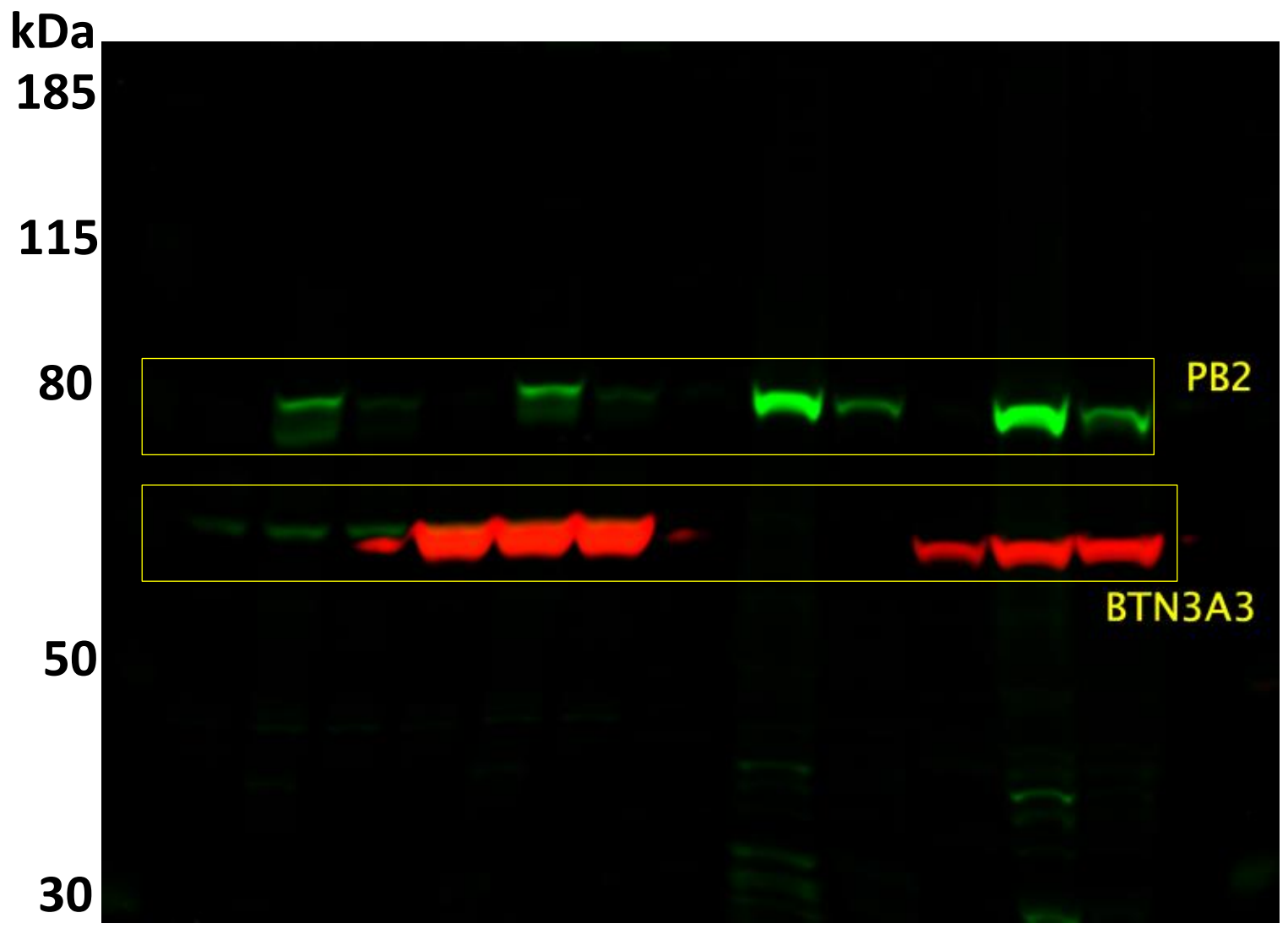

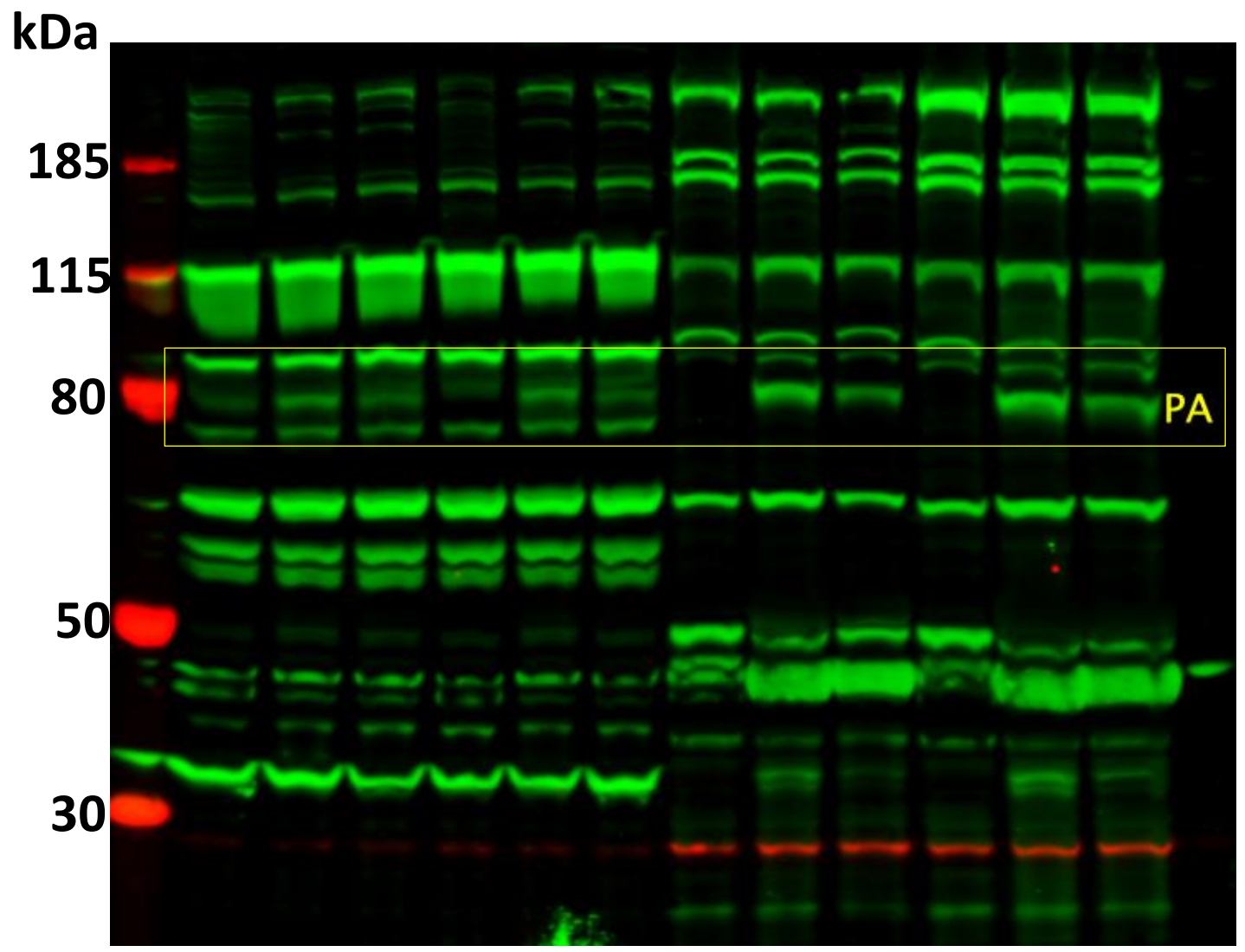

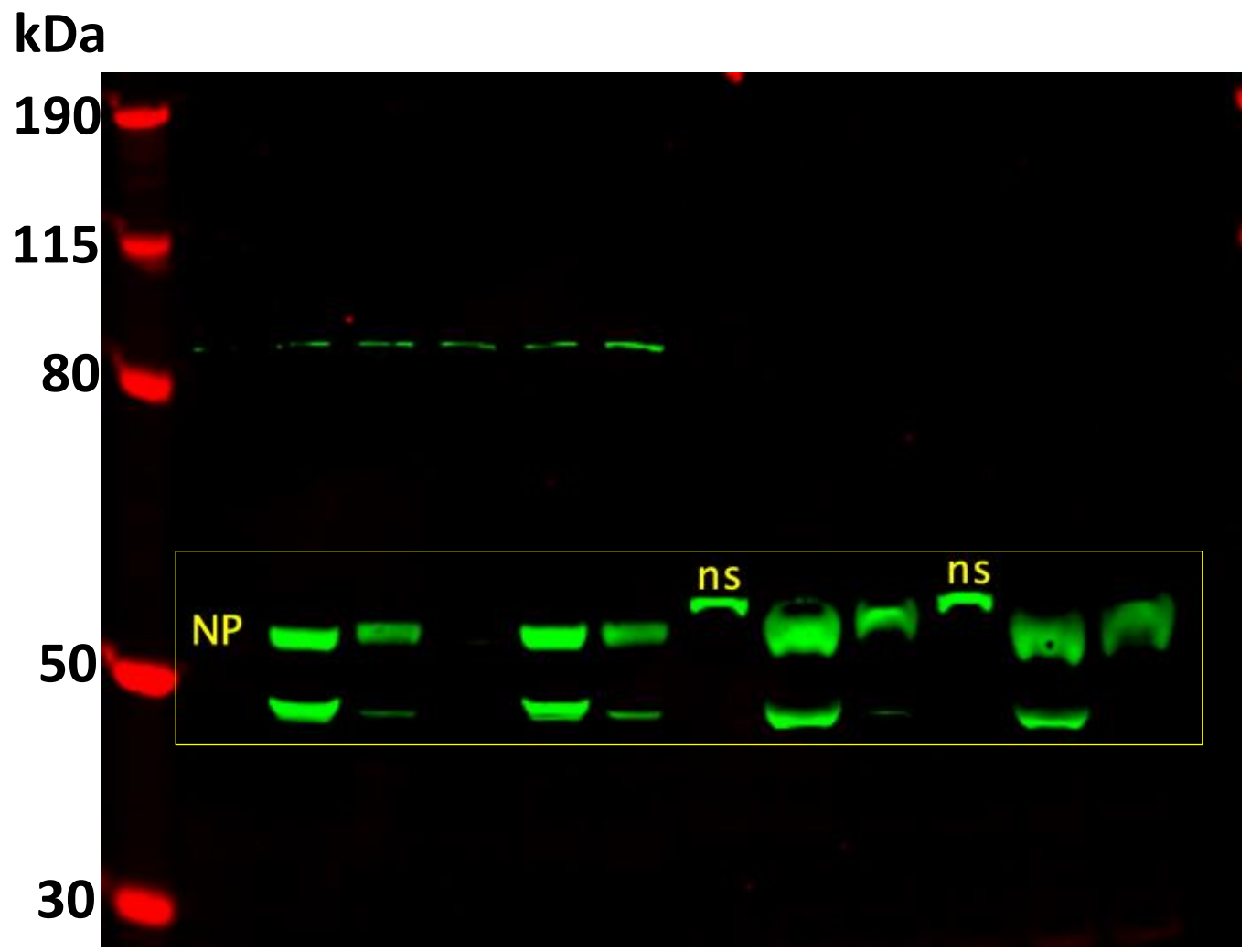

Gel run using MES buffer

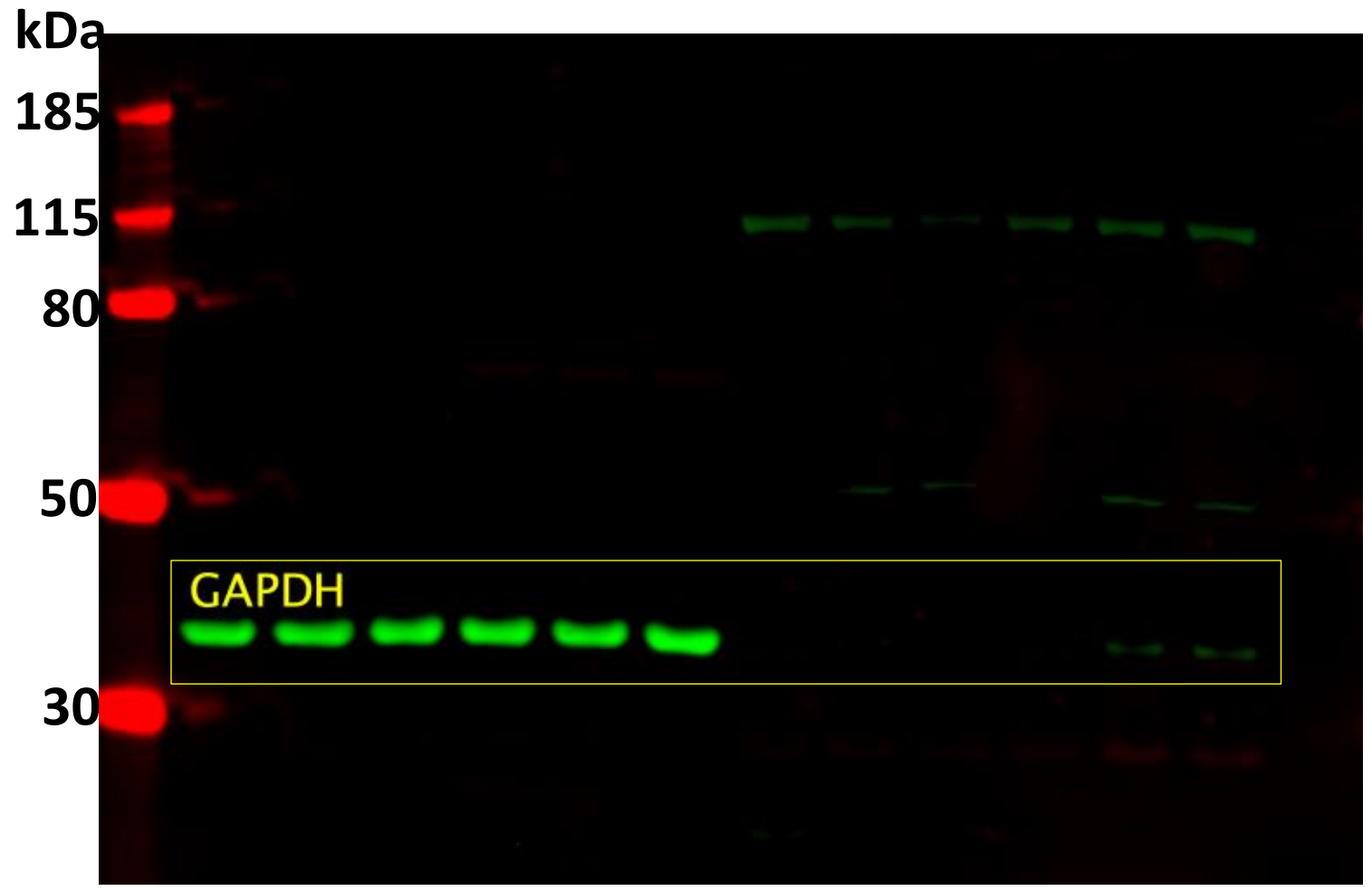

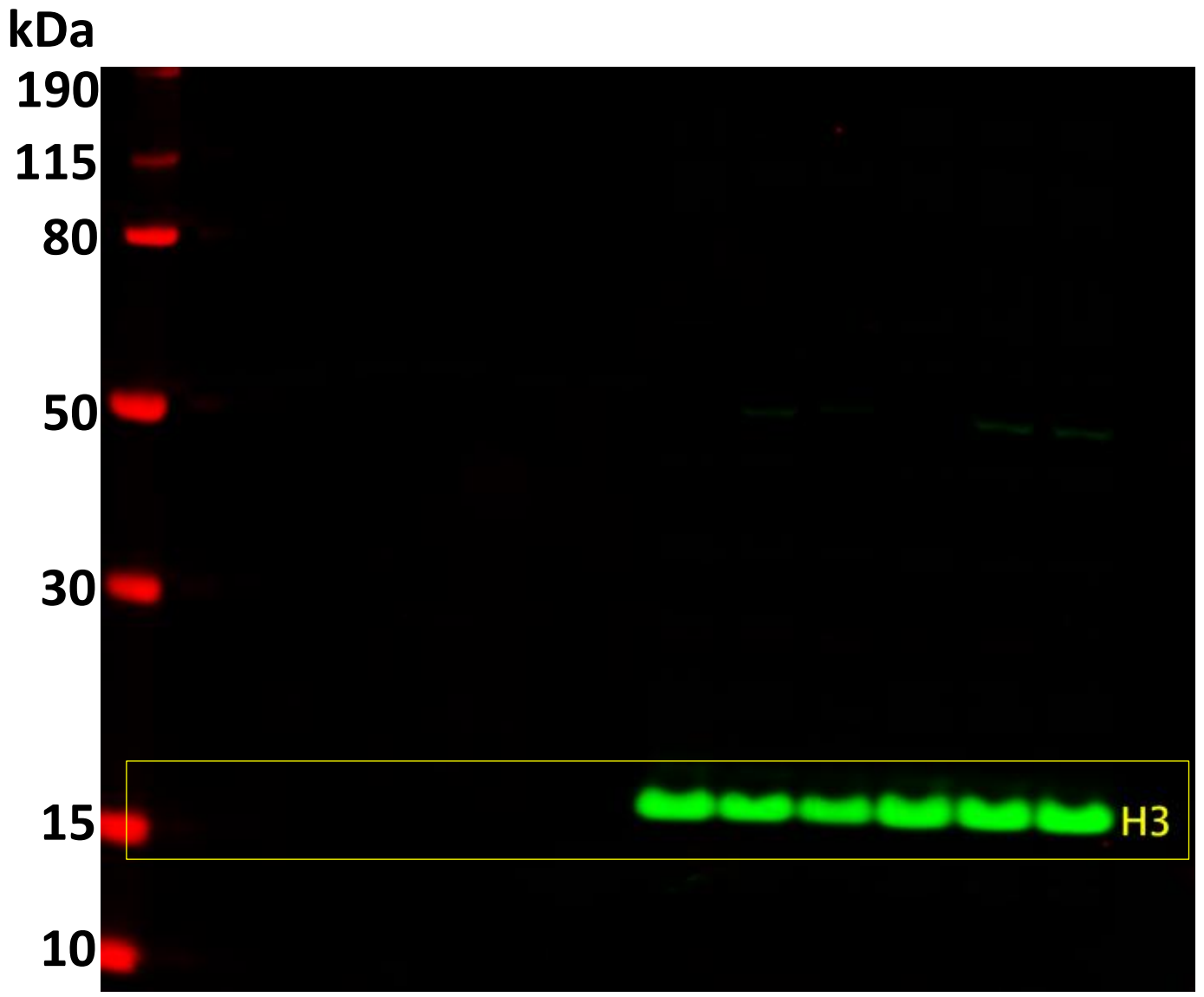

Gel run using MES buffer

Figure 4a - 90 minutes fractionation

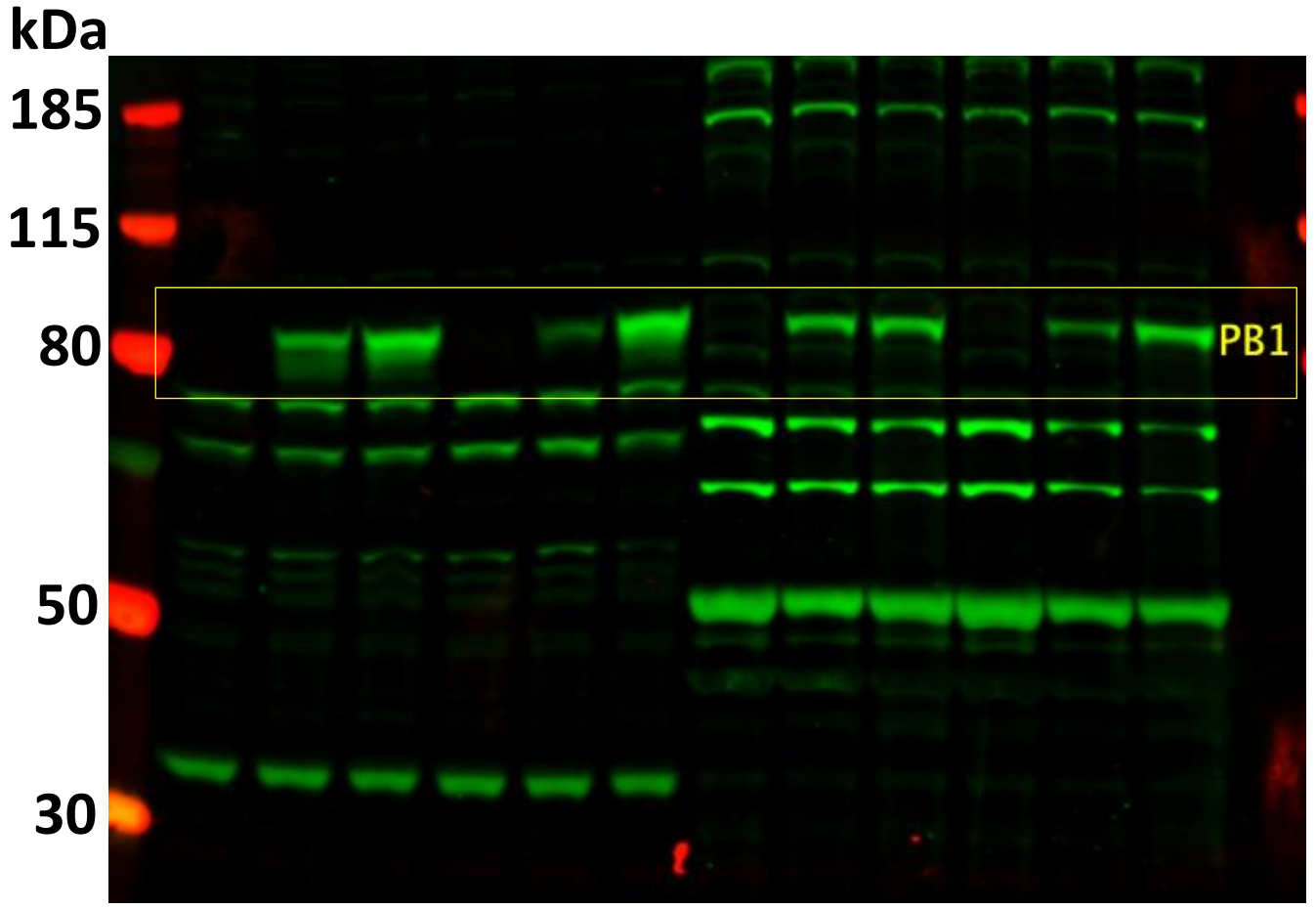

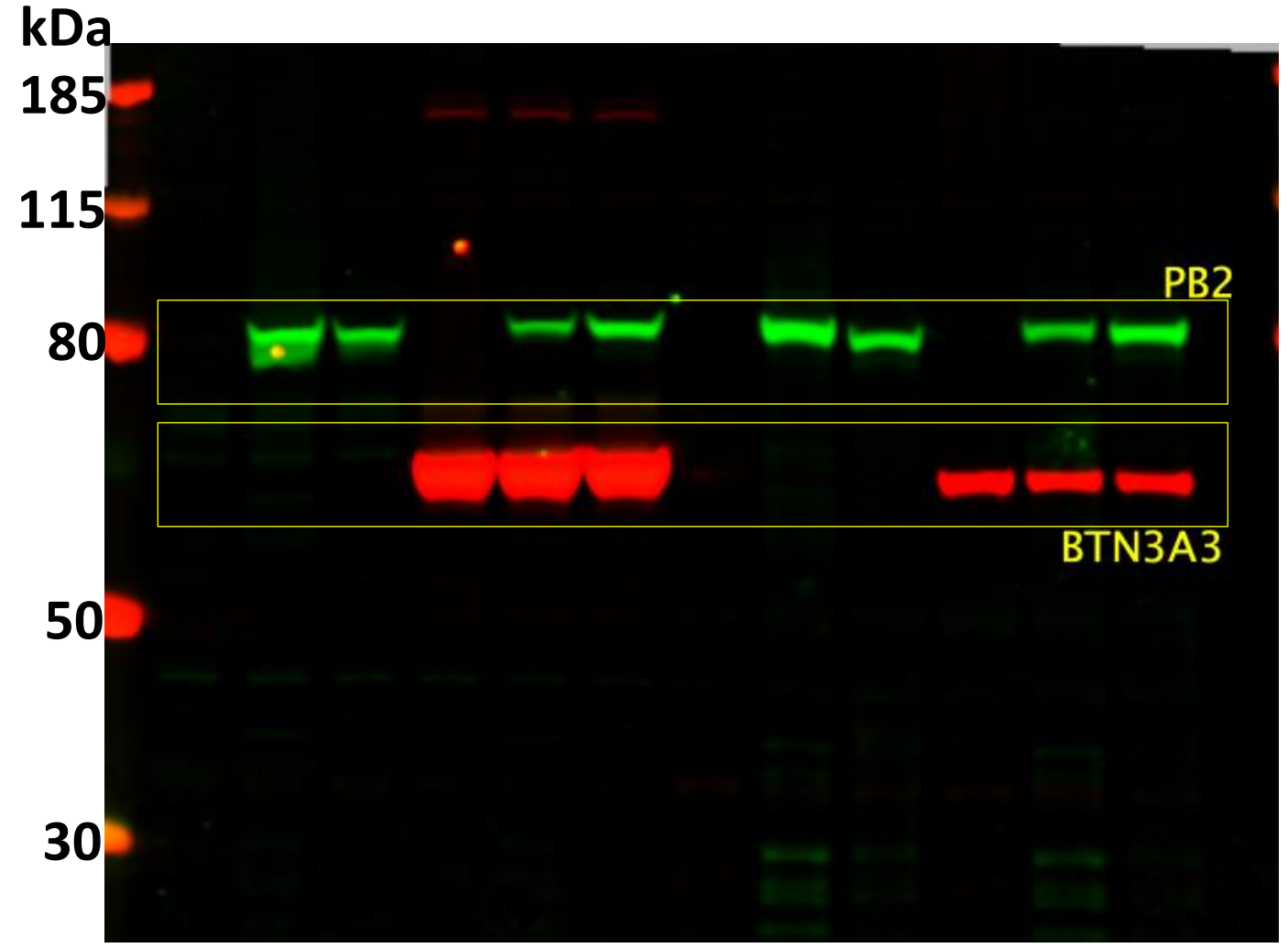

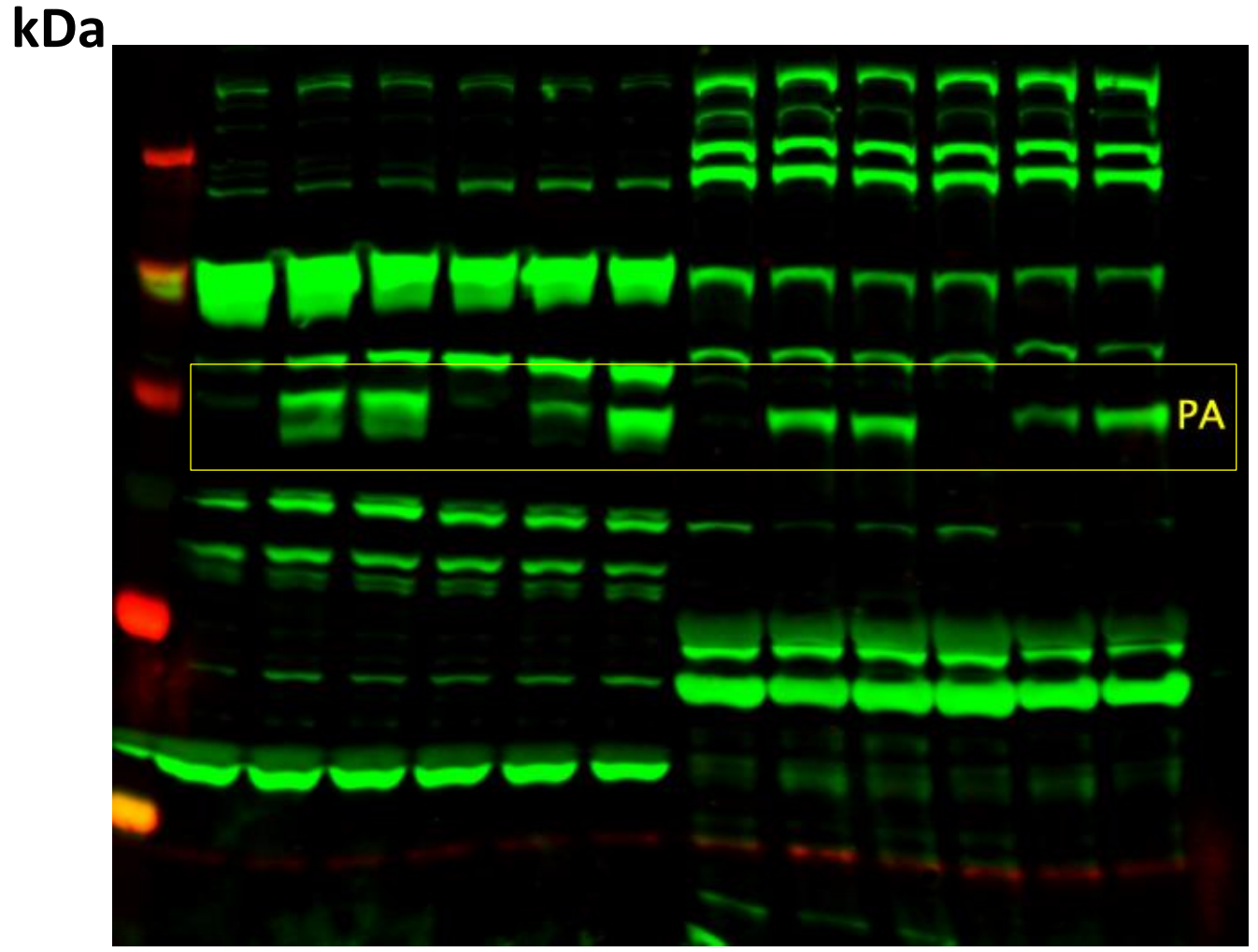

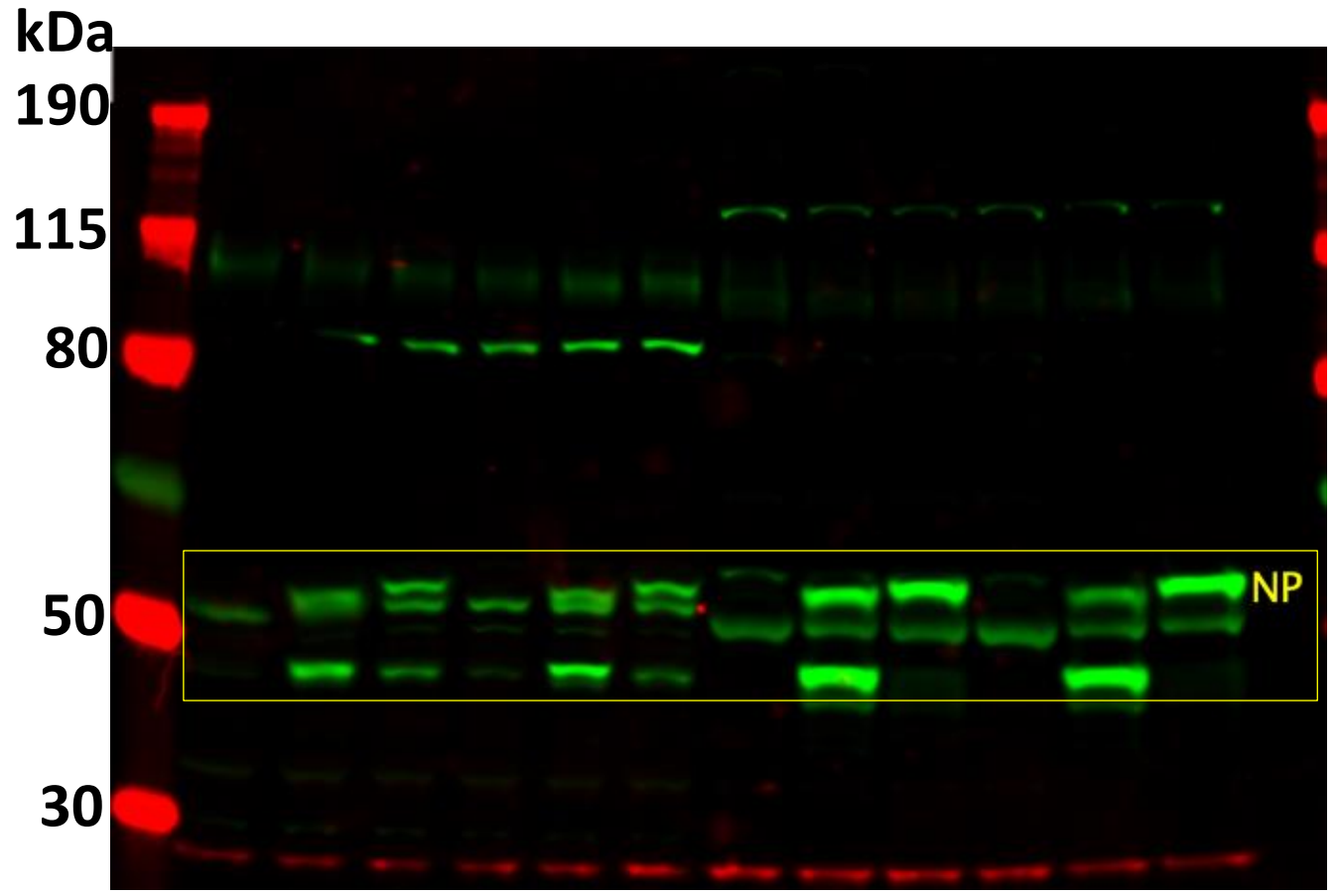

Gel run using MES buffer

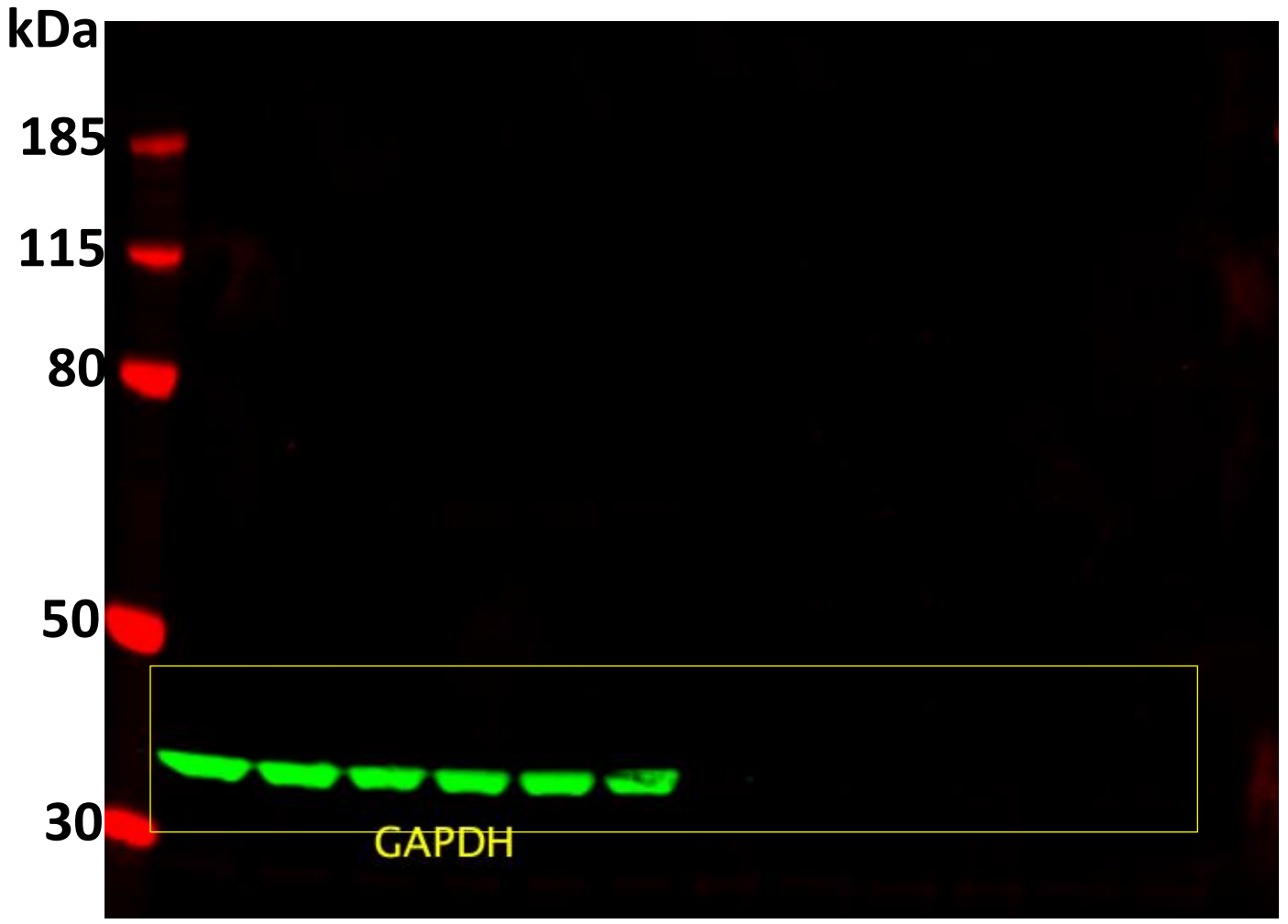

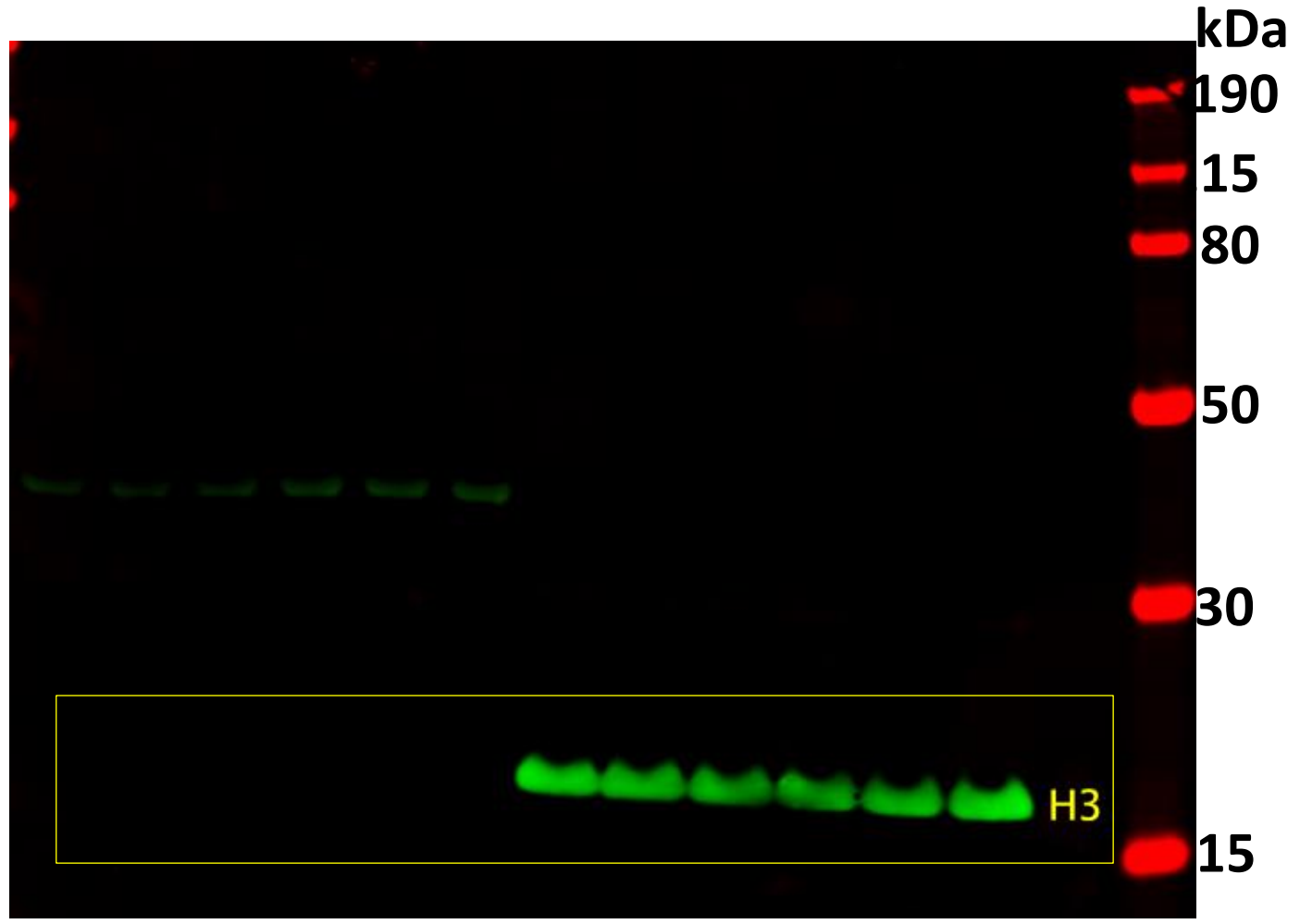

Gel run using MES buffer

Figure 4a - 6 hours fractionation

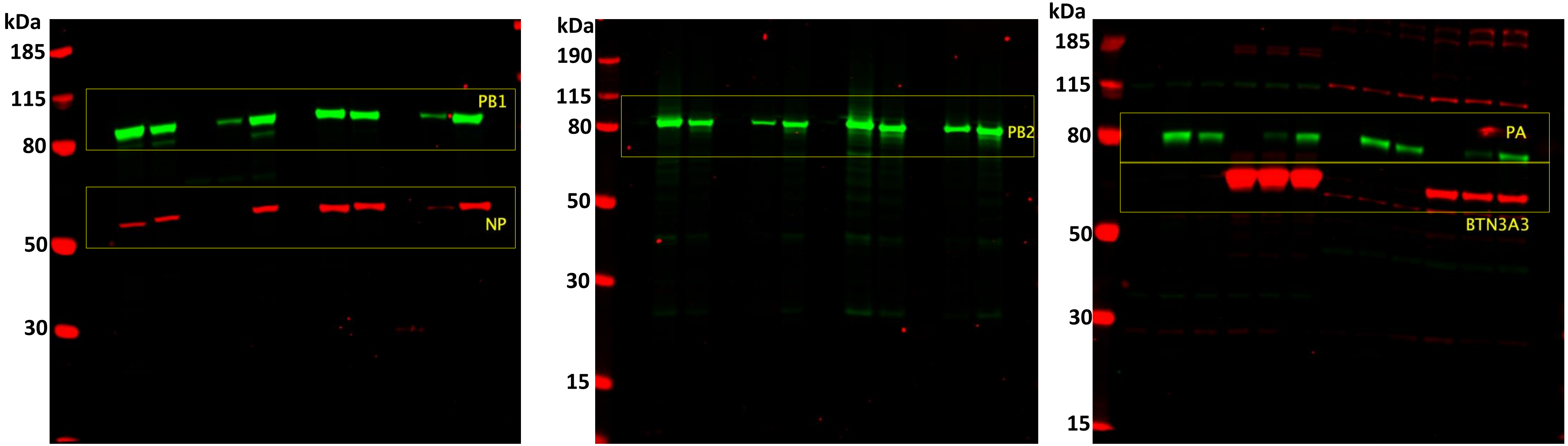

Gel run using MES buffer

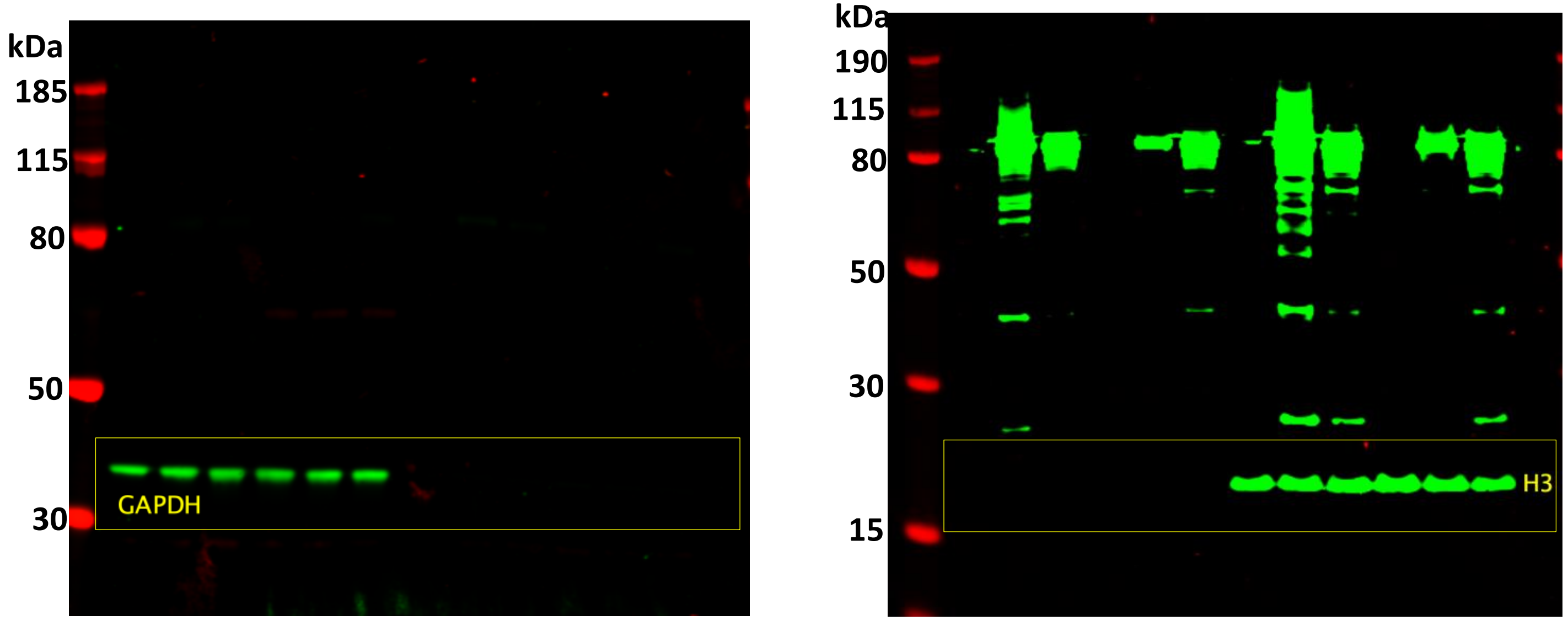

Gel run using MES buffer

Figure 4c – PR8

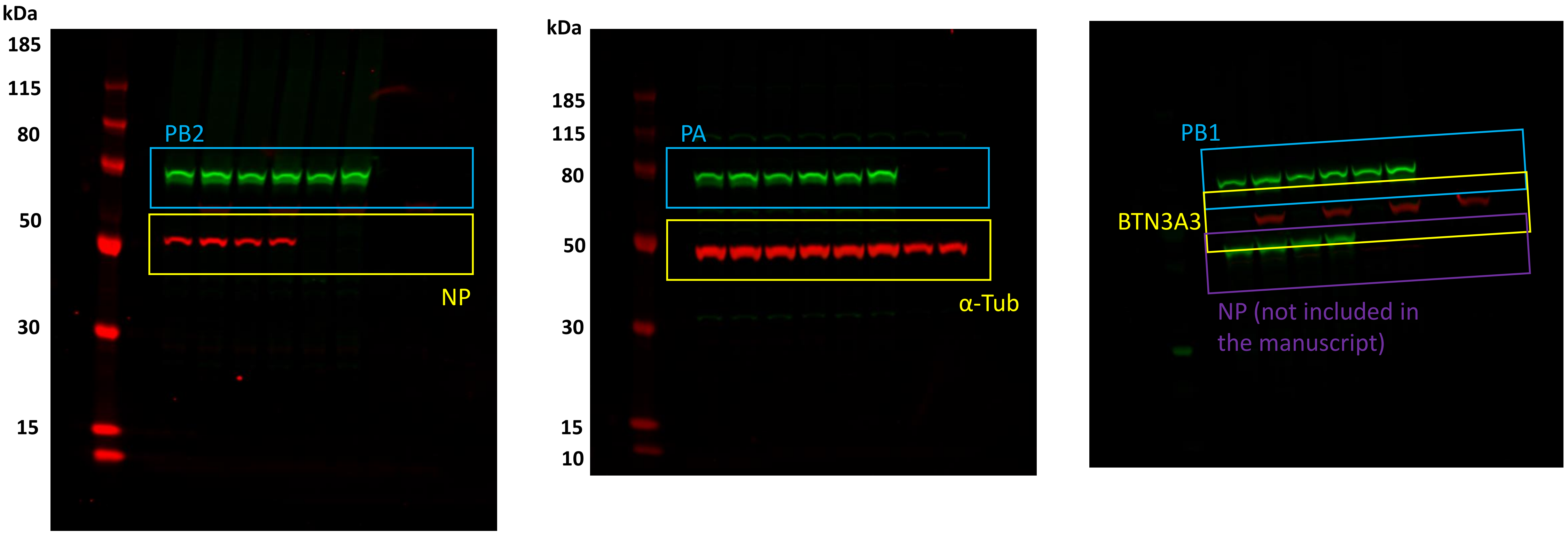

Figure 4c – Mallard

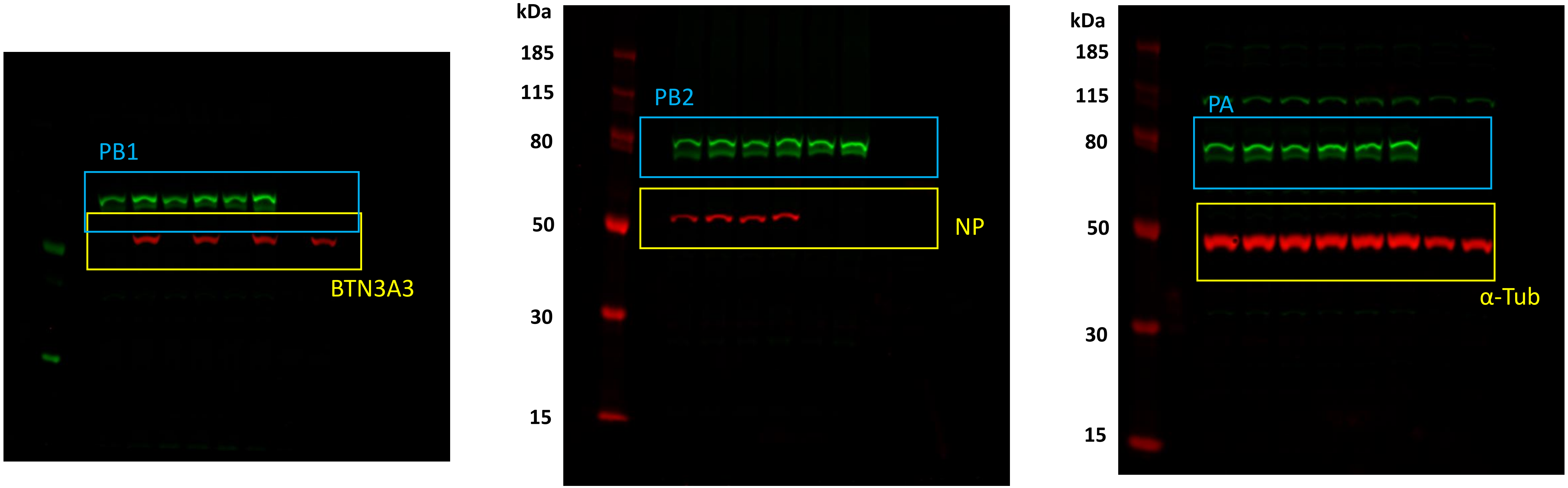

Figure 4c – Cal04

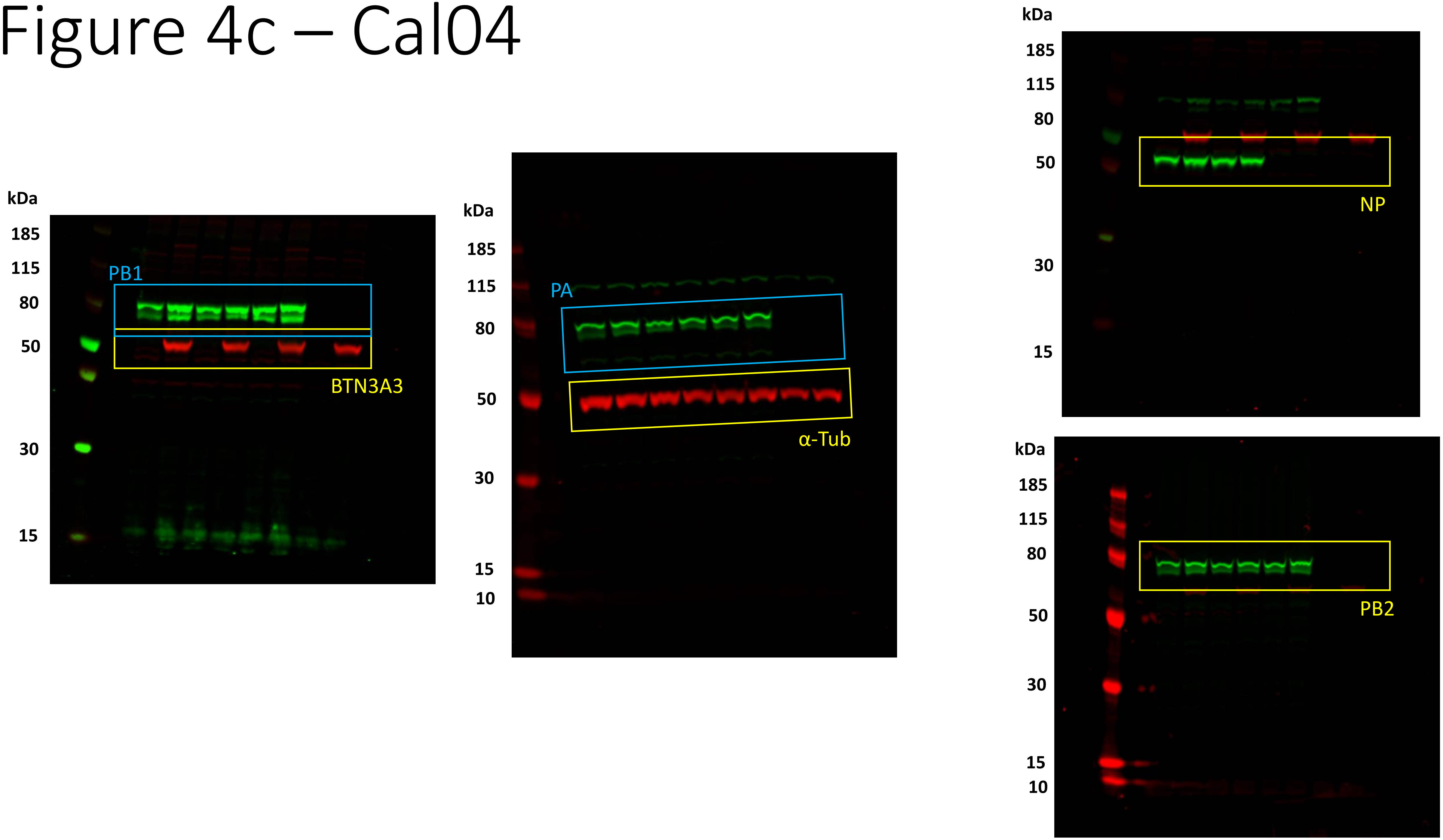

Extended Data Figure 1e – A549

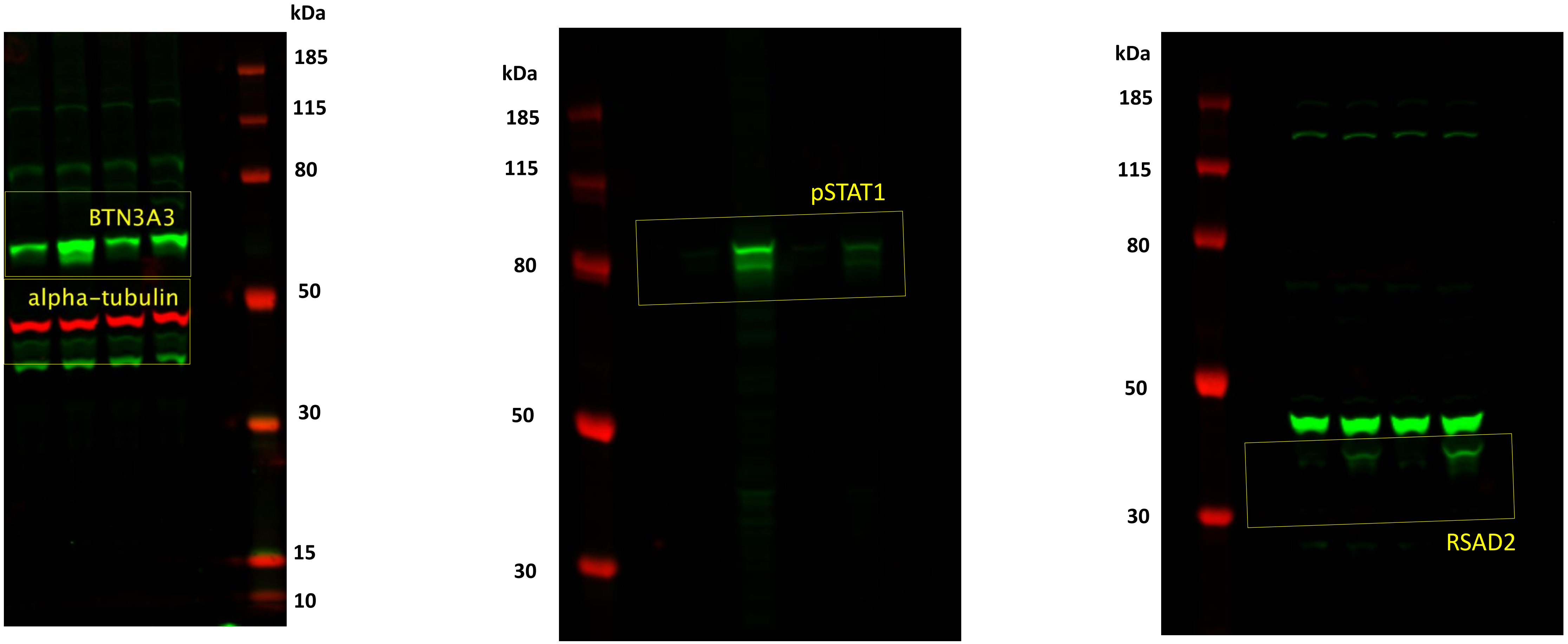

Extended Data Figure 1e – MRC5T

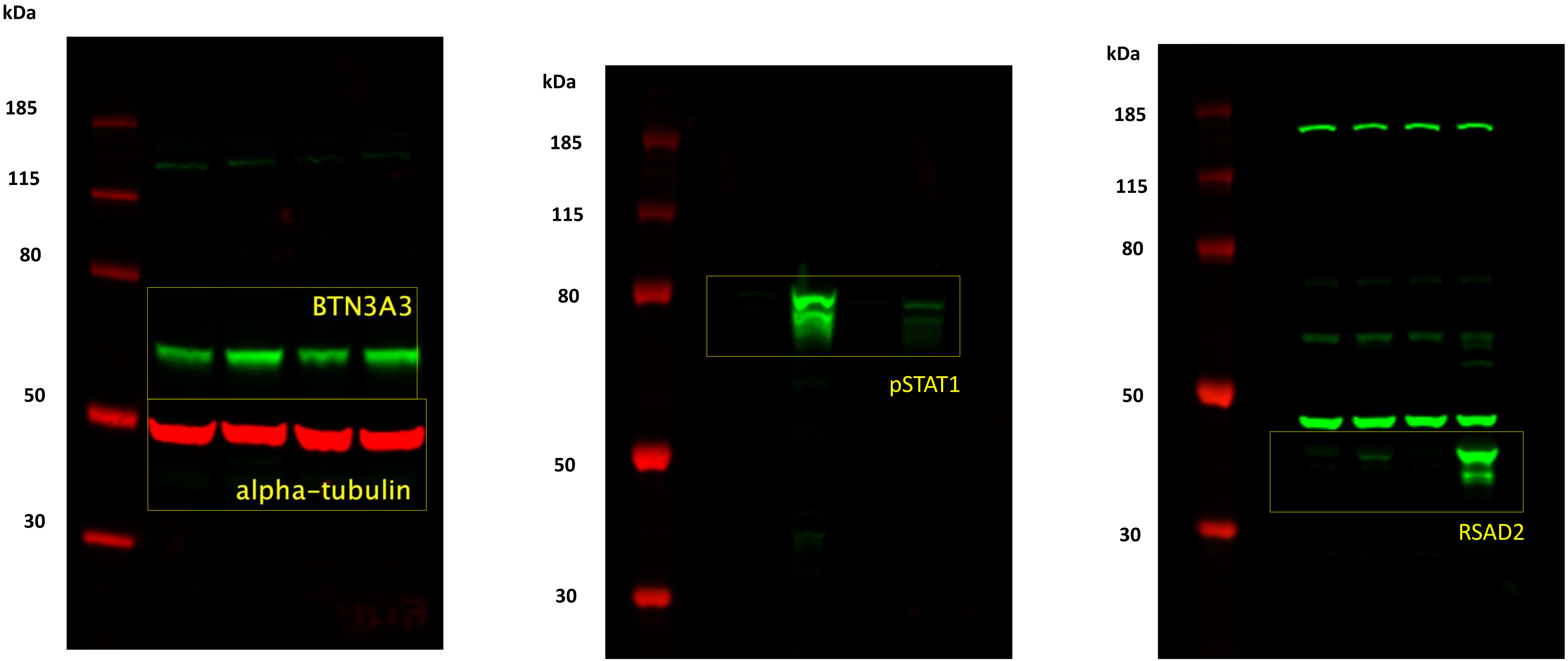

Extended Data Figure 1e – hBEC3KT

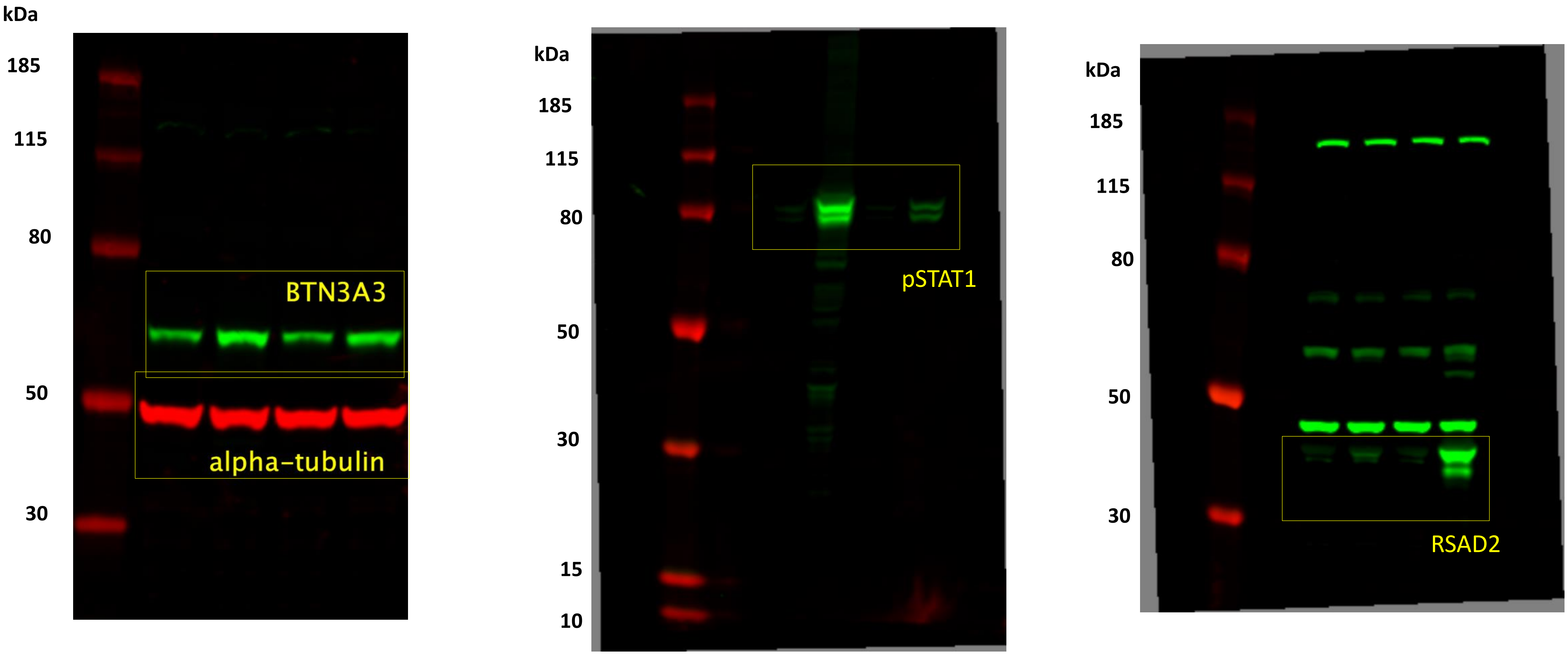

Extended Data Figure 1e – Calu3

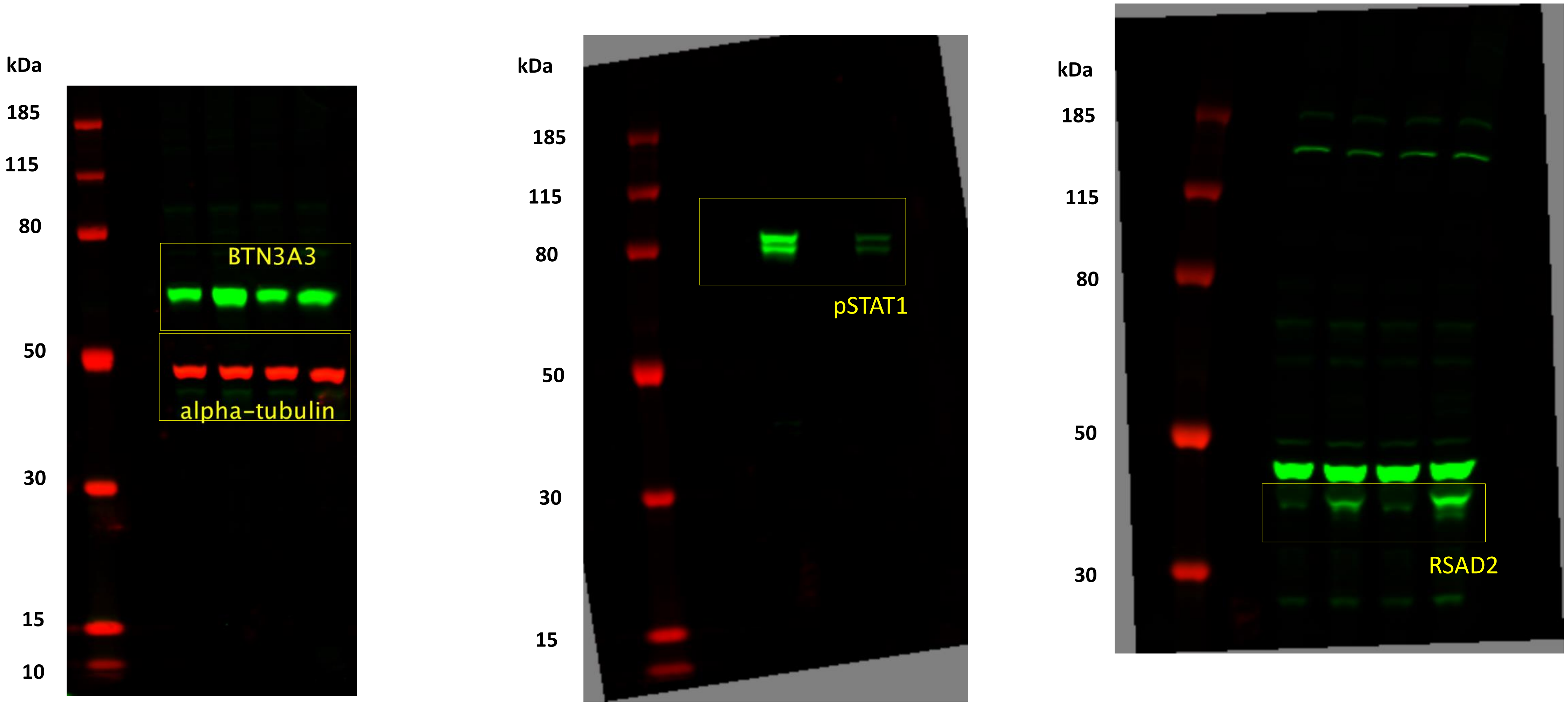

Extended Data Figure 3b

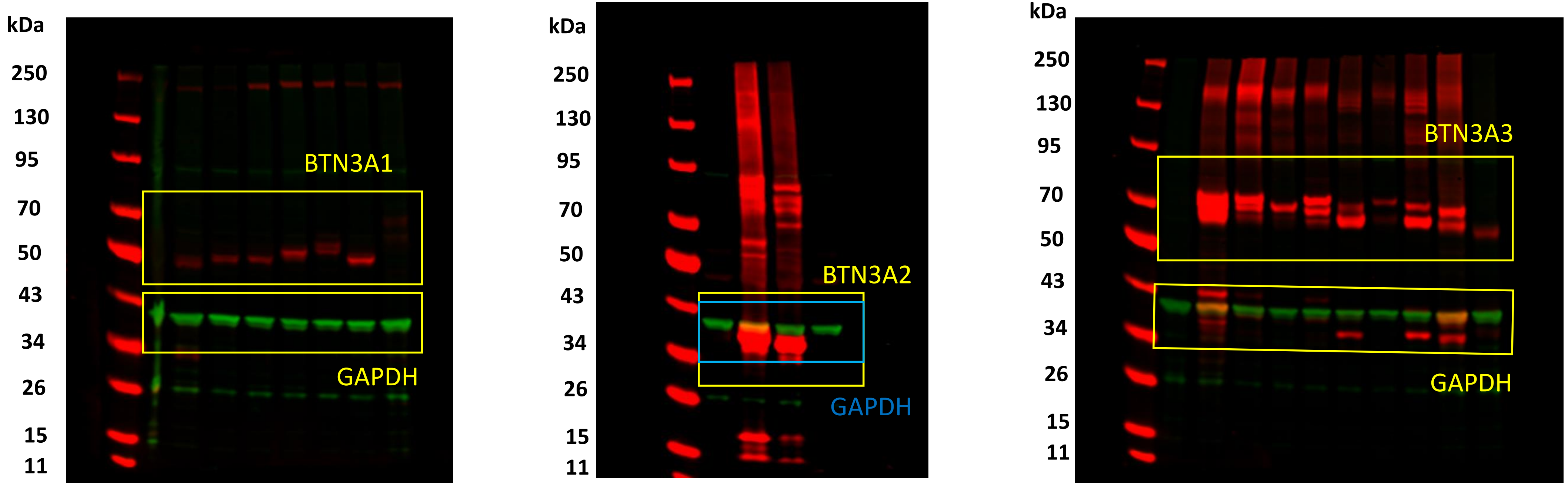

Extended Data Figure 7a

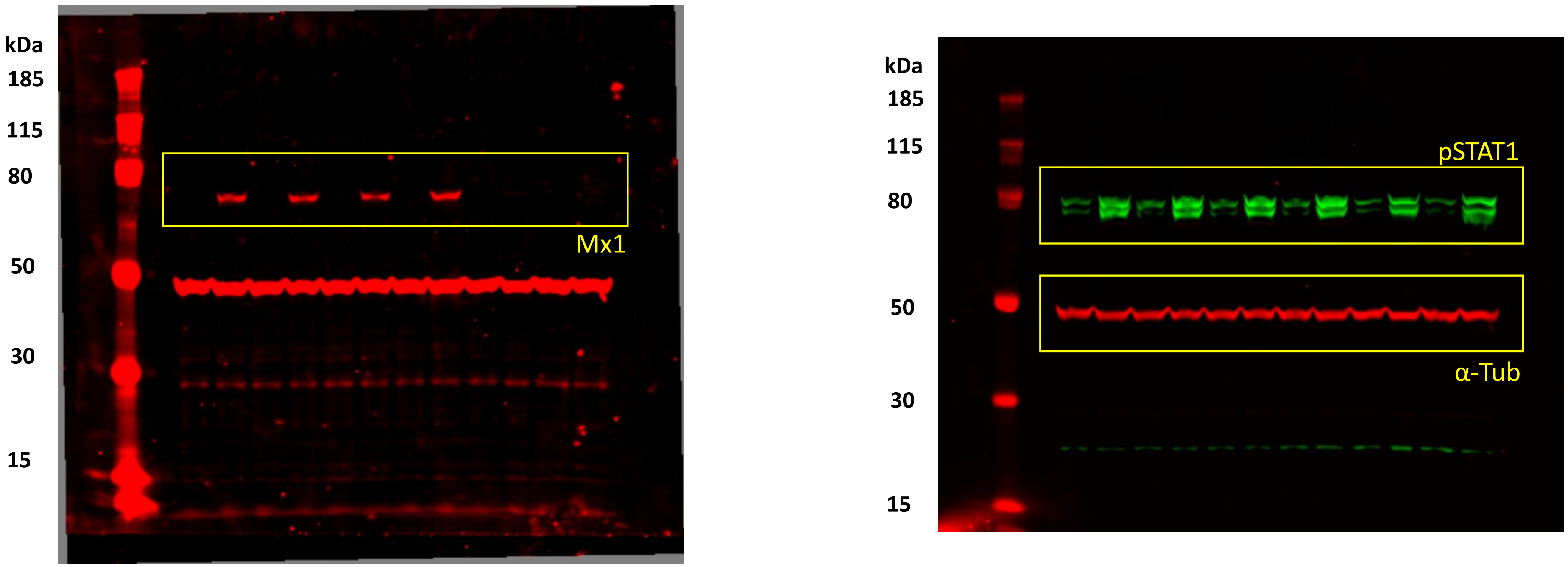

Extended Data Figure 8b – PR8:MaII

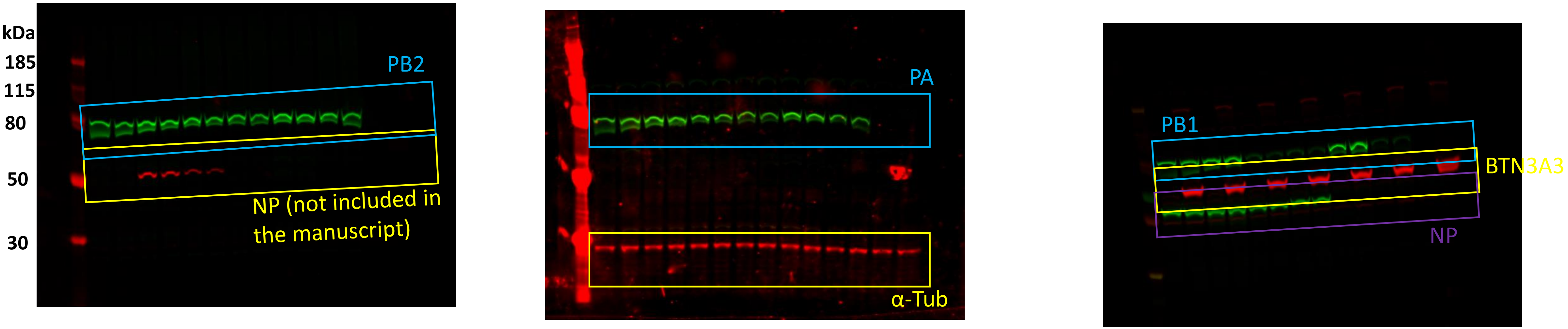

Extended Data Figure 8b – Cal04:Mall

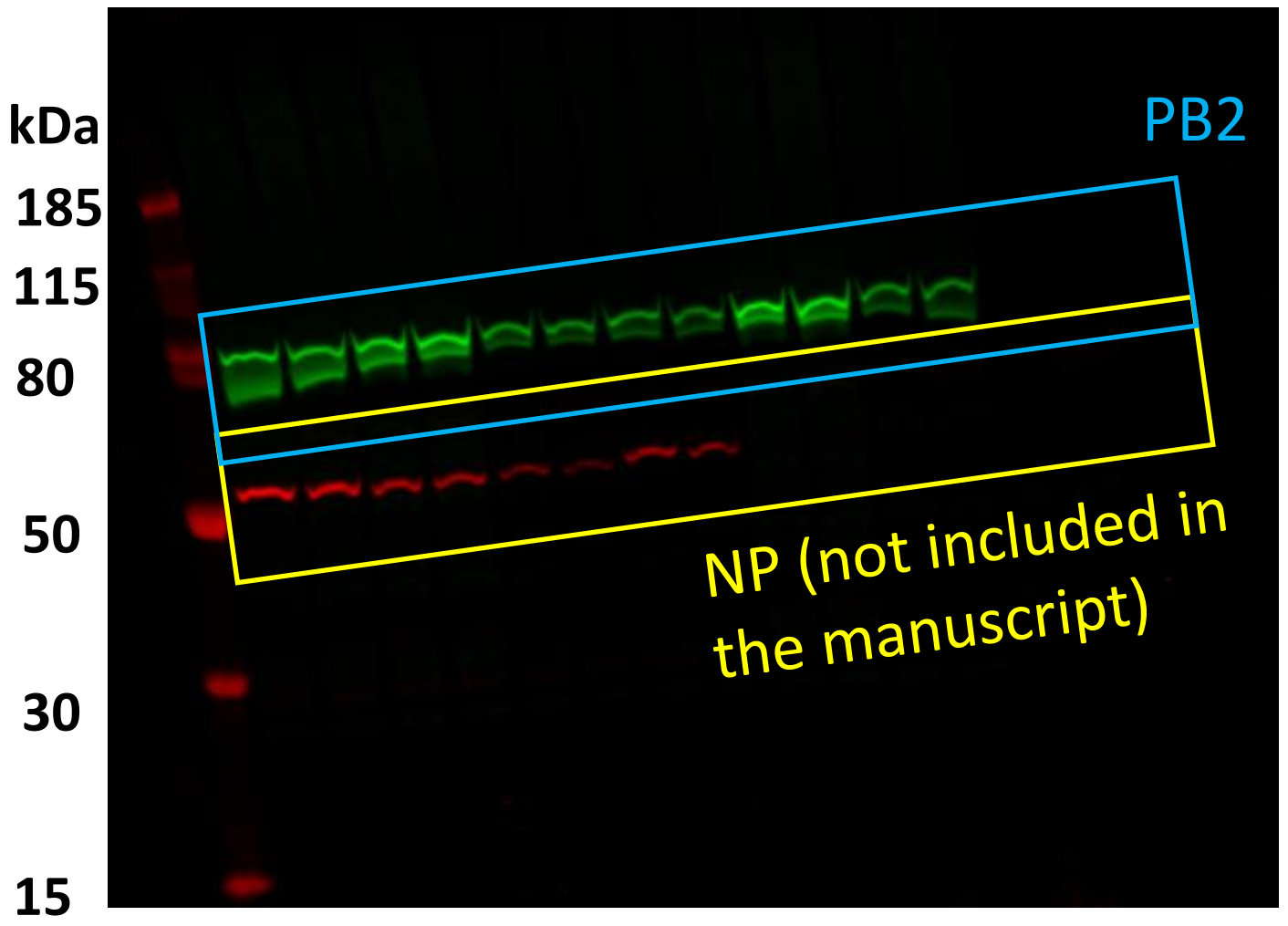

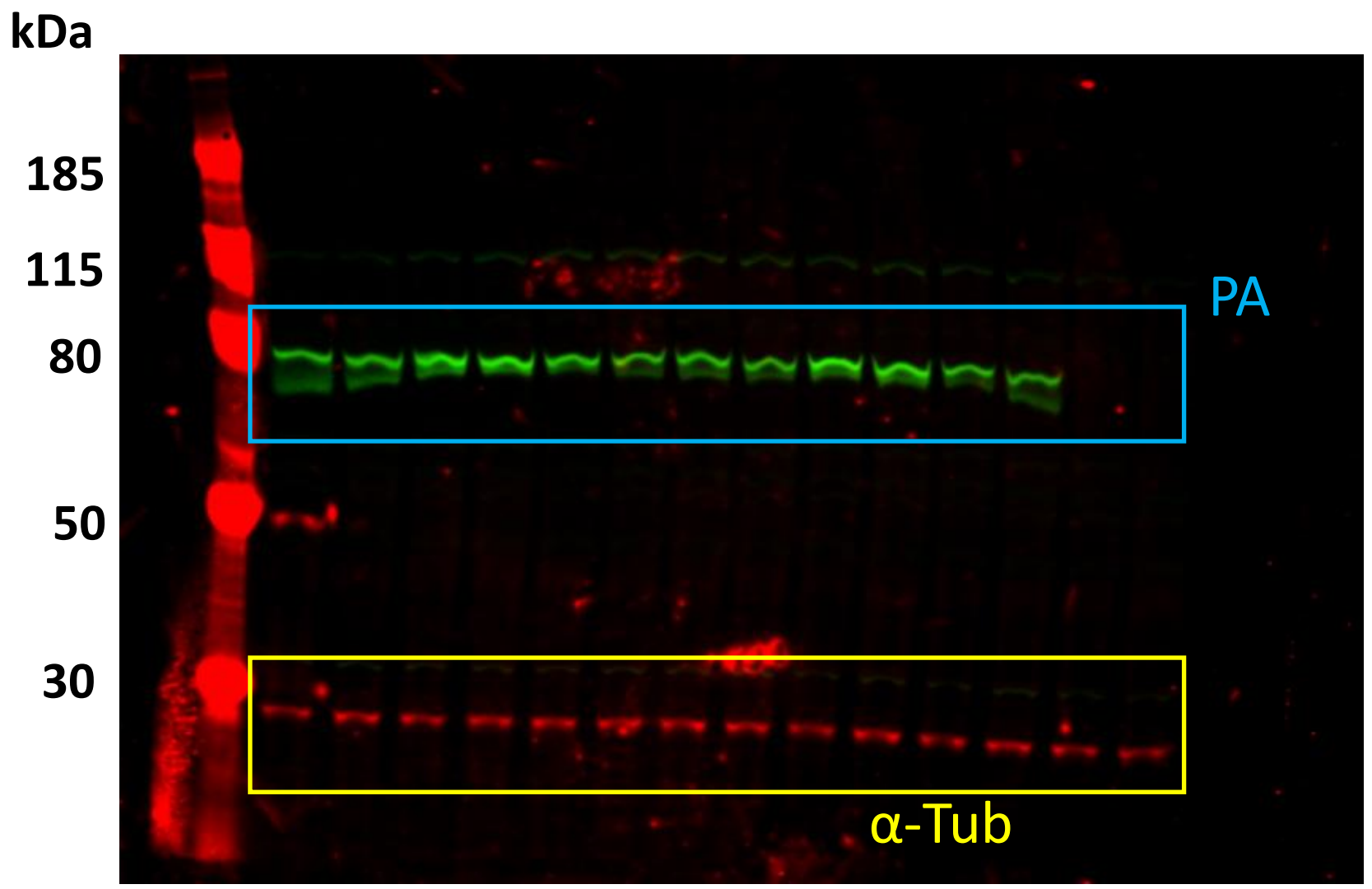

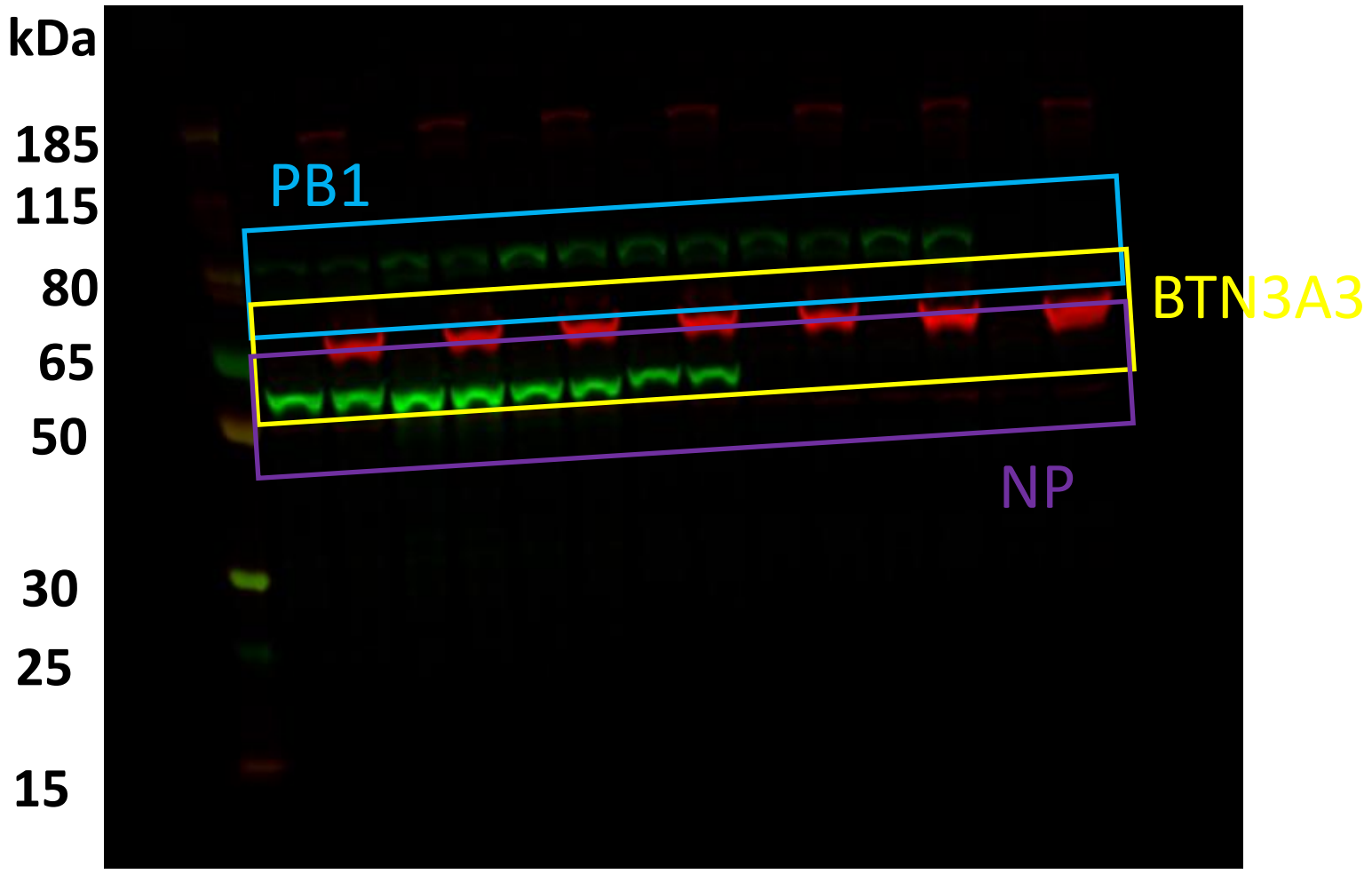

Extended Data Figure 9a

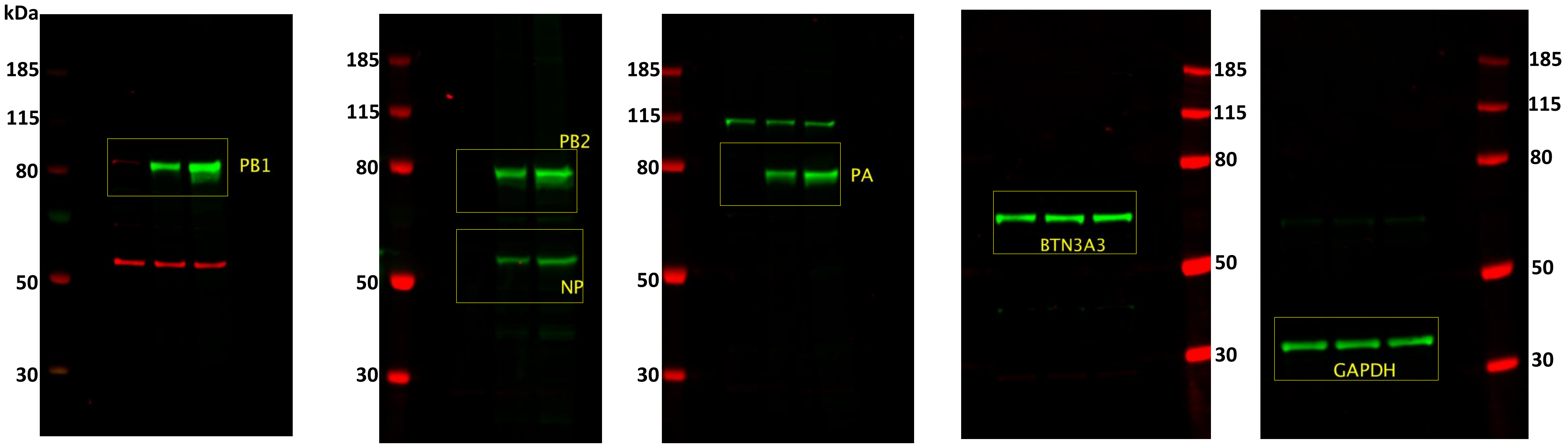

Extended Data Figure 9b

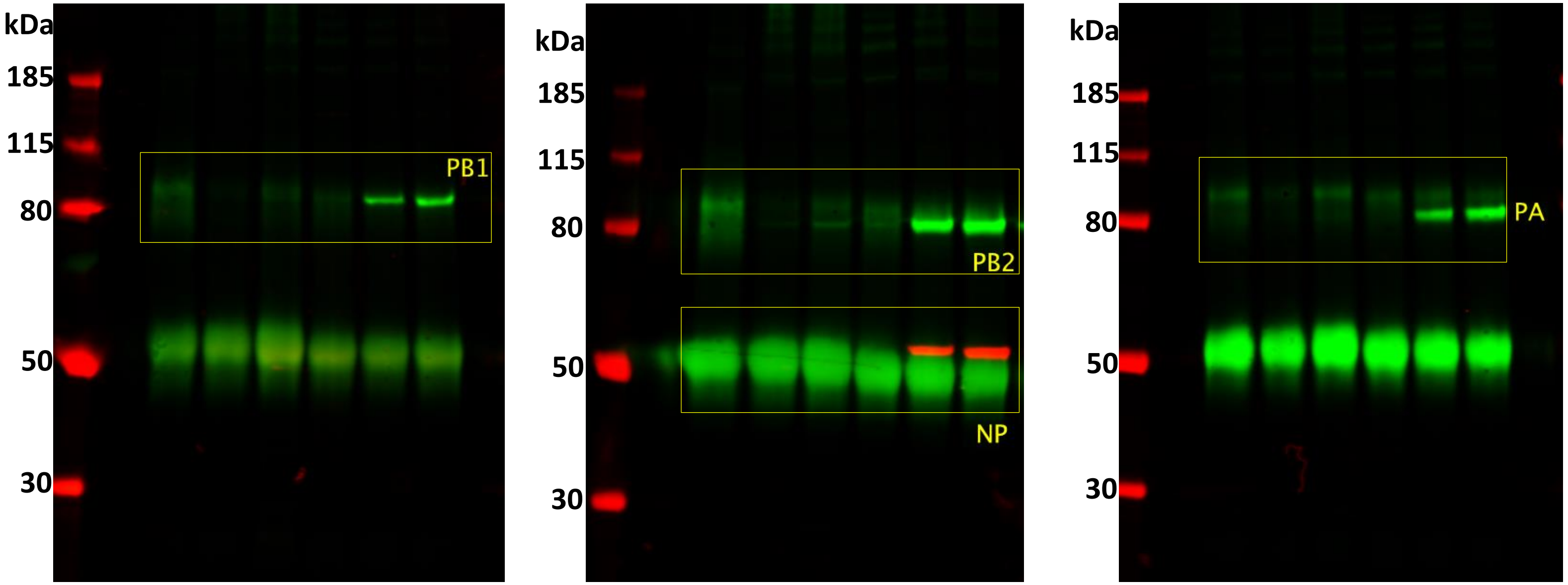

Extended Data Figure 9c

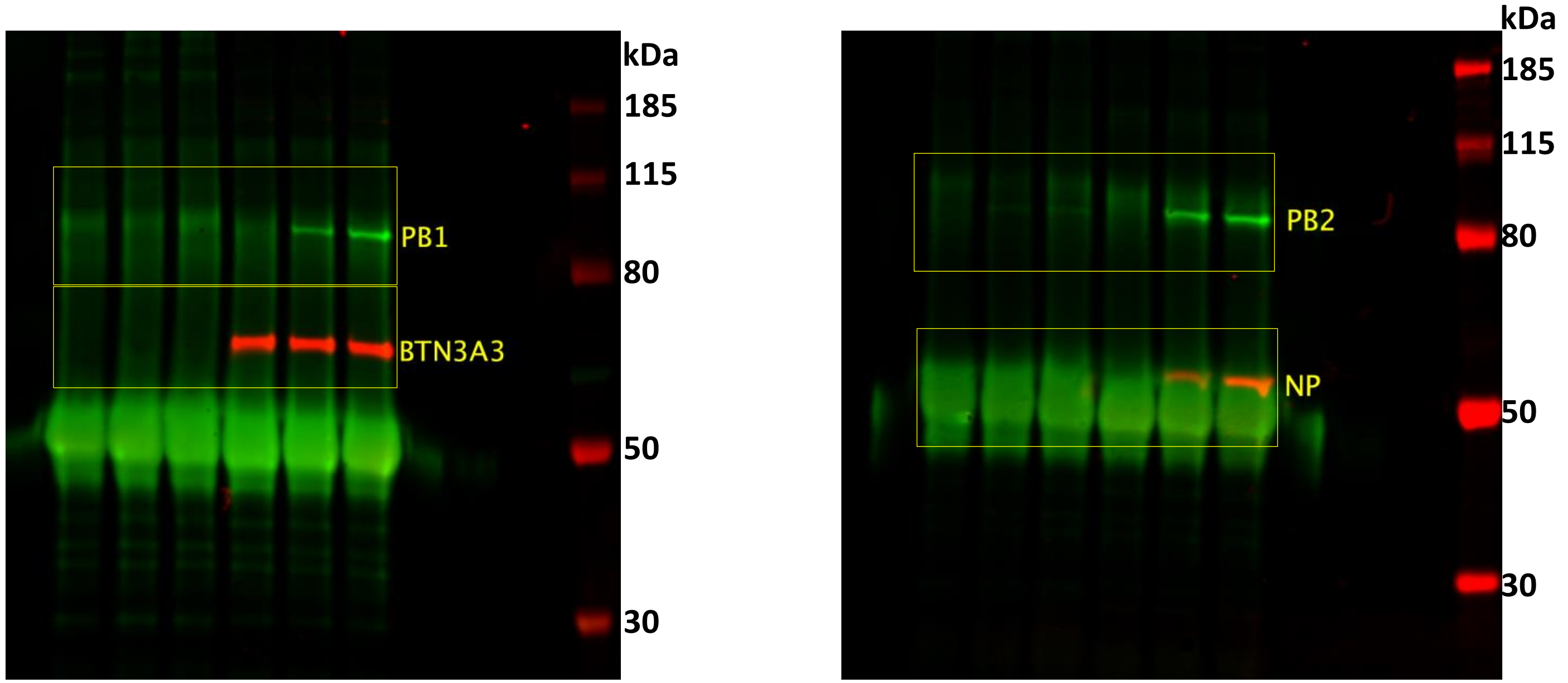

Supplement: Supplementary file 4 [file EMS219322-supplement-Supplementary_Figure_1.pdf]
