# Supplementary material for: BTN3A3 evasion promotes the zoonotic potential of influenza A viruses
Source: Nature. Author manuscript; Available in PMC 2026 Oct 15. (PMC7619554; doi:10.1038/s41586-023-06261-8)
Supplement: Supplementary file 5 [file EMS219322-supplement-Supplementary_Figure_2.pdf]

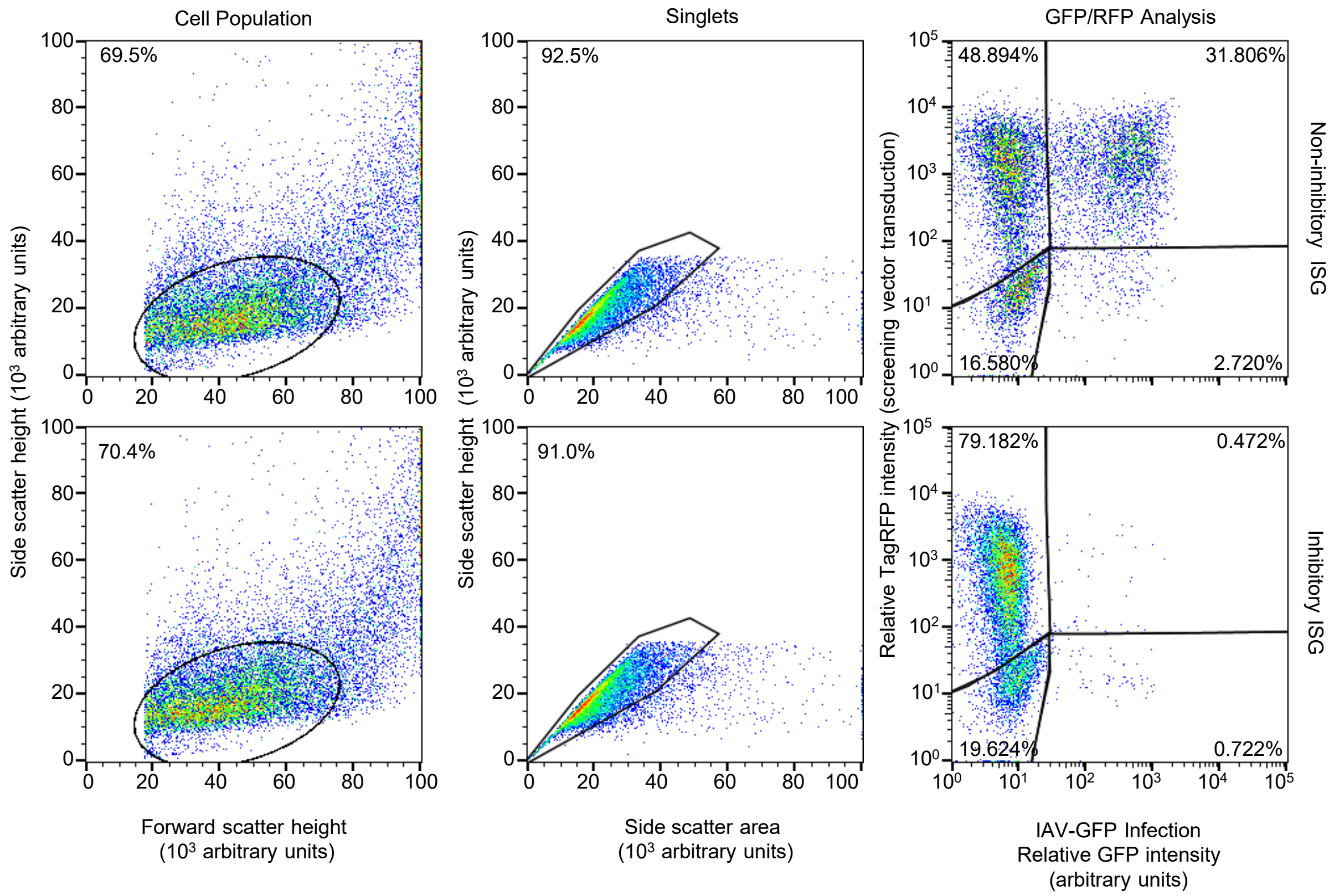

Supplementary Figure 2. **Example flow cytometry gating of an IAV-GFP arrayed ISG expression screen**. Population gating (FlowJo) of an example of a non-inhibitory ISG (top row) and a candidate antiviral ISG (bottom row) is shown. The cell population was identified using forward and side scatter height intensity (leftmost plots) with a forward scatter height threshold applied. Single cells within the cell population (singlets) were then identified for further analysis using side scatter area and side scatter height (middle plots). TagRFP (a surrogate marker for ISG expression (indicating SCRPSY ISG-encoding lentiviral vector transduction) and GFP (indicating IAV-GFP infection and gene expression) intensities of the singlet events were used to identify four populations. LL: TagRFP$^-$/GFP$^-$ (not transduced and not infected), UL: TagRFP$^+$/GFP$^-$ (SCRPSY transduced and not infected), LR: TagRFP$^-$/GFP$^+$ (not transduced and IAV-GFP infected) and UR: TagRFP$^+$/GFP$^+$ (SCRPSY transduced and IAV infected). The percentage of events in each region is shown. IAV-GFP infection in the presence of each ISG was calculated by dividing the number of events in the UR region (transduced and infected) by the number of transduced events (UL+UR). This strategy was used for the primary screens in Figs. 1b and 2e, in addition to assays where IAV-GFP infected cells were counted in Figs. 2b,f and 3f..
